# Pyrrolo[1,2‑*a*]quinoxaline: A Combined Experimental and Computational Study on the Photophysical Properties of a New Photofunctional Building Block

**DOI:** 10.1021/acs.jpcb.5c01810

**Published:** 2025-06-01

**Authors:** Adarash Kumar Shukla, Savita Choudhary, Dilawar Singh Sisodiya, Ashutosh Mahale, Pravinkumar Vipparthi, Uday Kumar Togiti, Onkar Prakash Kulkarni, Anjan Chattopadhyay, Anupam Bhattacharya

**Affiliations:** † Department of Chemistry, 209298 Birla Institute of Technology and Science-Pilani (Hyderabad Campus), Hyderabad 500078, India; ‡ Department of Chemistry, 166231 Birla Institute of Technology and Science-Pilani (KK Birla Goa Campus), NH 17B, Bypass, Road, Zuarinagar, Sancoale, Goa 403726, India; § Department of Pharmacy, 209298 Birla Institute of Technology and Science-Pilani (Hyderabad Campus), Hyderabad 500078, India

## Abstract

We report a detailed photophysical exploration of medicinally important pyrrolo­[1,2-*a*]­quinoxalines. Pyrrolo­[1,2-*a*]­quinoxaline (QHH), 2,4-diphenylpyrrolo­[1,2-*a*]­quinoxaline (QPP), 2-phenyl-4-(thiophen-2-yl)­pyrrolo­[1,2-*a*]­quinoxaline (QPT), and 4-phenyl-2-(thiophen-2-yl)­pyrrolo­[1,2-*a*]­quinoxaline (QTP) were synthesized and studied using various photophysical techniques. Preliminary studies revealed the environmental responsiveness of these systems along with the involvement of aggregation-induced emission (AIE). Molecules QPP and QTP displayed significant fluorescence enhancement via AIE when compared to QHH and QPT. The fluorophores could be used for bioimaging with subcellular localization, specifically on the lysosomes. TDDFT studies revealed that both S_0_–S_1_ and S_0_–S_2_ transitions dominate the higher-wavelength peaks in the absorption spectra of the nonrigid PQNs. A complete charge delocalization was seen in the fluorescent S_1_ state of the rigid unsubstituted analogue. In contrast, in its 2,4-disubstituted analogues, electronic charge clouds on the benzene and thiophene rings adjacent to the fused pyrrole of the PQN moieties were absent. The S_1_ states of these latter nonrigid molecules undergo ISC with higher triplets (T_2_/T_3_) through further twisting along the C–C bonds, accompanied by high charge transfer with a 1.5- to 3.0-fold increase in the dipole moments. These triplets were identified as the phosphorescent states, while triplet T_1_ seems to be responsible for ROS generation, aided by aggregation. Computational results were validated by phosphorescence studies, which showed the lowest intensity for the completely unsubstituted QHH, while the highest intensity was observed in the case of QPP. Subsequent studies also revealed the ROS generation capability of these systems, as indicated by the computational results. This work establishes the versatility of the pyrrolo­[1,2-*a*]­quinoxaline core and puts forward a plausible mechanism responsible for its photophysical features.

## Graphical abstract

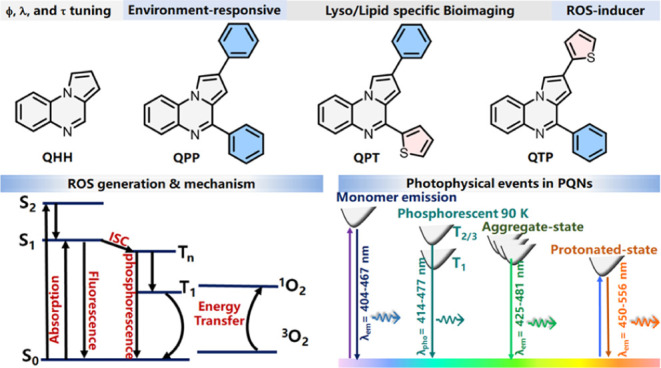

## Introduction

Pyrrolo­[1,2-*a*]­quinoxalines (PQNs), which possess a dual electron donor/acceptor nature due to a pyridine-pyrrole core, are well known in medicinal chemistry.^1–5^ However, a systematic photophysical study of this core is lacking in the literature. The few studies available in the literature have focused on using these systems as probes via fluorescence-assisted quenching in organic solvents and phosphorescent OLEDs.^6^ Only one article on the utility of these molecules for bioimaging applications is known, which reports using pyrroloquinoxaline hydrazones as fluorescent probes for studying amyloid fibrils.^7^ Additionally, most studies involving PQNs have focused on using their monosubstituted variants.^8–10^ Therefore, a thorough investigation of this scaffold with multiple substituents is required to modulate its photophysical properties and fully explore its capabilities.

Fluorophores relying on AIE characteristics have facilitated multiple domains due to their ability to turn on fluorescence, high Stokes shift, high photobleaching threshold, low cytotoxicity, and excellent bioimaging efficiency. The AIE phenomenon occurs in extended π-conjugated molecules, with most examples comprising core structures such as α-cyano-1,4-distyrylbenzenes, tetrabenzeneethenes, and 9,10-divinylanthracenes.^11–13^ In addition, strategies like the incorporation of lengthy alkyl chains/boron units and modulating fwhm (full width at half-maximum) are also used frequently to access the desired emission wavelengths from the aggregates.^14^ Finding small π-conjugated structures capable of AIE is difficult due to π–π stacking leading to quenching.^15–18^ A few examples are known where a substituent-based approach was successfully used to modulate the AIE.^19–21^

Organic phosphorescent molecules, with their long emission lifetime and good biocompatibility, have opened up many opportunities as promising bioimaging materials.^22,23^ These molecules offer a stable luminescent signal, which has the potential to eliminate interference from the autofluorescence. A larger Stokes shift may also be anticipated due to the lower triplet energy level than that of the singlet. In addition, studying the generation of reactive oxygen species (ROS) is another aspect closely associated with phosphorescence.^24^ Thus, the presence of phosphorescence signals on AIEgens allows one to simultaneously take advantage of the turn-on nature, extended lifetime properties, and generation of ROS.

Herein, we aimed to use unsubstituted and benzene/thienyl-appended pyrrolo­[1,2-*a*]­quinoxalines (Figure 1). It was anticipated that conjugating benzene or thiophene ring via a single bond could affect the highest occupied molecular orbital (HOMO) and lowest unoccupied molecular orbital (LUMO) levels, as well as the postexcitation electron flow. Consequently, various photophysical properties, such as emission wavelength, photoluminescence quantum yield (PLQY), and radiative/nonradiative rates, can be significantly impacted. Further, the modification mentioned above could also influence the aggregation behavior and the occurrence of phosphorescence. This review presents our observations and analysis of the importance of the PQN scaffold as a versatile fluorophore. Our experimental studies are well supported by computational investigations on both singlet and triplet states. Features like fluorescence, phosphorescence, ISC, and possibilities of AIE and ROS generations are captured through the DFT- and TDDFT-based studies on the low-lying electronic states (ground and excited) of all PQN systems investigated in this work.

**Figure 1:**
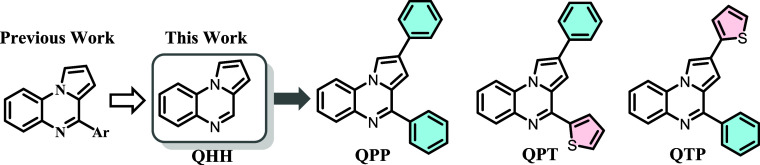
Pyrrolo­[1,2-*a*]­quinoxalines used in this study.

## Experimental Section

### Materials and Methods

Laboratory-grade solvents and reagents purchased from various commercial sources were used to synthesize the molecules. Analytical-grade solvents were used for the absorption and fluorescence experiments. Reactions were monitored by thin-layer chromatography (TLC) using precoated aluminum-supported silica gel plates (Merck 60 F254). UV light was used to observe the reactants and products (254 nm). Column chromatography was performed on silica gel (Merck 100–200 mesh). For the synthesized probes, ^1^H NMR and ^13^C NMR studies were conducted in CDCl_3_/DMSO-*d*_6_ solutions with tetramethylsilane (TMS) as the internal standard using a Bruker AV 400 spectrometer at 400 and 101 MHz, respectively. An Agilent LC-MS/MS Q-TOF 6540 was used to record the HRMS of the synthesized probes. A Jasco V-650 spectrophotometer and Horiba Fluorolog-3 were used to measure absorption and emission spectra, respectively. Fluorescence imaging of the cells was recorded through a Leica DMi8 confocal laser scanning microscope (CLSM) at 63× magnification. Fluorescence lifetime imaging (FLIM) experiments were carried out using a PicoQuant Microtime 200 time-resolved confocal microscope.

### Computational Details

Ground-state geometry optimizations (of monomers and dimers) have been performed in Gaussian 16 ^25^ at the DFT level, while their excited-state studies (optimizations and single point) are done at the TDDFT level of theory.^26^ In both cases, we have used the B3LYP functional^27,28^ and 6-311G** (6-31G* for trimers) basis sets. The SMD solvent model^29^ has been employed for the studies where dioxane and water have been used as the solvents. The spin–orbit coupling constants between the singlet and triplet states are derived at the TDDFT level of theory using PySOC program package.^30^ The computational studies of these PQNs are discussed below. The results support the experimental findings with proper justifications behind the observations, and based on these, we have proposed the mechanisms most likely involved in these photophysical processes.

## Results and Discussion

### Preliminary Photophysical Investigation

After the synthesis and characterization of the molecules (Figures S1–S12), solvatochromism/solvatofluorochromism experiments were carried out in a series of solvents, exhibiting diverse polarities and dielectric constant values spanning from 2.4 for toluene to 32.6 for methanol (Tables 1 and S1). The concentration of PQNs-10 μM was maintained throughout the study to ensure uniformity in the experimental conditions. The UV–vis spectra of all the compounds revealed minimal change in absorption profiles, indicating a lack of sensitivity toward polarity in the ground state (Figures S14–S21 and Table S2). In **QHH**, a broad absorption peak with a maxima at 340 nm was observed, which barely changed with polarity. However, its emission maxima shifted from 398 nm (in toluene) to 407 nm (in MeOH), demonstrating the effect of polarity in the excited state (Figure S22a). An improved Stokes shift (>70 nm) was noticed in the case of 2,4-disubstituted analogues, namely, **QPP**, **QPT**, and **QTP** (Figure S22b–d). These compounds showed prominent red-shifted absorption profiles from 350 to 362 nm due to increased conjugation and stronger donor–acceptor (D–A) interactions. In the case of **QPP**, the Stokes shift observed upon changing the polarity of the solvent ranged from 84 nm in toluene to 94 nm in methanol. Meanwhile, **QPT** and **QTP**, where thiophene and benzene rings were introduced, showed further improvement in the Stokes shift (for **QPT** toluene/MeOH, 97 nm/100 nm; for **QTP** toluene/MeOH, 94 nm/93 nm) (Table S1 and Figures S23–S26). The molar absorptivity coefficient was calculated for all of the molecules and found to be low for **QHH–QPT** when compared to **QPP** and **QTP**. The overall result indicated that the **QHH**, with its fused structure and lack of substituents, has a weak intramolecular charge transfer (ICT) compared to its substituted analogues **QPP**, **QTP**, and **QPT**.

**Table 1:** Absorption and Emission Wavelengths of PQNs in Solution and the Solid State^a^

	Toluene		Dioxane		THF		DCM		DMSO		MeOH		Solid
	**λ**_**abs**_	**λ**_**em**_	**λ**_**abs**_	**λ**_**em**_	**λ**_**abs**_	**λ**_**em**_	**λ**_**abs**_	**λ**_**em**_	**λ**_**abs**_	**λ**_**em**_	**λ**_**abs**_	**λ**_**em**_	**λ**_**em**_
QHH	340	398	338	402	337	401	337	402	338	408	337	407	430
QPP	356	440	355	430	354	404	354	448	355	404	354	408	453
QPT	362	459	360	458	348	431	364	458	366	466	360	460	464
QTP	357	451	355	431	358	405	355	443	361	454	357	450	467

a All the λ_abs_ (absorbance) and λ_em_ (emission) values are given in nm.

### Aggregation-Induced Emission Study

Our subsequent attempt explored the aggregation of compounds and its influence on luminescent properties. This study used dioxane and water as good and poor solvents, respectively. On increasing the percentage of water from 0 to 99%, changes in emission intensity, lifetime, and quantum yield were observed for **QHH**, **QPP**, **QPT**, and **QTP** at 424, 459, 468, and 481 nm, respectively (Figure 2). **QHH** displayed a marginal increase in the fluorescence intensity, which can be attributed to a restriction in the vibrational motion of the molecules. Red-shifted emission maxima were observed in **QPP** and **QTP** with approximately 6-fold enhancement. The origin of this behavior is possibly due to the aggregation of molecules and activation of the restriction in the intramolecular motion (RIM), which arises due to the introduction of rotors at the 2 and 4 positions. In contrast, **QPT** only showed a red shift in the fluorescence maxima without any change in intensity, which depicted the impact of delocalization and position of substituents in controlling the excited-state arrangement and molecular motion of the molecules in water.^31^ We used absorption experiments to analyze the nature of the aggregates using the dioxane:water mixture. All of the spectra showed ordered absorbance peaks in dioxane (100%). However, the increase in the water percentage resulted in broad spectra, with a concomitant decrease in the absorbance peak, possibly due to a change in packing (Figures S31–S34). Literature reports suggest the prevalence of J-type aggregates in polar medium. In fluorescence, a clear red-shifted emission of the molecules indicated an increase in rigidity and dipole–dipole interactions in polar media.

**Figure 2:**
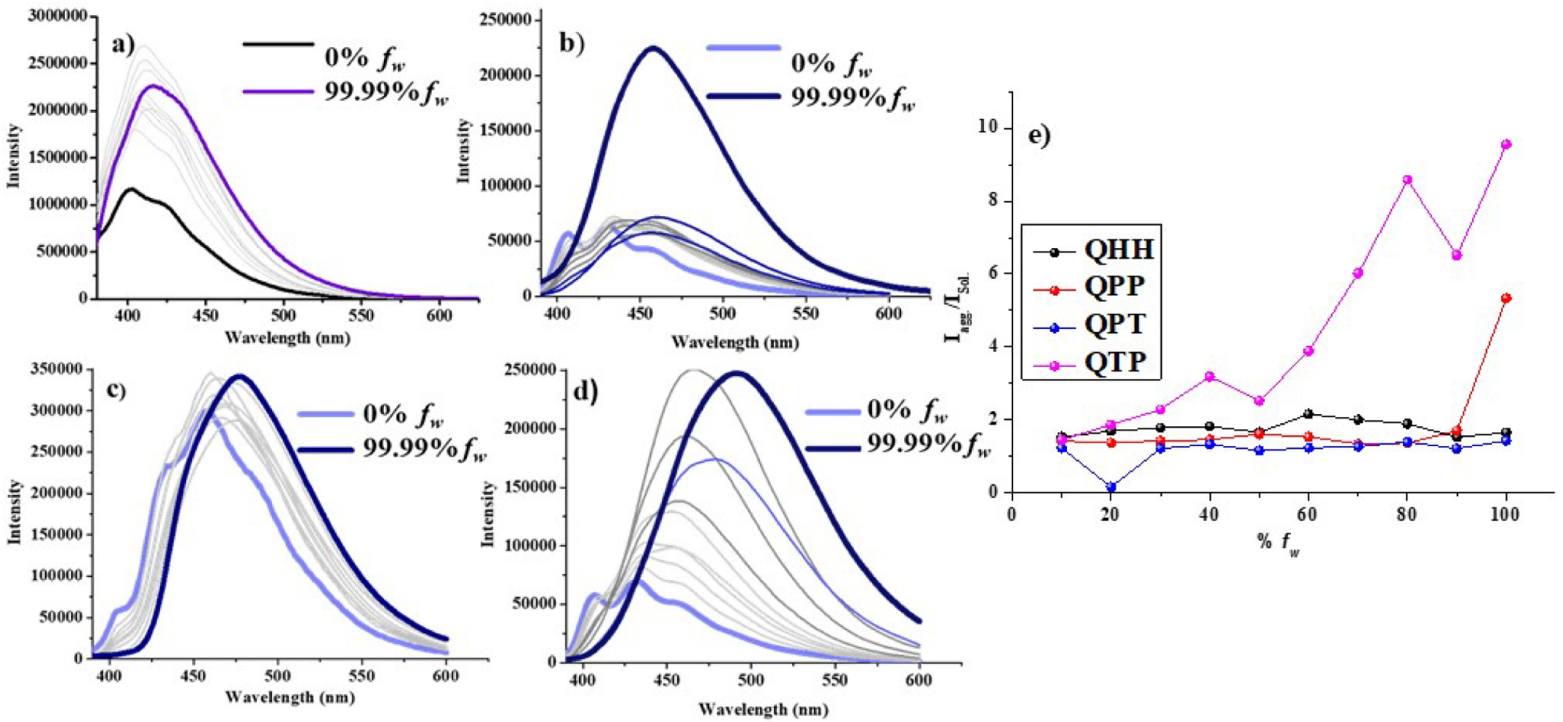
Fluorescence spectra of (a) **QHH**, (b) **QPP**, (c) **QPT**, and (d) **QTP** in different percentages of water and dioxane. (e) Relative fluorescence intensity graph of aggregates and solution state. (λ_ex_ 350 nm for **QHH** and λ_ex_ 360 nm for **QPP**, **QPT**, and **QTP**).

Preliminary observation of AIE was further substantiated by performing dynamic light scattering (DLS) and scanning electron microscopy (SEM) imaging to confirm the formation of supramolecular architectures (Figures S35 and S36). For **QHH**, the size of the aggregates was found to be ∼400 nm by DLS, and the SEM images indicated a change in morphology from spherical (in dioxane) to sheet-like structure (in water). With **QPP**, the size of the aggregates increased to ∼540 nm in DLS, and a change in morphology from nanoparticles (in dioxane) to nanowires (in water) was observed in SEM. Interestingly, **QPT** showed the formation of smaller particles of size ∼300 nm, and there was no change in morphology upon changing the medium from dioxane to water, except for a decrease in the size of the nanosheets. With **QTP**, the size of the aggregates increased to ∼950 nm; however, SEM imaging indicated that the morphology remained the same (nanosheet to microsheet) when the solvent was changed from dioxane to water.

### Lifetime Measurements

Upon completion of the steady-state fluorescence study, time-correlated single-photon counting (TCSPC) experiments were carried out to determine the lifetime of the molecules and parameters such as radiative decay (*K*_r_) and nonradiative decay (*K*_nr_) (Table S3 and Figures S27–S30). The lifetimes of all the molecules were recorded in 10 μM concentrations using a 375 nm laser excitation source. The average lifetime (τ_avg_) for various solvents with different polarities was recorded, and no sequential trends were observed (either low to high, or vice versa). The observed lifetimes (ns) in dioxane were low when compared to that in water (Figure S37). On *K*_r_ and *K*_nr_ calculation, it was observed that the nonradiative decay at 1.38 × 10^8^ s^–1^ for **QHH** was the lowest among all the molecules, which was expected due to the lack of substituents/molecular rotor at the second and fourth positions. On the other hand, K_nr_ values for **QPP**, **QPT**, and **QTP** were found to be 23.25 × 10^8^, 10.42 × 10^8^, and 3.88 × 10^8^ s^–1^, respectively, in dioxane. Thus, in dioxane as a solvent, the substituents at the second and fourth positions induce higher rotational motion, resulting in quanta loss via activation of the nonradiative channel. On the other hand, in water, we found an enhancement in the lifetime for all the molecules (except **QPT**). A detailed analysis of the lifetimes in dioxane, when compared to that in water, also indicated a greater tendency to form aggregates in **QHH**, **QPP**, and **QTP** as the values of τ_agg._/τ_sol._ follow the order **QHH** ≫ **QTP** > **QPP**> **QPT**. This observation was also supported by the values of K_nr_(sol.)/K_nr_(agg.).

### Solid-State Emission Study

AIE results prompted us to study the solid-state emission of the PQNs (Figures S38–S41). All of the compounds showed λ_em_ comparable to their aggregated state. Additionally, the quantum yield for the **QHH** molecule was found to be the lowest with the highest quantum yield for the **QTP** molecule. This behavior was substantially different from the quantum yields in the solution (Tables 1 and S4). The emission spectral overlap in all of the molecules in the solid and solution states also indicated similar packing/arrangement in the excited species. The PXRD spectra for PQNs were also recorded, which showed intense and sharp reflection peaks for **QHH**, indicative of its self-organized crystalline nature (Figure S47). On the other hand, **QPP**, **QPT**, and **QTP** showed broader and weaker reflection peaks, which point to the amorphous nature of these molecules. TCSPC studies performed on the molecules gave the lifetime of the molecules in the 1.2–1.9 ns range (Figures S42–45).

### Analysis of the QTP Crystal Structure

After completion of the solid-state emission studies, crystal analysis of only **QTP** could be carried out, as other molecules failed to crystallize. **QTP** was obtained, possessing a rigid π-system with two appended rotors (thiophene and benzene ring). The calculated angles between the PQN core and the rotors, thiophene, and benzene ring are 25.9° and 118.78°, respectively. It seems that the small dihedral angle between the PQN core and thiophene may induce easy electron transfer on excitation, thereby ushering in the aggregation-induced emission enhancement (AIEE) phenomenon in **QTP**.^32^ The thiophene (Figure 3a,b) ring also plays a critical role in **QTP** crystal packing. C–H of the thiophene ring forms weak intermolecular interactions with neighboring molecules, which probably stabilizes the crystal. On the other hand, the pyridine ring present in the PQN core forms weak intermolecular hydrogen bonding (C–H··· . N; 2.862 Å) with the *peri* hydrogen of the adjacent PQN ring and promotes crystal growth in the XY-plane (Figure 3c). In addition, the intermolecular vertical spacing between parallel molecules is restricted between 3.21 and 3.45 Å, indicating π–π interaction (Figure 3d). Further, Hirshfeld surface analysis was used to quantify the weak intermolecular interactions (Figure 3e–j). It showed that H---H forms the most significant interaction (49%) between **QTP** molecules, followed by C---N (18.5%) and C---H (15.4%) interactions (Figure S46).

**Figure 3:**
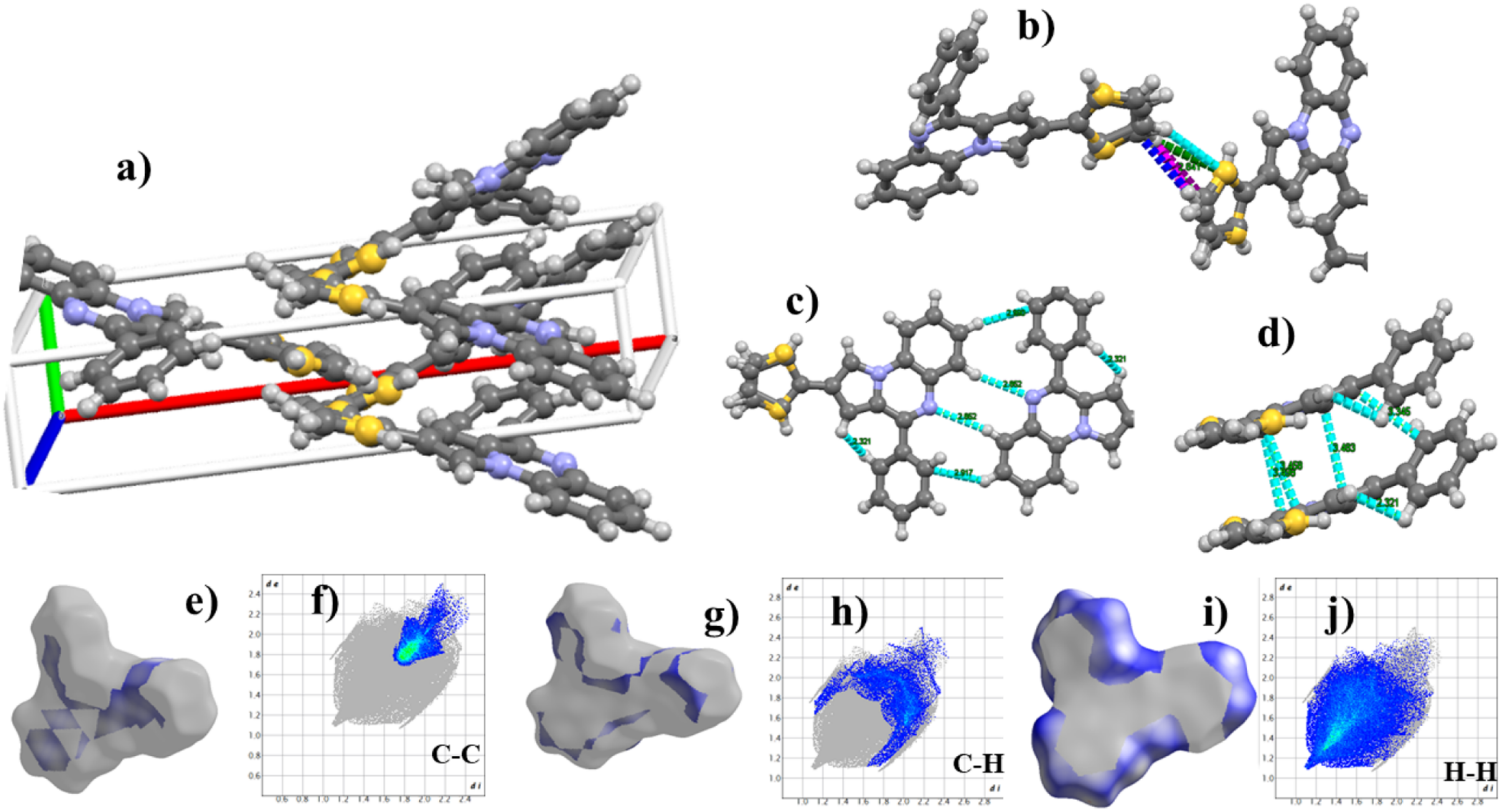
(a) Crystal packing of the **QTP** crystal in the unit cell, (b) S–H bond interaction, (c) peri-hydrogen interaction in the XY-plane, (d) vertical π–π interaction and corresponding distances, and (e–j) Hirshfeld surface for weak C–C, C–H, and H–H interaction and respective quantification.

Preliminary pH screening of the molecules showed a bathochromic shift for the emission signal, with a maximum 97 nm red shift in the case of **QTP**. However, the molecules retained their emission behavior in alkaline pH. The acid sensitivity of the PQNs was further used for suborganelle imaging.

### Cell Imaging

The MTT assay was initially carried out, which showed the nontoxic nature of PQNs (Figures S53-54). Preliminary experiments with RAW 264.7 cells revealed the cell-permeable nature of the probes (Figure S55). Experiments were also conducted in CAL-27 cells and different organelle-targeting dyes (Figures S55b,a and S56–S59). There was no localization of the probes in the nucleus, which was validated using DRAQ (the nucleus-specific probe) (Figure S55b). Experiments, when carried out in the presence of LysoTracker (lysosome-specific dye)/Nile red (lipid droplet-specific dye)/MitoRed (mitochondria-specific dye), revealed minimal specificity toward any organelle by QHH (Figures 4a and S56). However, **QPP** and **QTP** displayed strong affinity toward the lysosomal compartment of the cell, with Pearson coefficients of 0.819 and 0.849, respectively (Figures 4c, S57, and S59). **QPT**, on the other hand, showed a preference for the lipid droplets in the CAL-27 cells (Pearson coefficient = 0.829; Figures 4c and S58).

**Figure 4:**
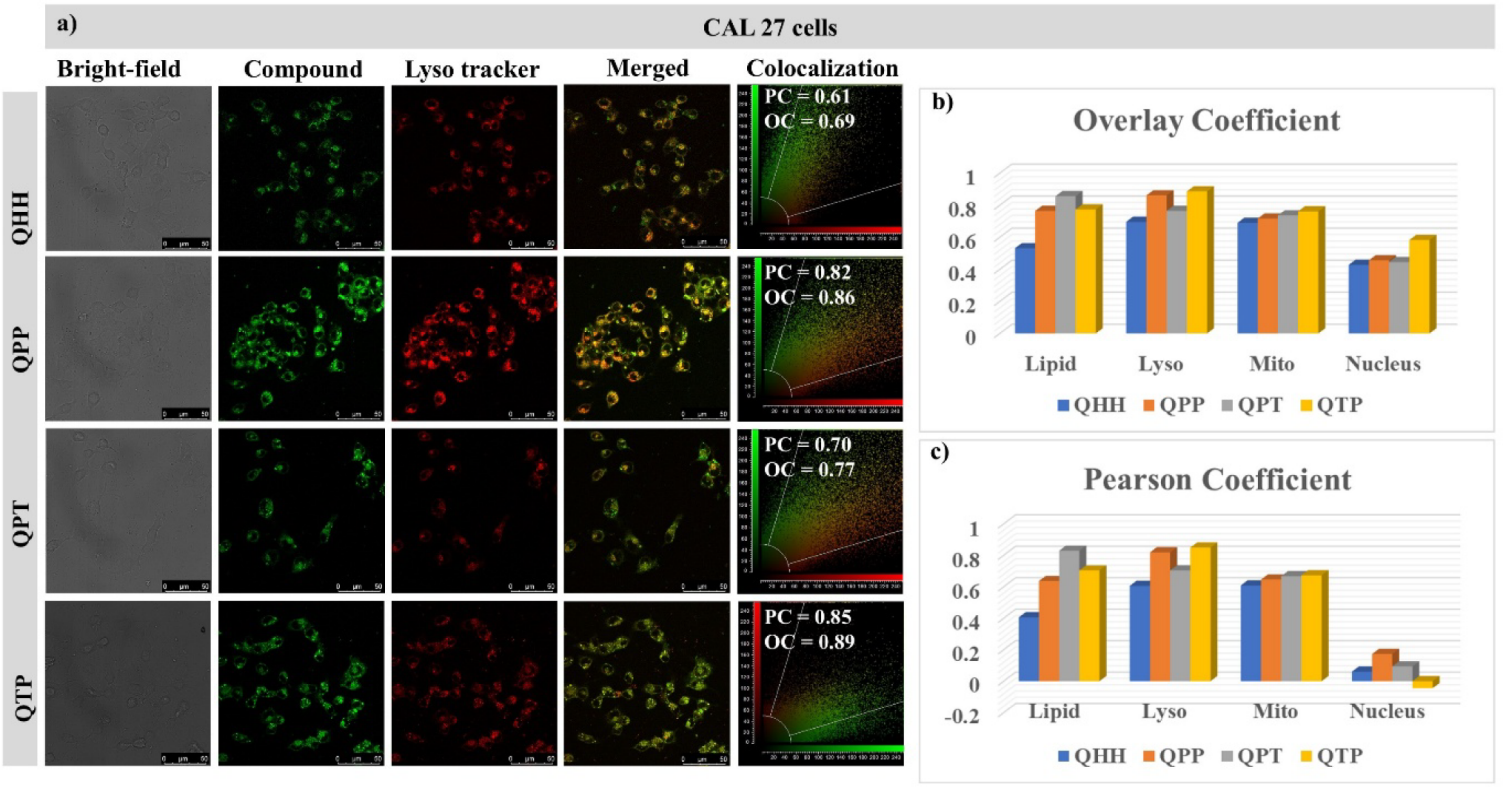
(a) CLSM imaging of PQNs (10 μM, λ_ex_= 405 nm, λ_em_= 415–550 nm) in CAL 27 cells with LysoTracker Red (100 nM, λ_ex_ = 514 nm, λ_em_ = 550–600 nm) and the corresponding (b) overlay coefficient and (c) Pearson coefficient data of PQNs with different organelles. Scale bar = 50 μm.

After completing the preliminary confocal experiments, the effect of polarity changes was monitored in RAW cells using the fluorescence lifetime imaging microscopy (FLIM) technique (Figure S60). Molecules chosen for this purpose were **QHH** and **QTP**, based on their TCSPC results, where significant changes were noticed in the average lifetimes upon changing the polarity and pH. FLIM experiments showed the sensitivity of the probes to the changes in the cellular microenvironment, especially with respect to the changes in polarity and pH (Figures S48–S52).

### Computational Studies

#### Monomer-Optimized Geometries and Their Properties

##### Absorption and Fluorescence Peaks (Table S5a,b)

The absorption peaks predicted based on the vertical excitations involving the low-lying electronic transitions at the optimized ground-state geometries in dioxane and water match the observed UV–vis peaks very closely. In **QPP**, **QPT**, and **QTP**, both S_0_–S_1_ and S_0_–S_2_ are strongly allowed transitions arising from the H→L and H-1→L excitations (HOMO = H, LUMO = L), respectively, while in **QHH**, S_0_–S_1_ is the only strongly allowed transition (H→L). It must be added that in both solvents, **QTP** shows a stronger S_0_–S_2_ transition than S_0_–S_1_. Further studies on the S_2_ state reveal that in all three systems (**QPP**, **QPT**, and **QTP**), during the relaxation from the vertically excited state, it becomes degenerate (very close to the Franck–Condon geometry) with the S_1_ state (and with a higher triplet state). The S_0_–S_2_ transition, being very weak in **QHH**, is absent in this system. The fluorescence peak positions predicted based on the S_1_–S_0_ energy gap at the S_1_ minima of all these systems are in line with their experimentally observed values. Only in the case of **QTP**, two S_1_ minima (here mentioned as S_1_ and S_1_′) are obtained. The one designated as S_1_′ has a different geometry than S_1_ (discussed in the next section). It must be added that the fluorescence peak corresponding to the S_1_–S_0_ emission in **QTP** is significantly red-shifted compared to its S_1_′–S_0_ emission. The observed lower oscillator strength values of the S_1_–S_0_ transitions in dioxane (f ≈ 0.25) with respect to that in water (f ≈ 0.46) are in line with the lower φ_f_ values and the intensities observed in the former (for **QHH**, **QPP**, and **QTP**). These theoretical results have correctly captured the red shifts of the fluorescence peaks with a change of solvent from dioxane to water.

##### Geometries

A comparative study of the optimized geometries (Figures S61 and S62) of the ground state (S_0_) and the first excited state (S_1_) in water reveals that the C–C bond connecting the PQN unit (pyrrole side) and the benzene ring remains unchanged in these two geometries for **QPP** and **QPT**. In **QTP**, where this unit is connected to the thiophene ring, the C–C bond length remains unaltered in S_1_ (Figure S61) while it decreases drastically in S_1_′ from 1.46 to 1.40 Å (Figure S63). The mentioned benzene rings (in **QPP**, **QPT**) do not deviate from the plane of the PQN ring in the S_1_ geometries compared to that of the S_0_ geometries. In **QTP**, this deviation of the benzene ring is by 7° (<CCCC ≈ −9° to −16°) in the S_1_ minimum, while in S_1_′, this ring becomes completely planar. It must be added that in the previously discussed geometry, where the S_2_ state during relaxation becomes nearly degenerate with the S_1_ state (and a triplet state), the above-mentioned C–C bond decreases by 0.04 Å and the benzene or thiophene ring becomes more planar through a torsion along the <CCCC dihedral angle. On the other side of the PQN unit, where the pyrazine moiety is connected to the benzene (in **QPP** and **QTP**) or the thiophene ring (in **QPT**), a decrease in the connecting C–C bond lengths can be seen from S_0_ to S_1_. These benzene rings in **QPP** and **QTP** become more planar (<CCCC changes from 40° to 15°) with respect to the PQN moiety in the S_1_ (or S_1_′) state; the change in this angle is more pronounced in **QPT** (<CCCC changes from 41° to 6°).

##### Charge Distributions

The relatively more fluorescent **QHH** molecule exhibits a rigid planar structure with delocalized electron density across the π-conjugated backbone (Figure S61). The electronic charge clouds in the HOMO and LUMO (S_0_–S_1_ dominated by H→L excitation) are found to be absent on the pyrrole-side (of PQN)-connected benzene rings in the S_1_ states of **QPP**, **QPT**, and **QTP**. A transfer of charge from the PQN part to the pyridine-connected benzene or thiophene rings can be seen in these excited states, particularly in **QPP**. A more drastic transfer of charge can be seen in this part in the vertically excited S_2_ states (S_0_–S_2_ dominated by H-1→L excitation). In these cases, the HOMO–1 charge cloud is on the PQN unit and on its pyrrole-adjacent benzene or thiophene rings, while in the LUMO, it is on the other side. In the **QTP** S_1_′ geometry, the nature of HOMO and HOMO–1 is exactly the reverse in comparison to that of the S_1_ geometry. Here, the fused benzene ring of the PQN unit in HOMO lacks any charge cloud like the other benzene ring, and a clear charge transfer from the right to the left during the H→L excitation (similar to the H-1→L in S_2_) has occurred as we move from the ground to this excited state.

### Dimer (π-Stacked) Optimized Geometries and Their Properties

In the next stage of our work, we studied the possibilities of the π-stacked dimer forms (excited and ground-state minima) for all four systems in water. Subsequently, predictions of their corresponding emission and absorption peak positions were made . These values are checked with the monomer-predicted and experimental values.

(A) Optimizations of the lowest excited singlet state geometries have led to two distinct varieties of S_1_ π-stacked dimer minima: (A) The **QHH** and **QPT** molecules show exactly head-to-head (H-like aggregate) stacking where the two monomer units are situated around 3.2–3.7 Å apart (Figure 5). The slip angles are nearly 90° and structurally resemble the H-aggregate nature. However, these lowest singlet excited states of the dimers are not the fluorescent states, as shown by the almost zero oscillator strength values of the lowest S_1_–S_0_ emission at these S_1_ minima geometries. In fact, the emissive bright state here is a high-lying singlet state (S_4_) as shown by the high oscillator strength value of the S_4_–S_0_ transition from the TDDFT results. However, such high-lying states (S_4_) will undergo internal conversions or the surface crossings (or avoided crossings) with the low-lying dark singlet excited states, and consequently, the fluorescence strength is likely to reduce or disappear. It must be mentioned that this is a common feature of the H-aggregates where the fluorescence is normally quenched due to the low-lying dark states. The ground state (S_0_)-optimized dimer geometries of these two systems are similar to their respective S_1_ minima with the monomer units situated at a slightly higher distance (4.0–4.1 Å), and the slip angle values are marginally lower than 90° (75° and 77°). In each case, the TDDFT level of study shows that S_4_ is the bright state of these dimers with the absorption peak positions giving comparable results to the experimental ones.

**Figure 5:**
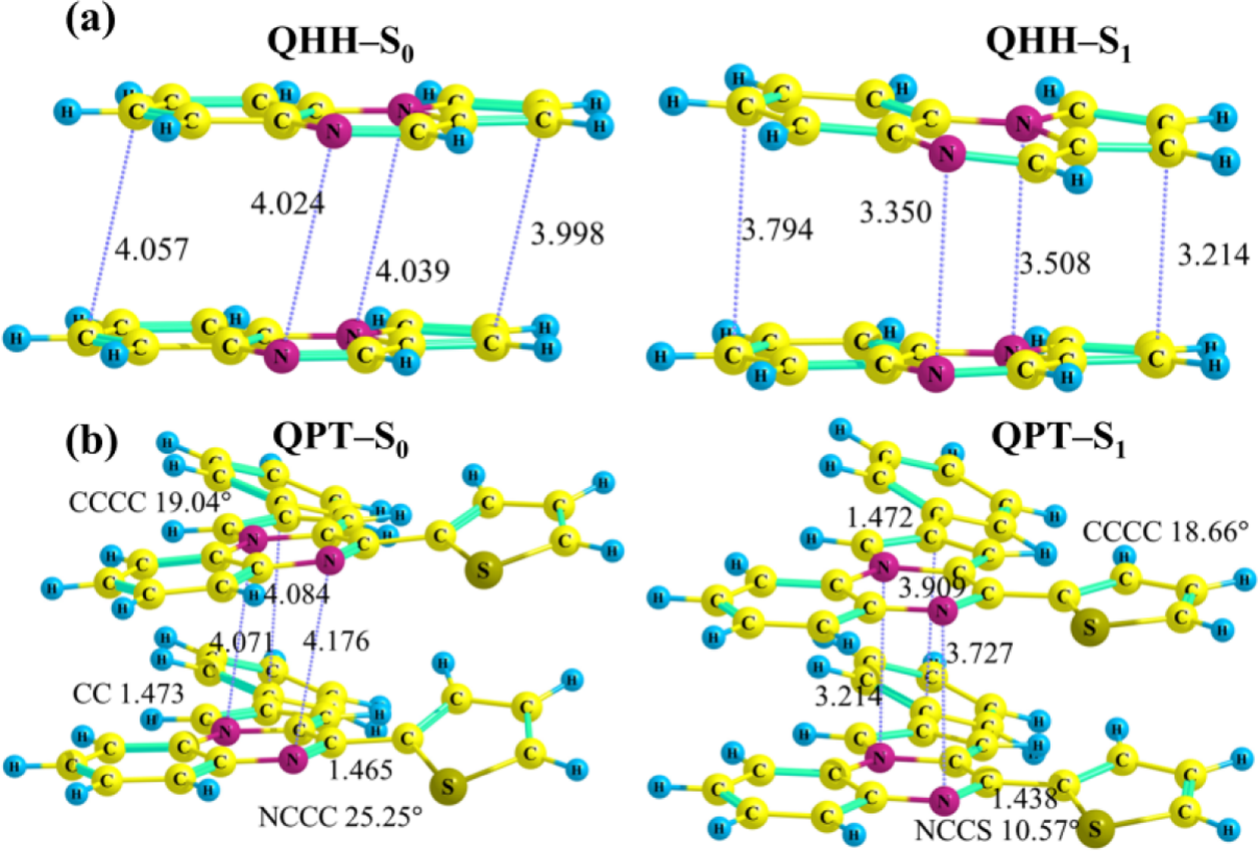
Dimer (π-stacked) optimized S_0_ and S_1_ geometries of (a) **QHH** and (b) **QPT.**

(B) On the other hand, the structures of the optimized S_1_ of π-stacked dimers of **QPP** and **QTP** (Figure S64) are not similar to the above-mentioned ones. Here, we found two different types of optimized geometries of the dimers in each case. Due to the stacking of the S_1_ and S_1_′ monomer geometries individually, we get two different dimers for **QTP**. These structures seem to have a slip-stacked head-to-tail arrangement (J-like aggregate) with slip angle values close to 50° and look distinctly different from those of **QHH** and **QPT**. On the other hand, in **QPP**, the two optimized dimers look different from **QTP** and others. Unlike in **QHH** and **QPT**, in both **QTP** and **QPP** π-stacked dimers, the lowest singlet states are the bright states, as indicated by strong oscillator strengths of the S_1_–S_0_ transitions (f ≈ 0.43), and the energy gaps are in good agreement with the observed emission peak (and the theoretically derived monomer peak) positions. The two S_1_-monomer units in these dimers in both cases are 4.5–5.0 Å apart from each other, indicating comparatively less interactions than **QHH** and **QPT**. Moreover, in their optimized ground-state (S_0_) geometries, the monomer units are more distant from each other, and looking at their geometries, it seems that no (or less) interaction is there between the two units.

Overall, studies on these optimized dimer geometries reveal head-to-head π-stacked arrangements for **QHH** and **QPT**, which resemble an H-type aggregate nature. In these cases, the fluorescence is likely to be quenched as the lowest state (S_1_) is dark, and the high-lying bright singlet state will undergo crossing or internal conversions with the low-lying states. On the other hand, in **QPP** and **QTP**, π-stacked dimer formation in the ground state will lead to less interaction between the monomer units, which is likely to increase in the excited state and have a different look from that of **QHH** and **QPT**. The **QTP** dimer shows a head-to-tail slip-stacked arrangement and has features like a J-stack aggregate; however, the **QPP** dimer looks different. In both cases (**QPP** and **QTP**), the bright states are the lowest singlet states, and the oscillator strength values of their S_1_–S_0_ transitions are similar to those of the monomers.

### Trimers

Though the above-mentioned studies on the dimers have been performed on relaxed geometries in water without any constraints on the twisting or other motions, the results can help us to understand the tendency or the nature of the stacking of the monomer units during the aggregate formations in solution. In the aggregated state, it is expected that the monomers in the solution will stack without any further relaxation (such as twisting of the dihedral angles) in the geometry. Considering this, we have explored emission possibilities from the parallel-stacked dimer and trimer aggregates of the S_1_ monomer geometries in water. These single-point TDDFT calculations have been performed on the **QTP** molecule (Figure S65), where the enhancement in fluorescence observed (in the possible aggregated state) in water is significantly high. The **QTP** S_1_-monomers are kept (4 Å apart) in a slip-stacked arrangement with a nearly 50° value of the slip angle. In line with Kasha’s exciton coupling model,^33–35^ the S_1_–S_0_ oscillator strength for the dimer is found to be almost two times the monomer, which becomes nearly thrice in the case of the trimer. In each case, the predicted emission positions are similar to those of the optimized relaxed dimer state discussed previously, and the observed significant red shift from the dioxane emission peak is also maintained.

### ISC, Phosphorescence, and ROS Generation Possibilities

#### ISC Between S_2_ and T_3_/T_4_

As discussed previously, the vertically excited S_2_ state (in **QPP**, **QPT**, **QTP**) relaxes through a comparatively more planar geometry (Figure S66) where the two excited singlet states (S_1_ and S_2_) are degenerate, which finally leads to the fluorescent S_1_ minima. An interesting observation at this geometry is the degeneracy of a higher triplet state (T_3_ in QPT, T_4_ in **QTP**) with these singlet states, indicating a possible ISC channel operating here. In **QPP**, the T_4_ state lies slightly above the singlets. Such degeneracy of the T_1_ state at the singlet–singlet (S_0_/S_1_) crossing geometry has been reported in the literature.^36^ However, these high-lying triplets are not found to have any relaxed minima as they undergo crossing (in **QPT**) or avoided crossing (in **QTP**) with their lower triplet states, indicating population transfer to these latter states.

#### ISC between S_1_ and T_2_/T_3_

The lower triplet states (T_2_, T_1_ in **QHH**, **QPP**, and **QPT**, and T_3_, T_1_ in **QTP**) were found to have relaxed minima in water. The Δ*E*_ST_ values based on the energy gaps of the optimized minima geometries of singlet (S_1_) and triplets (S_1_–T_2_/T_3_) are only 0.1 eV in **QHH**, **QPP**, and **QTP**, while for **QPT**, it is 0.6 eV. The vertical energy gaps at the S_1_ minima (also S_1_′ for **QTP**) geometries with the triplets (T_1_, T_2_, T_3_) at these geometries are also checked. This shows that the energy of the T_2_ states is the nearest (0 to 0.25 eV) to the S_1_ minima (vertically), while for the S_1_′ of **QTP**, the energy gap is lowest (0.15 eV) for the T_3_ state. These results clearly indicate the S_1_–T_2_/T_3_ intersystem crossing. Normally, a triplet–singlet energy gap below 0.3 eV is known to be good enough to have an ISC process.^37,38^ The spin–orbit coupling plays an important role in populating the triplet states as well. We have analyzed these coupling parameters, and the values (Table S6) suggest moderately strong coupling between these singlets and triplets. In all the cases, the lowest triplet state (T_1_) minimum is situated far below the singlet minimum (0.7 to 1.0 eV). The vertical energy gap of T_2_(min)–T_1_ in **QPP** is 0.24 eV, while for **QPT** and **QHH**, the values are roughly 0.42–0.54 eV. The results indicate chances of better T_2_–T_1_ internal conversion in **QPP**.

#### Phosphorescence Possibilities

The energy difference between the optimized triplet minima (T_2_ for **QHH**, **QPP**, **QPT**, and T_3_ for **QTP**) and the S_0_ state at these geometries corresponds to 2.50 eV (∼500 nm) to 2.75 eV (∼450 nm) values. The expected emission peaks based on these energy gaps are 445 nm in **QHH**, 493 nm in **QPP**, 505 nm in **QPT**, and 476 nm in **QTP**. These results indicate the possibilities of phosphorescence emissions (T_2_/T_3_–S_0_) of these systems (close to fluorescence emissions) following the anti-Kasha rule. The T_1_(minimum)–S_0_ energy gaps are much lower (1.45–1.55 eV) in these systems, except in **QHH**, where this gap is slightly higher (1.75 eV). These lowest triplet states will gain population from the T_2_ states through internal conversion, as discussed above. A schematic picture of the phosphorescence emissions can be seen in Figure 6.

**Figure 6:**
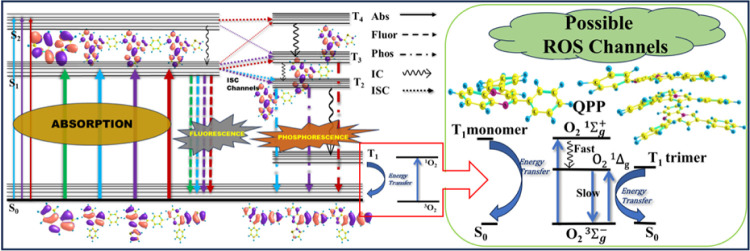
Schematic diagram of absorption, fluorescence, ISC, phosphorescence, and possible ROS channels from T_1_ (monomer and trimer) of **QPP**.

#### Structure, Charge, and Dipole Moment Change from Singlet (S_1_) to Triplet (T_2_/T_3_)

In the **QPP**, **QPT**, and **QTP** molecules, as we move from the excited singlet (S_1_) minima to the triplet (T_2_) minima, the benzene ring adjacent to the fused-pyrrole becomes planar through a rotation of the <CCCC dihedral angle by almost 10–20°. The length of the connecting C–C bond decreases by 0.03–0.07 Å. Unlike in the S_1_ state, the π-cloud on the mentioned benzene rings can be seen in these triplet states for all three systems (Figure S66). Analysis of the HOMO and LUMO indicates that in **QPP**, **QPT**, and **QTP** molecules, the triplet states accessed through ISC from the S_1_ state have a better donor (D)–acceptor (A) property. The higher dipole moment values of these triplet states (**QPP** T_2_: 8.4 D, **QPT** T_2_: 14.8 D, **QTP** T_3_: 12.9 D) in comparison to their S_1_ states (**QPP**: 7.3 D, QPT: 7.4 D, **QTP**: 6.7 D) clearly show this increase in charge separation accompanying the ISC process. It is well known that a change in the torsion angle in twisted DA molecules can play a major role in the ISC process, as a significant twist accompanies a large change in orbital angular momentum, which leads to a strong SOC effect.^39^ Among the four studied systems, **QPP** undergoes the maximum change in the torsion angle where both of the benzene rings connected to the QPN-unit change by nearly 150 during the singlet–triplet conversion, while the planar **QHH**, being fully rigid, does not correspond to any twist. The dipole moment of the latter decreases from S_1_ (6.2 D) to T_2_ (1.9 D), indicating a decrease in charge separation during this conversion, which may lead to comparatively less ISC in this system. The high fluorescence intensity of **QHH** in the solvents justifies the possibility of less ISC (S_1_–T_2_) in this system. Additionally, the lack of population of its S_2_ state and the absence of subsequent ISC to the higher triplet states (mentioned in the section titled ISC between S_2_ and T_3_/T_4_) may also have a negative influence on its phosphorescence. It must be added that the charge distribution in S_1_′ of **QTP** is similar to that of T_3_, where the thiophene ring attached to the PQN unit is planar in both of these states. All related energy values, coupling constants, and dipole moments can be found in the Supporting Information.

#### Energy Gap of Triplets and Chances of ROS Generation

We have tried to investigate the chances of a T_1_ state population from the higher triplet states (probable phosphorescent states here), as these may provide information about the possible ROS generation ability of this low-lying triplet state. One can expect better ROS generation for the systems where better ISC and, subsequently, higher phosphorescent states or well-populated triplet states are found. The energies of the higher triplet phosphorescent states populated through ISC (discussed earlier) hold the key as their low energy gaps with the T_1_ states or crossing with the lower triplets (like T_3_/T_2_ followed by T_2_/T_1_ in **QTP**) can indicate better transfer of population to this lowest triplet state through IC. Subsequently, this populated T_1_ state can be involved in generating the ROS. Among the studied molecules, **QPT** shows the highest energy gap (T_2_–T_1_ vertical) at the T_2_ minimum (0.54 eV or 12 kcal/mol), followed by **QHH** (0.42 eV or 10 kcal/mol). This energy gap in **QPP** is the lowest (0.24 eV or 5.5 kcal/mol), indicating better chances of internal conversion to the T_1_ state. In QTP, at the T_3_ minimum, the energy gap (T_3_–T_2_) is 0.20 eV; here, the T_2_ state has no minimum, and crossing with the T_1_ state has been observed. The energy gap (Table S7) value between the T_2_ minimum and T_1_ minimum is highest for **QHH** (1.14 eV), while for **QPP**, it is 0.8 eV. Overall, **QHH** is expected to have the least populated T_1_ state and, hence, the lowest chances of ROS generation. The previously discussed lower ISC (to T_2_) in this planar rigid molecule makes the chances of population of T_1_ further lower. It becomes apparent from these monomer studies that the other three PQNs may have possibilities to generate ROS. It must be added that in recent times, the aggregated induced ISC (AIISC) process has been investigated by several groups.^37–40^ These studies have indicated that in the aggregated form, a better ISC process can occur due to the formation of band-like states. Similar possibilities cannot be ruled out here also, though, as discussed, in our systems, the small energy gaps between the S_1_ and T_2_ (or T_3_ in **QTP**) states in the monomer indicate that without aggregation, the ISC process can operate here, as well. Though aggregates will certainly help (we have checked with the dimer aggregates on these triplet states) in reducing the energy gap between the T_2_ (or higher triplet) and T_1_ states, the latter can be more effective in transferring energy to the triplet oxygen. Now, coming back to the point of ROS generation (Figure 6), we have seen that the calculated T_1_–S_0_ energy gaps for the **QPP** and **QTP** systems are nearly 1.55 eV, while for **QPT**, this value is 1.45 eV. The values of the first two systems, in particular, are very close to the X^3^Σ_g_^–^–b^1^Σ_g_^+^ energy gap of O_2_ (1.60 eV) reported in the literature. A triplet energy transfer can populate the higher singlet state (b^1^Σ_g_^+^) of the crystalline aqueous phase of O_2_. This chemically unreactive state, being unstable, is known to convert (within picoseconds) to the lower singlet state (a^1^Δ_g_), particularly in aqueous solution.^41,42^ The latter state (lifetime in microseconds) is chemically reactive and may undergo reactive oxygen generation. To check the possibility of ROS generation in the aggregated state, we have taken the π-stacked trimer of **QPP** and calculated the energy difference between the T_1_ and S_0_ states. This energy gap is found to be only 0.94 eV (in the monomer, it was 1.55 eV) and almost matches the reported X^3^Σ_g_^–^ – a^1^Δ_g_ energy gap of O_2_ (0.95 eV), which produces the more reactive state (a^1^Δ_g_). Therefore, the aggregation-induced ROS generation through the decrease in the T_1_–S_0_ energy gap seems to be a possibility here. However, proper experimental analyses like phosphorescence and ROS studies are required to be performed on these PQNs to check the validity of these predictions, which were made through computational studies on the triplet states.

### Phosphorescence

Based on the observations from the computational studies, we decided to record the phosphorescence spectra of the molecules (Figure S67). Phosphorescence spectra were recorded at 370–700 nm and 90 K for all four molecules, upon excitation at 340 nm for **QHH** and at 360 nm for **QPP**, **QPT**, and **QTP**, respectively. The phosphorescence intensity was the lowest for **QHH** (λ_phos_ = 414 nm), which was very close to the fluorescence emission wavelength (λ_em_ = 398 nm), and the highest for **QPP** (λ_phos_ = 484 nm). However, **QPT** showed two peaks of similar intensity, blue-shifted (λ_phos_= 409 nm), and red-shifted (λ_phos_ = 472 nm), whereas λ_em_ was 450 nm. **QTP** has shown a similar phosphorescence intensity with a single red-shifted peak (λ_phos_ = 477 nm) from fluorescence spectra (λ_em_ = 448 nm). The close fluorescence and phosphorescence in all cases suggest the position of the emissive triplet state near the emissive singlet states. The observed phosphorescence results align with the computational studies reported on triplet states. In fact, the predicted probable absence or weakest peak of **QHH** is also found to be correct.

### Studies on ROS Generation

The role played by aggregation in strengthening the singlet–triplet energy matches (Δ*E*_ST_) via enhancement of the ISC rate and prolonging of triplet state lifetime is well established. Therefore, considering the aggregation of the PQNs, a separate study was initiated to explore these molecules for the reactive oxygen species (ROS) generation.^37^ Herein, CAL-27 cells were incubated with PQNs under white light irradiation. The irradiated cells were further treated with dichlorodihydrofluorescein diacetate (DCFDA) under dark conditions. Cells were observed in two channels for PQNs (green pseudo-color, λ_ex_ 405 nm) and DCFDA (red pseudo-color, λ_ex_ 488 nm). As evident from Figure 7a, visualization of DCFDA fluorescence indicated the generation of ROS. Among all of the PQNs, the highest fluorescence was observed for **QPP** (Figure 7b). Based on the brightest fluorescence output of DCFDA in the presence of **QPP**, we performed another experiment to visualize singlet oxygen generation in a cuvette with **QPP** and DCFDA. The solution was initially kept under dark conditions with occasional white light irradiation to trigger ROS generation. It showed a gradual increase in the fluorescence intensity of the dichloro-dihydro-fluorescein (DCFH) solution at 522 nm (λ_ex_ 488 nm) (Figure 7c).

**Figure 7:**
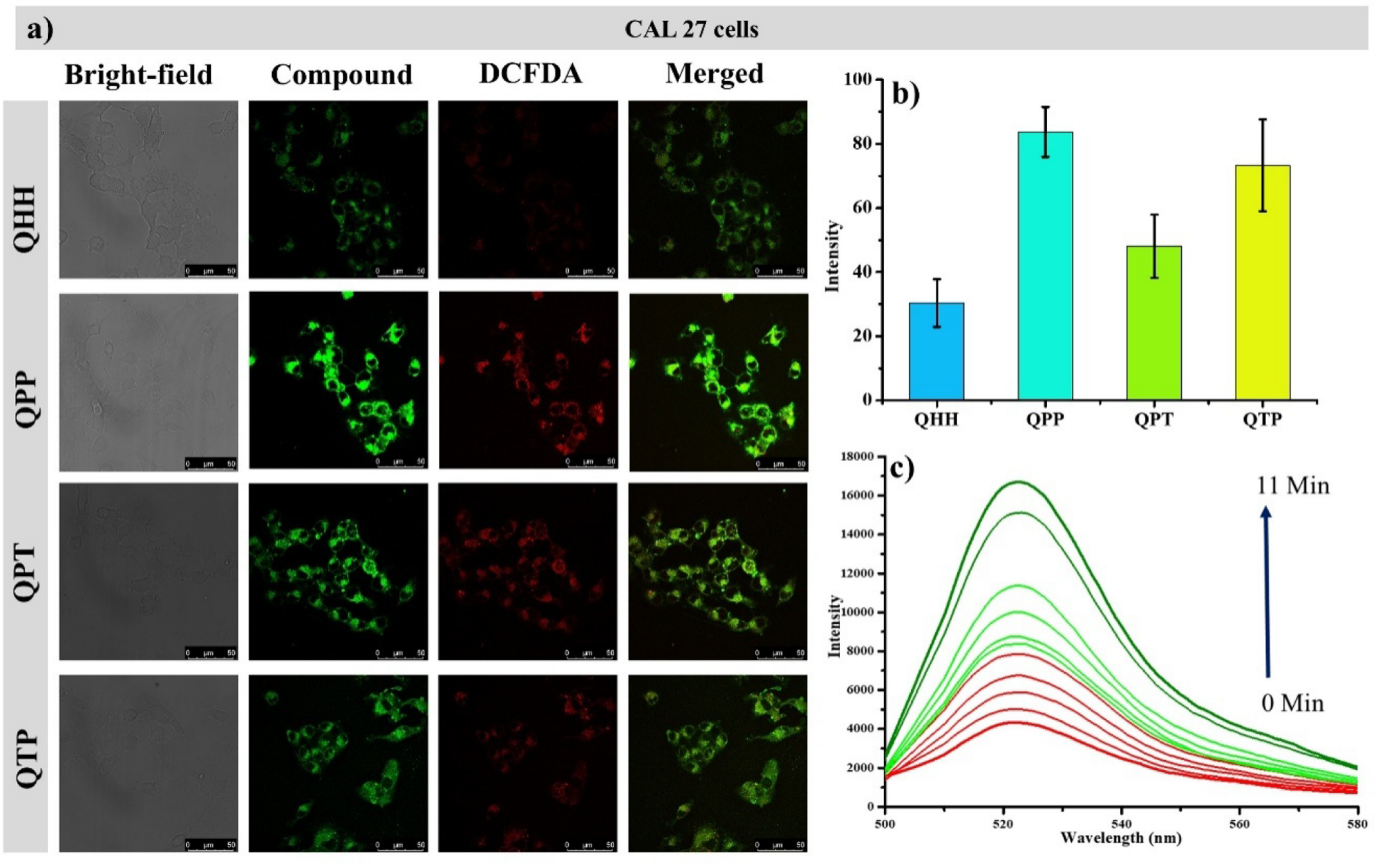
DCFDA assay of PQNs and CLSM imaging. (a) Quantified fluorescence intensity of DCFDA with PQNs. (b) Treatment of **QPP** with DCFDA and consequent generation of DCFH.

Further studies to confirm ROS generation were performed with 1,3-dibenzeneisobenzofuran (DPBF) dye. Herein, the probe molecules in dioxane were treated with DPBF, and their absorbance spectra were recorded (Figures 8 and S68–S74). A decrease in the absorbance peak at 414 nm was noticed in all of the experiments. However, it was most rapid for **QPP** and **QTP**, whereas it was relatively sluggish for **QHH** and **QPT**. Experiments were also performed with PQN, DPBF, and NaN_3_ (a known singlet oxygen quencher) to further establish the generation of singlet oxygen species (Figures 8, S70, and S74). These experiments demonstrated a drastic reduction in the absorbance change. A control experiment with only DPBF in dioxane showed slower dye photodegradation.

**Figure 8:**
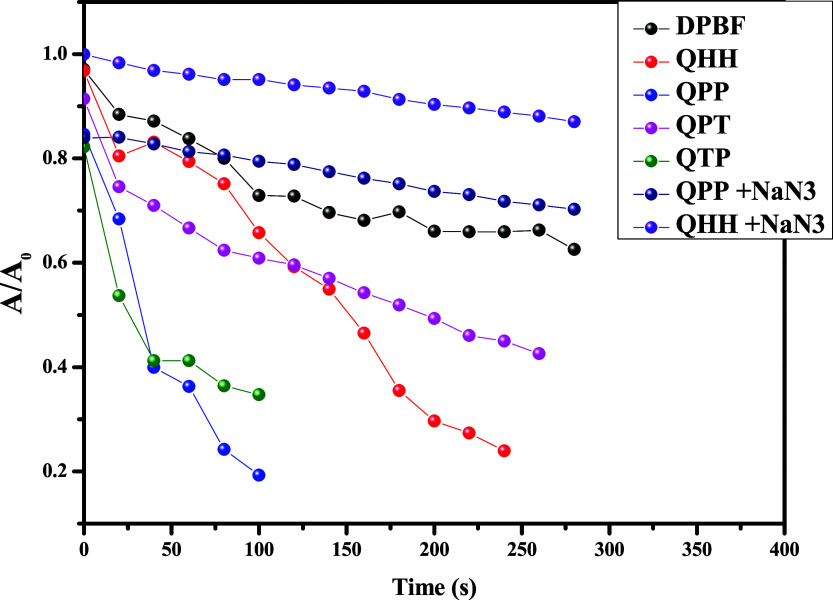
Relative absorption change of DPBF with/without NaN_3_ in the presence of PQNs after white light irradiation.

The ROS generation ability displayed by **QPP** and **QTP** aligns with the computational studies, where the calculated T_1_–S_0_ energy gap of the monomeric system was found to be close to the X^3^Σ_g_^–^–b^1^Σ_g_^+^ energy gap of O_2_. As discussed, further studies of the π-stacked trimer of **QPP** revealed that this gap reduces upon aggregation and becomes exactly the same as the energy required to populate the ROS state (b^1^Δ_g_) of O_2_. This indicates that the aggregation here is likely to promote the generation of the ROS state of O_2_, a feature that has been highlighted in several AIEgen-induced reactive oxygen generation studies in recent times.^43^

## Conclusion

In conclusion, this work has shown that the presence of substituents at the 2/4 positions of pyrrolo­[1,2-*a*]­quinoxalines (PQNs) leads to pronounced differences in their ICT characteristics, solvatochromism, and AIEE properties. Solid-state studies confirmed AIE behavior with QTP showing the highest quantum yield and π–π interactions in crystal packing. Cell imaging revealed lysosomal localization for QPP and QTP, while the QPT targeted lipid droplets. Theoretical calculations aligned well with experimental results, capturing solvent-dependent shifts and intensity variations. These studies also indicate that QHH and QPT are likely to form H-type aggregates with quenched fluorescence due to dark S_1_ states, while QPP and QTP form J-type aggregates retaining bright S_1_ states and strong fluorescence. The ROS generation ability of QPP and QTP was also well supported by computational studies, which revealed their T_1_–S_0_ energy gaps closely matching the X^3^Σg^–^–b^1^Σg^+^ energy gap of O_2_, facilitating singlet oxygen generation even in the monomeric state. Additionally, studies on π-stacked trimers revealed that aggregation reduced this gap to match the energy required to populate the reactive a^1^Δg state of the O_2_, suggesting enhanced ROS generation in the aggregated form. Experimental studies using DCFDA and DPBF further confirmed that ROS production for QPP was highest. The phosphorescence spectra also aligned with computational predictions, displaying emission from higher triplet states located near the singlet states. These findings suggest that ISC and subsequent ROS generation are facilitated by structural planarization and aggregation. Collectively, these computational studies provide an understanding of how subtle variations in heterocyclic substituents dictate the photophysical properties of fluorophores.

## Supplementary Material



## Notes

Synthetic procedures and characterization data; ^1^H NMR and ^13^C NMR spectra; HRMS data; UV–vis and fluorescence spectra; confocal imaging data; computational study details; figures and tables of QHH, QPP, QPT, and QTP (PDF)
