# Supplementary material for: Pyrrolo[1,2‑a]quinoxaline: A Combined Experimental and Computational Study on the Photophysical Properties of a New Photofunctional Building Block
Source: J Phys Chem B. 2025 Jun 1;129(23):5842–53. doi: 10.1021/acs.jpcb.5c01810 (PMC12169665; doi:10.1021/acs.jpcb.5c01810)
Supplement: Supplementary file 1 [file jp5c01810_si_001.pdf]

Pyrrolo[1,2-*a*] quinoxaline: A Combined Experimental and Computational Study on the Photophysical Properties of a New Photo-functional Building Block

Adarash Kumar Shukla,[a] Savita Choudhary,[a] Dilawar Singh Sisodiya,[b] Ashutosh Mahale,[c] Pravinkumar Vipparthi,[c] Togiti Uday Kumar,[a] Onkar Prakash Kulkarni,[c] Anjan Chattopadhyay,*[b] and Anupam Bhattacharya*[a]

[a.] Department of Chemistry, Birla Institute of Technology and Science-Pilani (Hyderabad Campus), Hyderabad-500078, India.

[b.] Department of Chemistry, Birla Institute of Technology and Science-Pilani (KK Birla Goa Campus), NH 17B, Bypass, Road, Zuarinagar, Sancoale, Goa 403726, India.

[c.] Department of Pharmacy, Birla Institute of Technology and Science-Pilani (Hyderabad Campus), Hyderabad-500078, India.

E-mail: anupam@hyderabad.bits-pilani.ac.in; Tel: +91-40-66303522.

anjan@goa.bits-pilani.ac.in; Tel: +91-832-2580319.

S.No.	Contents	Page No.
1	**Experimental section:**	S4-S6
2	**Fig. S1:** ^{1}H NMR of QHH in DMSO-d_6.<br>**Fig. S2:** ^{13}C NMR of QHH in DMSO- d_6.<br>**Fig. S3:** ^{1}H NMR of QPP in $CDCl_3$.<br>**Fig. S4:** ^{13}C NMR of QPP in $CDCl_3$.<br>**Fig. S5:** ^{1}H NMR of QPT in $CDCl_3$.<br>**Fig. S6:** ^{13}C NMR of QPT in $CDCl_3$.<br>**Fig. S7:** ^{1}H NMR of QTP in DMSO-d_6.<br>**Fig. S8:** ^{13}C NMR of QTP in DMSO-d_6.	S7-S10
3.	**Fig. S9:** HRMS Data of QHH.<br>**Fig. S10:** HRMS Data of QPP.<br>**Fig. S11:** HRMS Data of QPT.<br>**Fig. S12:** HRMS Data of QPT.	S11
4.	**Fig. S13** General representation of PQNs polarity response	S11
5.	**Fig. S14:** UV-visible spectra and Normalised UV-visible spectra of QHH (10µM) in different solvents.<br>**Fig. S15:** UV-visible spectra and Normalised UV-visible spectra of QPP (10µM) in different solvents.<br>**Fig. S16:** UV-visible spectra and Normalised UV-Visible spectra of QPT (10µM) in different solvents.<br>**Fig. S17:** UV-visible spectra and Normalised UV-visible spectra of QTP (20µM) in different solvents.	S12-S13
6.	**Fig. S18:** Fluorescence spectra of QHH (10µM) in different solvents.<br>**Fig. S19:** Fluorescence spectra of QPP (10µM) in different solvents.<br>**Fig. S20:** Fluorescence spectra of QPT (10µM) in different solvents.<br>**Fig. S21:** Fluorescence spectra of QTP (10µM) in different solvents.	S13-S14
	**Fig. S22** Fluorescence spectra of (a) QHH, (b) QPP, (c) QPT, and (d) QTP (10 µM) in different solvents. (λ_{ex} 350 nm for QHH and λ_{ex} 360 nm for QPP, QPT and QTP)	S15
7.	**Table S1:** Absorption and emission wavelength of PQNs	S15

8.	**Table: S2** Solvatofluorochromism parameter of PQNs.	S16
9.	**Fig. S23:** Molar absorptivity of QHH (2,5,10,20 μM) in different solvents at λ_{abs} **=340nm.**<br>**Fig. S24:** Molar absorptivity of QPP (2,5,10,20 μM) in different solvents at λ_{abs} **=360nm.**<br>**Fig. S25:** Molar absorptivity of QPT (2,5,10,20 μM) in different solvents at λ_{abs} **=360nm.**<br>**Fig. S26:** Molar absorptivity of QTP (2,5,10,20 μM) in different solvents at λ_{abs} **=360nm.**	S17-S20
10.	**Fig. S27:** Life-time decay plot of QHH (10μM) in different solvents.<br>**Fig. S28:** Life-time decay plot of QPP (10μM) in different solvents.<br>**Fig. S29:** Life-time decay plot of QPT (10μM) in different solvents.<br>**Fig. S30:** Life-time decay plot of QTP (10μM) in different solvents.	S21-S22
	**Table: S3** Lifetime parameter of PQNs.	S22
11.	**Table S4:** Photophysical parameter of PQNs in solid and solution states	S23
12.	**Fig. S31:** UV-visible spectra of QHH (10μM) in dioxane and water solvent.<br>**Fig. S32:** UV-visible spectra of QPP (10μM) in dioxane and water solvent.<br>**Fig. S33:** UV-visible spectra of QPP (10μM) in dioxane and water solvent.<br>**Fig. S34:** UV-visible spectra of QTP (10μM) in dioxane and water solvent.	S23-S24
13.	**Fig. S35:** Detailed analysis of aggregation of formation of PQNs.	S24
14.	**Fig. S36:** DLS spectra of PQNs in water at 10μM concentration.	S25
15.	**Fig. S37** Fluorescence lifetime decay of (a) QHH, (b) QPP, (c) QPT, and (d) QTP with dioxane and water using λ_{ex}= 375 nm.<br>**Fig. S38:** Fluorescence spectra of QHH (10 μM) in solid and solution state.<br>**Fig. S39:** Fluorescence spectra of QPP (10μM) in solid and solution state using λ_{ex}=370 nm.<br>**Fig. S40:** Fluorescence spectra of QPT (10μM) in solid and solution state using λ_{ex}=370 nm.<br>**Fig. S41:** Fluorescence spectra of QTP (10μM) in solid and solution state using λ_{ex}=370 nm.	S25-S27
16.	**Fig. S42:** Lifetime decay plot of QHH (10μM) in solid and solution state using λ_{ex}=350 nm.<br>**Fig. S43:** Lifetime decay plot of QPP (10μM) in solid and solution state using λ_{ex}=370 nm.<br>**Fig. S44:** Lifetime decay plot of QPT (10μM) in solid and solution state using λ_{ex}=370 nm.<br>**Fig. S45:** Lifetime decay plot of QTP (10μM) in solid and solution state using λ_{ex}=370 nm.	S27-S28
17.	**Fig. S46:** QTP-Crystal packing mode, Hirshfeld Surface analysis of QTP crystal for weak interaction quantification.	S29-S30
18.	**Fig. S47:** PXRD spectra of PQNs.	S31
19.	**Figure 48:** a) Fluorescence spectra of (a) QHH, (b) QPP, (c) QPT, and (d) QTP in different pH using λ_{ex}= 370 nm.	S31
20.	**Fig. S49:** Lifetime decay plot of QHH (10μM) in different pH.<br>**Fig. S50:** Lifetime decay plot of QPP (10μM) in different pH.<br>**Fig. S51:** Lifetime decay plot of QPT (10μM) in different pH.<br>**Fig. S52:** Life-time decay plot of QTP (10μM) in different pH.	S32-S33
21.	**Figure S53:** MTT data of PQNs in RAW 264.7 cells [control-1 for QHH, control-2 for QPP, control-3 for QTP, control-4 for QPT].	S33

	**Figure S54:** MTT data of PQNs in CAL 20 cells [control-1 for QHH, control-2 for QPP, control-3 for QTP, control-4 for QPT].	
22.	**Fig. S55** (a) CLSM imaging of PQNs (10 μM, λ_{ex}= 405 nm, λ_{em}= 415-550 nm) in RAW 264.7 cells and (b) CAL 27 cells incubated with PQNs (10 μM, λ_{ex}= 405 nm, λ_{em}= 415-550 nm)and DRAQ5 (5 μM, nucleus tracker, λ_{ex} = 633 nm, λ_{em} = 650−700 nm). Scale bar=50μm.<br>**Fig. S56:** Confocal imaging comparison of QHH (10μM) with different organelle staining biomarkers.<br>**Fig. S57:** Confocal imaging comparison of QPP (10μM) with different organelle staining biomarkers.<br>**Fig. S58:** Confocal imaging comparison of QPT (10μM) with different organelle staining biomarkers.<br>**Fig. S59:** Confocal imaging comparison of QTP (10μM) with different organelle staining biomarkers.	S34-S36
23.	**Fig. S60:** ROI-based image analysis a) FLIM imaging of QHH (10μM) in RAW cell, b) FLIM imaging of QHH (10μM) under inflammation condition in RAW cell, c) FLIM imaging of QTP (10μM) in RAW cell, FLIM imaging of QTP (10μM) under inflammation condition in RAW cell.	S36-S38
24.	**Table S5a.** TDDFT-predicted absorption peak positions in dioxane and water<br>**Table S5b.** TDDFT-predicted emission peak positions in dioxane and water	S39
25.	**Fig. S61** Fluorescent S_1 minima geometries with charge distributions (orbitals of S_0 and S_1) of (a) QHH (b) QPP (c) QPT (d) QTP<br>**Fig. S62:** S_0 minima geometries of QHH, QPP, QPT, and QTP.<br>**Fig. S63:** S_1 minima geometry of QTP- S_1'.<br>**Fig. S64:** Structures of the optimized S_1 of π-stacked dimers of QPP and QTP.<br>**Fig. S65:** slip-stacked trimer of QTP-S_1.<br>**Fig. S66:** The S2\T3\T4 degenerate geometries of QPP (top left), QPT (top right), and QTP (bottom).	S39-S41
26.	**Table S6:** Spin-Orbit Coupling (SOC) parameters (in cm^{-1}) for S_0 and S_1 optimized geometries of QHH, QPP, QPT, and QTP.	S42-S43
27.	**Table S7: a)** TDDFT-predicted phosphorescence peak positions (in water) in nm **b) T_2(min) /T_3(min) – T_1 energy gap in eV c)** $S_{1\text{-}min}$ – $T_{1/2/3\text{-}min}$ gap in eV **d)** Emission Dimer (optimized) with oscillator strengths (in water) in nm **e)** Emission of slip-stack dimer and trimer of QTP-S1 **f)** Dipole moments (Debye).	
28.	**Fig. S67.** Fluorescence and phosphorescence emission spectra of QHH, QPP, QPT, and QTP.<br>**Fig. S68:** UV-visible spectra of DPBF (25μM) photodegradation in dioxane using white light irradiation.<br>**Fig. S69:** UV-visible spectra of DPBF ((25μM) with QPP (5μM) using white light irradiation.<br>**Fig. S70:** UV-visible spectra of DPBF ((25μM) and NaN_3 (50μM)with QPP (5μM) using white light irradiation.<br>**Fig. S71:** UV-visible spectra of DPBF (25μM) in the presence of QHH (5 μM) irradiating with white light source**.**<br>**Fig. S72:** UV-visible spectra of DPBF (25μM) in the presence of QPT (5 μM) irradiating white light source**.**<br>**Fig. S73:** UV-visible spectra of DPBF (25μM) in the presence of QTP (5 μM) irradiating white light source**.**<br>**Fig. S74:** UV-visible spectra of DPBF (25μM) in the presence of QHH (5 μM) and singlet oxygen quencher irradiating white light source**.**	S44-S46

Experimental section:

Nuclear magnetic resonance (NMR) spectroscopy:

^{1}H NMR and ^{13}C NMR spectra were recorded using Bruker Avance III NMR spectrometers. The residual solvent signal was used as an internal standard, and chemical shifts (δ) were reported in parts per million (ppm). All NMR measurements were carried out in DMSO-d_6/$CDCl_3$ at room temperature.

High-resolution mass spectrometry (HRMS):

High-resolution mass spectrometry (HRMS) data were obtained using Bruker MicrOTOF-Q-II mass spectrometer.

Melting point measurements:

The melting points of the compounds were measured using a Digital Melting Point Apparatus (Kruss. SN. 1804140001). Compounds were loaded in the capillary glass tubes.

Field emission scanning electron microscopy (FE-SEM): The morphologies of the PQNs' dispersions were observed using Carl Zeiss's (Ultraplus) field emission scanning electron microscope. Samples for microscopy were prepared by drop-casting of PQNs dispersion (~ 10µL) on Si wafer. After drying under a vacuum, all samples were coated with a thin layer of sputtered gold before imaging.

Steady-state absorption spectroscopy: Jasco spectrophotometer was used to measure the UV-visible absorption spectra. 10 mm path-length quartz cuvettes were used for recording the spectra. The optical density was kept low (< 0.1) to avoid inner filter effects. The solid-state absorption spectra were recorded using the same spectrometer equipped with a diffuse reflectance integrating sphere attachment (DRA).

Steady-state fluorescence spectroscopy: Jobin Yvon Horiba Model Fluorolog-3 instrument was used for all the steady-state fluorescence measurements. The slit width for all experiments was maintained at 1.5 nm. λ_{ex} 350 nm for QHH and λ_{ex} 360 nm for QPP, QPT and QTP.

The time-correlated single-photon counting (TCSPC):

HORIBA spectrometer was used for the time-resolved fluorescence measurements. 370/340 nm delta diode lasers were used as the excitation sources.

$K_r = \phi_f / \tau_{avg}$ (ns), $K_{nr} = (1-\phi_f) / \tau_{avg}$ (ns) (K_r = radiative decay, K_{nr} = non-radiative)

Fluorescence Quantum Yield (ϕ_f) Measurements: The fluorescence quantum yield of PQNs in solution was estimated using Quinine sulfate in 1N H_2SO_4 (Φ_f = 54%) as the reference dye using the following equation.

$$\Phi_{f,x} = \Phi_{f,s} \times I_X/I_S \times A_S/A_X \times (n_X/n_S)^2 .$$

Where Φ_f is the fluorescence quantum yield, subscript x denotes the sample, and subscript s refers to the standard. I denote integral fluorescence; n refers to the refractive index of the solvent used in the measurements, and A is the absorption at the excitation wavelength given by the following equation.

Powder X-ray diffraction (PXRD) analysis: The XRD measurement was performed using a PANalytical Empyrean X-ray diffractometer in the Bragg-Brentano geometry using a CuKα1 (λ = 1.5405 Å) radiation and Ni beta filter was used to obtain only the K_α radiation. The powder samples were mounted on the silicon zero background holder by dropping powder and then flattening the sample surface with a glass plate. The generator power is set at 45 kV and 40 mA to measure the powder X-ray data. The data were collected using the Pixcel3D detector in the angular range of 2 to 45° in the 2θ range within steps of 0.1°.

Preparation of PQN aggregates: A stock solution of 0.1 mM **of PQNs** was prepared in 5 mL of DMSO. 2 μlt was rapidly added to the binary solvent mixture of dioxane-water under sonication for 1 min. The pH of the dispersion medium was monitored using a calibrated pH meter (Oakton). The pH meter was calibrated using standard pH buffer solutions = 12, 7, and 4.

Cell Culture:

MTT (carried out in dark): In-vitro experiments on cell line RAW 264.7/CAL 20

RAW 264.7 (monocyte/macrophage cell line)/CAL 27 cells (oral adenosquamous carcinoma cell line) were seeded at 7,000 cells per well in a 96-well plate and incubated for 24 hours at 37°C with 5% CO_2. PQNs were then treated with eight concentrations (100, 50, 25, 12.5, 6.25, 3.12, 1.56, and 0.78 μM) for 24 hours. After treatment, 0.5 mg/mL MTT reagent was added, and the cells were incubated for 3 hours. Formazan crystals were solubilized with 100 μL DMSO, and absorbance was measured at 562 nm to assess cell viability. The percentage of cell viability was calculated relative to control wells, and results were graphed using GraphPad Prism.

Note: MTT experiment was performed in dark.

Confocal Imaging and Organelle Staining:

For initial confocal imaging with RAW264.7 and CAL 27 cells , were seeded onto a coverslip in a 12-well plate and allowed to incubate for 24 hours. The cells were then incubated with PQNs (10 μM) for 10 minutes. Further the cells were washed with PBS, and incubated with DRAQ5 for 5 mins to stain nucleus. Without further washing cells were, fixed, and mounted for imaging.

For inducing inflammation, cells were treated with lipopolysaccharides (LPS, 1 nM) for 3 hours and finally with PQNs (10 μM) for 10 minutes.

Organelle Marker	Organelle stained	Concentration	Excitation and Emission	Incubation Time
**DRAQ5**	Nuclues	5 μM	λ_{ex} = 633 nm, λ_{em} = 650−700 nm	5 mins
**Lyso-Tracker Red**	Lysosomes	100 nM	λ_{ex} = 514 nm, λ_{em} = 550−600 nm	3 hours
**Nile Red**	Lipid droplet	5 μM	λ_{ex} = 488 nm, λ_{em} = 550− 600 nm	30 mins
**Mito-Tracker red**	Mitochondria	20 nM	λ_{ex} = 514 nm, λ_{em} = 550− 610 nm	30 mins
**DCFDA (5(6)-Carboxy-2′,7′ dichlorofluoresceindiacetate)**	For ROS detection	10 μM	λ_{ex} = 488 nm, λ_{em} = 500− 550 nm	30 mins

Note: Except for DRAQ5 all the organelle markers were incubated first followed by PQNs (10 μM)

Fluorescence Lifetime Imaging microscopy: Fluorescence lifetime imaging was performed in PicoQuant Microtime 200 using a 405 nm excitation laser source, without a bandpass filter, during a long pass filter. Confocal slides were used for FLIM imaging.

Preparation of Sample for Phosphorescence: Phosphorescence was recorded in the Horiba make instrument. PQNs (100μM) were dissolved in DCM drop-casted in a glassy coverslip, keeping excitation at 340 nm for QHH and 350nm. phosphorescence emission range in 390-700 nm at 90 K.

Singlet-oxygen detection and generation: The UV-visible study of PQNs with DPBF solution was performed to understand singlet-oxygen generation. The DPBF (50μM) solution was freshly prepared in the dark, and the control photodegradation experiment was performed without PQNs solution in

dioxane. PQNs (10µM) solution was incubated with DPBF (50µM) under the dark, purged with an oxygen balloon, and with 20-second intervals using a white light exciting source.

Synthesis and Characterization of PQNs:

The synthesis of PQNs was done using previously reported methods.[1,2]

Pyrrolo[1,2-a]quinoxaline(QHH) ^{1}H NMR (400 MHz, DMSO) δ 8.88 (s, 1H), 8.47-8.46 (m, 1H), 8.29 (dd, J= 8.4 1.2 Hz, 1H), 7.88 (dd, J= 8, 1.6 Hz, 1H), 7.61 (td, J= 8, 1.6 Hz, 1H), 7.51-7.47 (m, 1H), 7.01-7.00 (m, 1H), 6.95-6.94 (m, 1H). ^{13}C NMR (101 MHz, DMSO) δ 146.23, 135.78, 129.92, 128.46, 128.03, 126.32, 125.74, 116.28, 115.30, 114.52, 107.91. Calculated/Experimental mass 169.0766/169.0774.

2,4-diphenylpyrrolo[1,2-a]quinoxaline(QPP)[ref] ^{1}H NMR (400 MHz, $CDCl_3$) δ 8.20 (d, J=1.2Hz, 1H), 8.00-7.95 (m, 3H), 7.88-7.86 (m 1H), 7.65-7.63 (m, 2H), 7.52-7.48 (m, 3H), 7.47-7.41(m, 2H), 7.39-7.34 (m, 2H), 7.26-7.22(m 1H), 7.18 (m, 1H). ^{13}C NMR (101 MHz, CDCl3) δ 138.39, 136.36, 134.25, 130.41, 129.90, 128.97, 128.69, 128.63, 127.66, 127.16, 126.23, 125.43, 113.62, 111.30, 106.07. Calculated/Experimental mass 321.1386/321.1408.

2-phenyl-4-(thiophen-2-yl)pyrrolo[1,2-a]quinoxaline(QPT) ^{1}H NMR (400 MHz, $CDCl_3$) δ 8.25 (d, J= 1.2Hz, 1H), 8.02-7.99 (m, 2H), 7.92 (dd, J= 8, 1.2Hz, 1H), 7.76-7.73 (m, 2H), 7.57 (dd, J= 4, 1.2Hz, 1H) 7.54-7.50 (m, 2H), 7.48-7.44 (m, 3H) 7.32 (tt, J= 15.2, 1.2Hz, 1H), 7.26-7.24 (m, 1H). Calculated/Experimental mass 327.0956/327.0971.

4-phenyl-2-(thiophen-2-yl)pyrrolo[1,2-a]quinoxaline (QTP) H NMR (400 MHz, DMSO) δ 8.92 (d, J= 1.2Hz, 1H), 8.39 (dd, J= 8.4, 1.2 Hz, 1H), 8.05-8.03 (m, 2H),7.95 (dd, J= 8, 1.2Hz, 1H), 7.67-7.60 (m 4H), 7.55-7.50 (m, 3H), 7.24-7.23 (m, 1H), 7.15-7.13 (m, 1H). ^{13}C NMR (101 MHz, DMSO) δ 153.22, 138.11, 137.27, 136.02, 130.52, 130.11, 129.13, 128.93, 128.61, 126.73, 126.20, 125.35, 125.16, 124.50, 123.92, 115.36, 113.60, 105.69. Calculated/Experimental mass 327.0956/327.0973.

Reference: (1). Togiti, U. K., Shukla, A. K., & Bhattacharya, A. (2021). Pyrrolo [1, 2-a] quinoxalines from chalcones: An alternate route. Tetrahedron Letters, 70, 153008. (2). Reeves, J.T., Fandrick, D.R., Tan, Z., Song, J.J., Lee, H., Yee, N.K., Senanayake, C.H. (2010). Copper-catalysed annulation of 2-formyl azoles with *o*-aminoiodoarenes. Journal of Organic Chemistry, 75(3), 992-994.

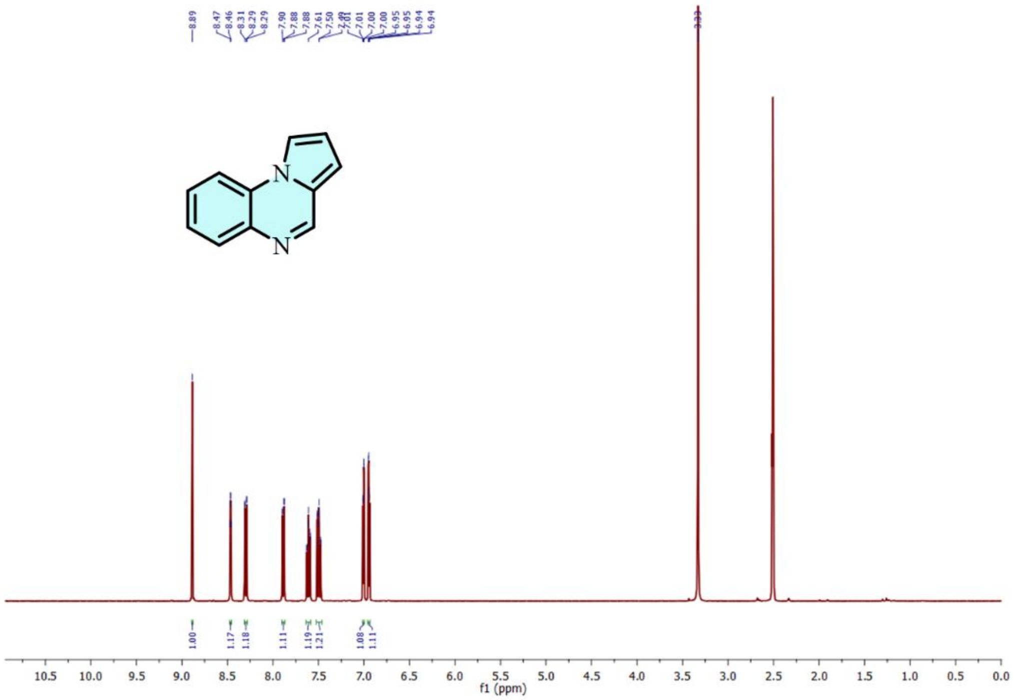

Fig. S1: ^{1}H NMR of QHH in DMSO-d_6.

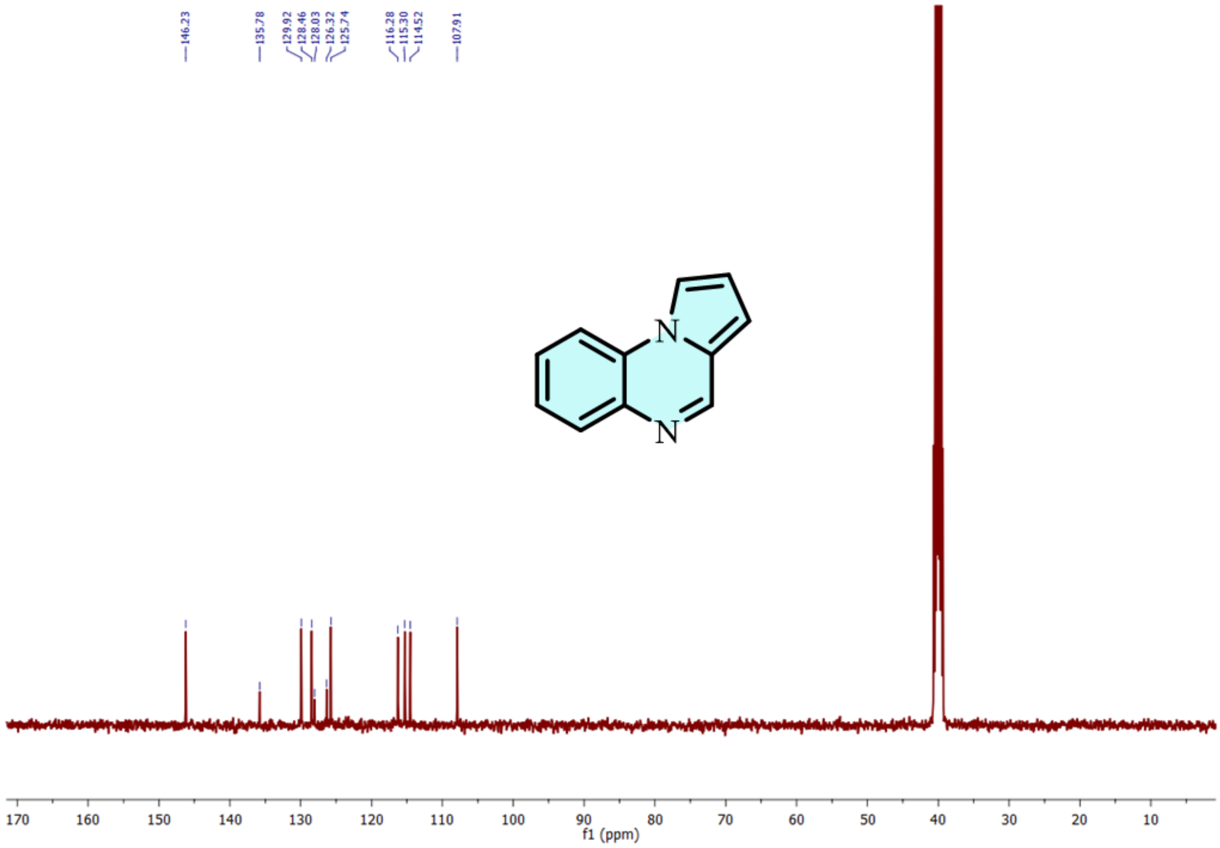

Fig. S2: ^{13}C NMR of QHH in DMSO- d_6.

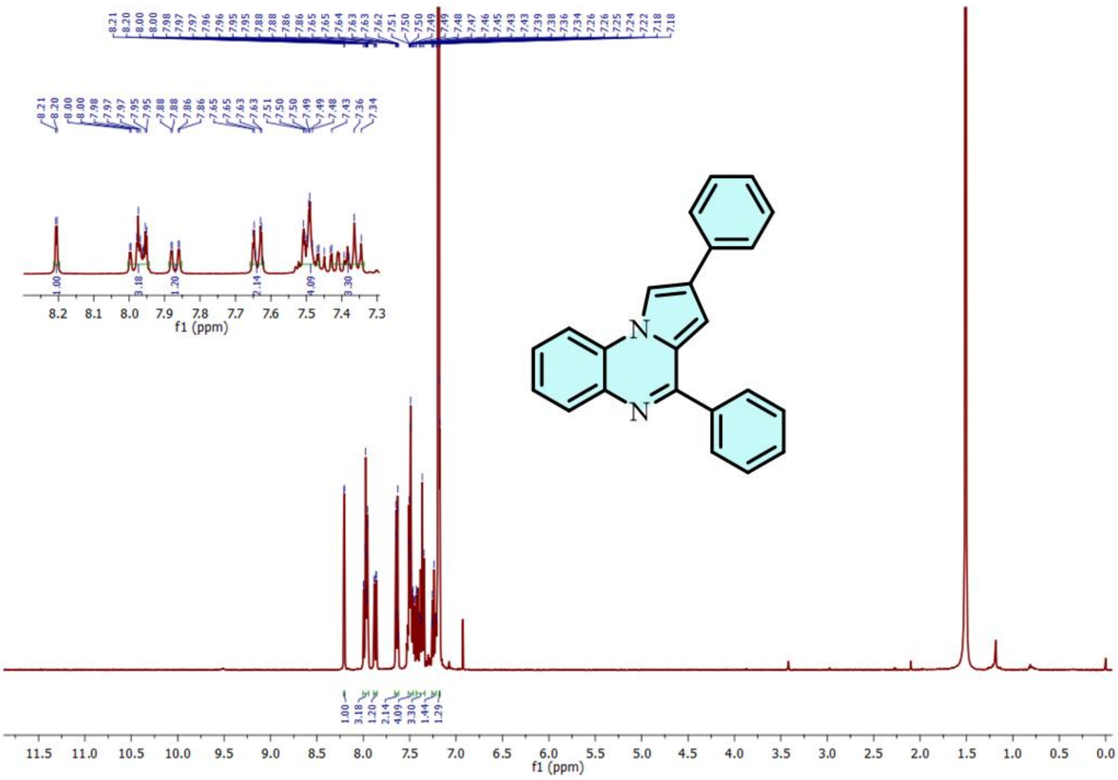

Fig. S3: ^{1}H NMR of QPP in $CDCl_3$.

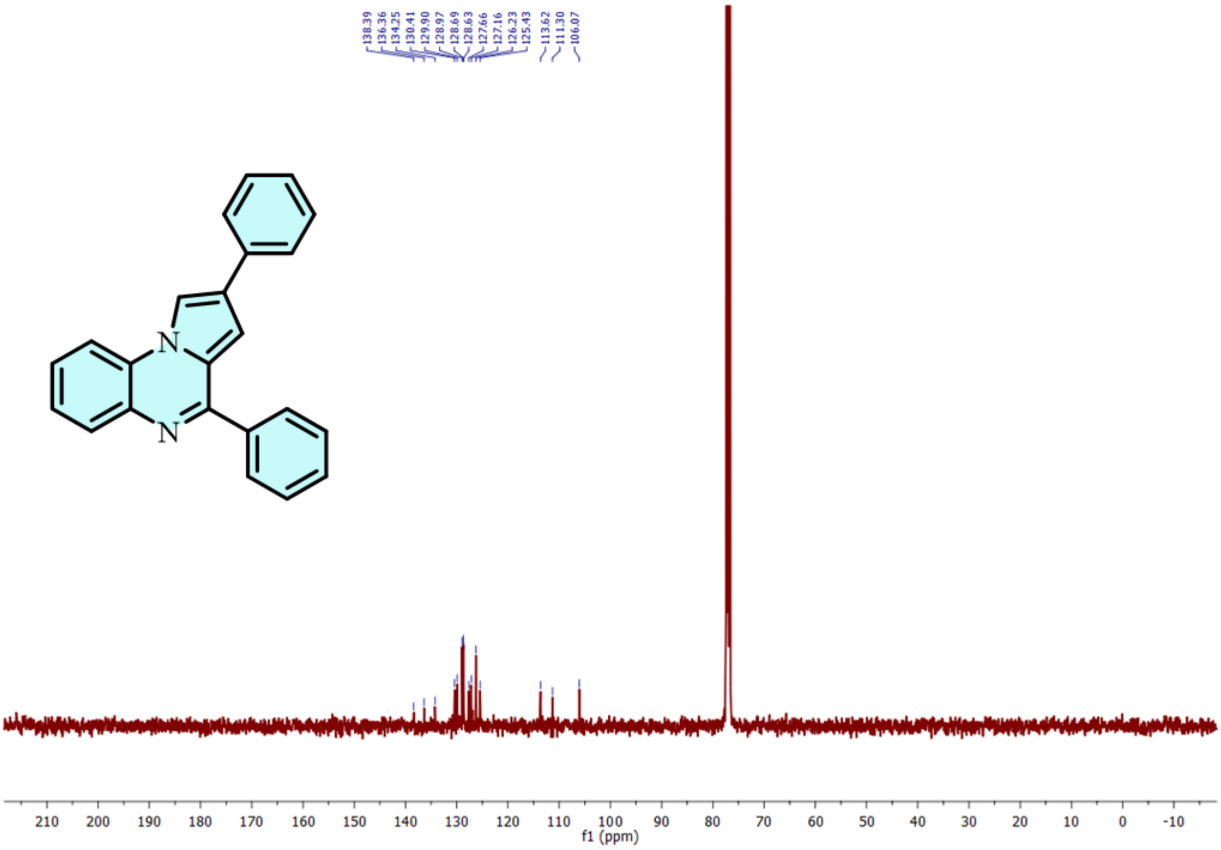

Fig. S4: ^{13}C NMR of QPP in $CDCl_3$.

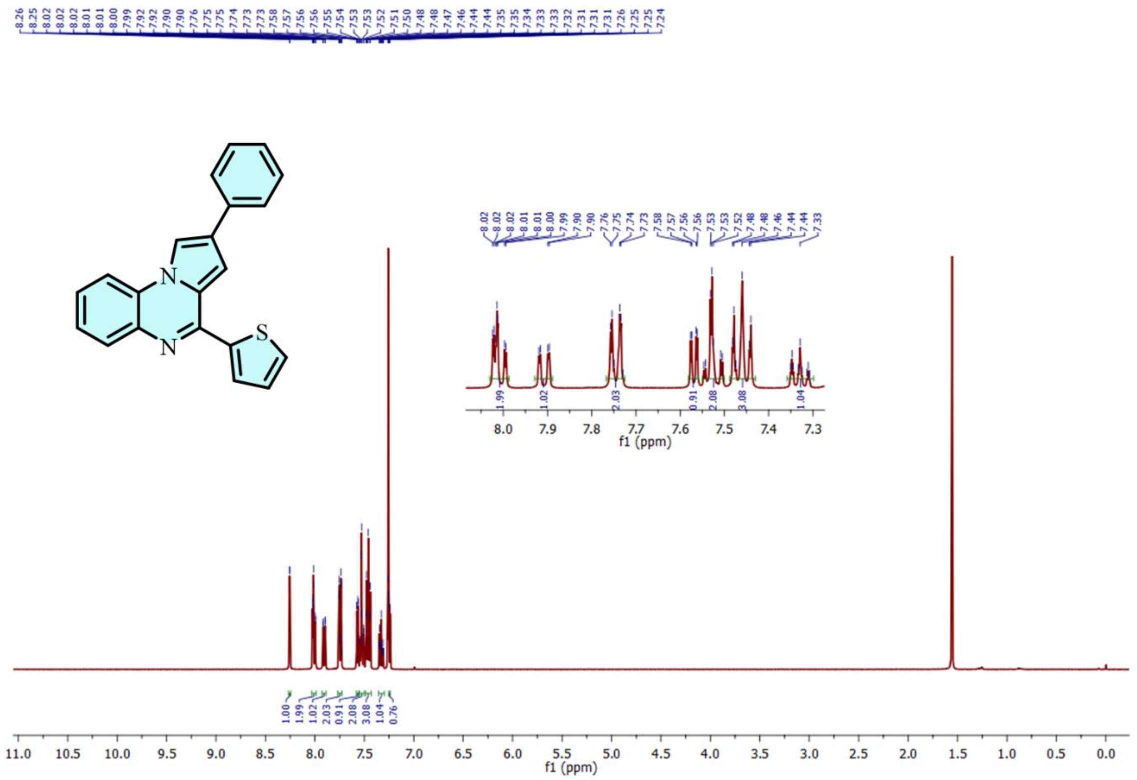

Fig. S5: ^{1}H NMR of QPT in $CDCl_3$**.**

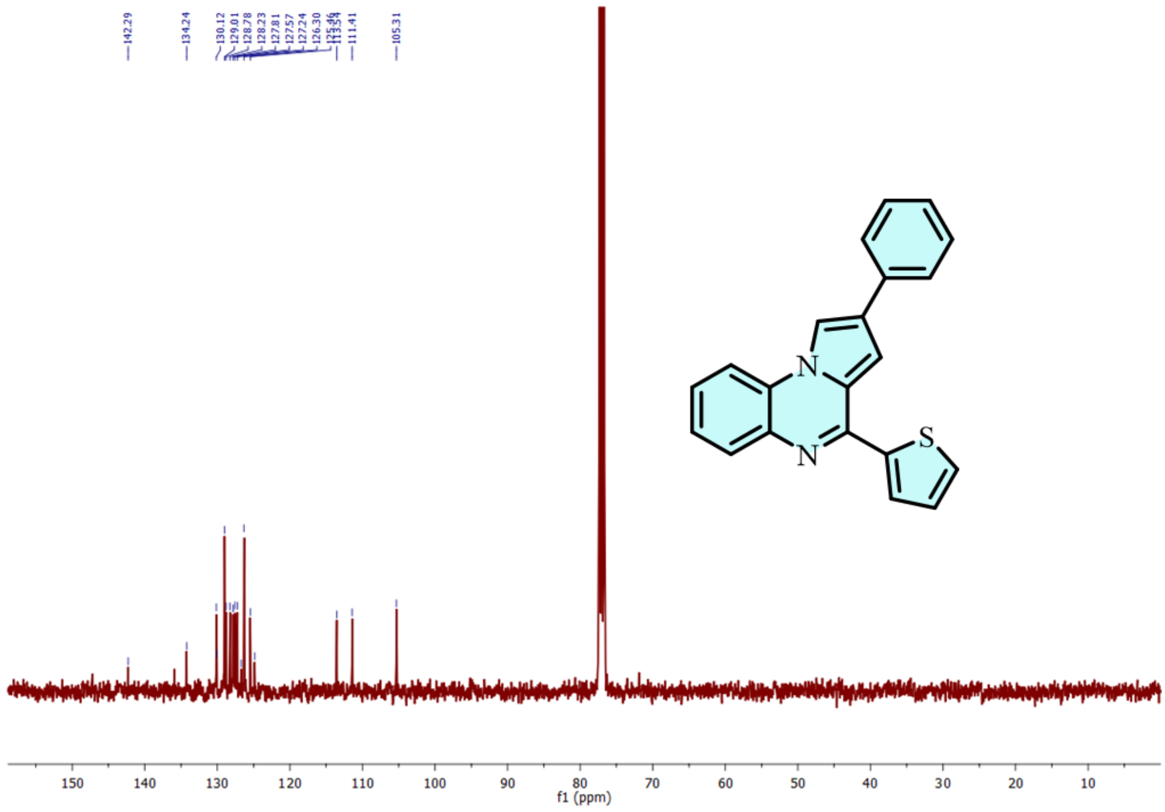

Fig. S6: ^{13}C NMR of QPT in $CDCl_3$**.**

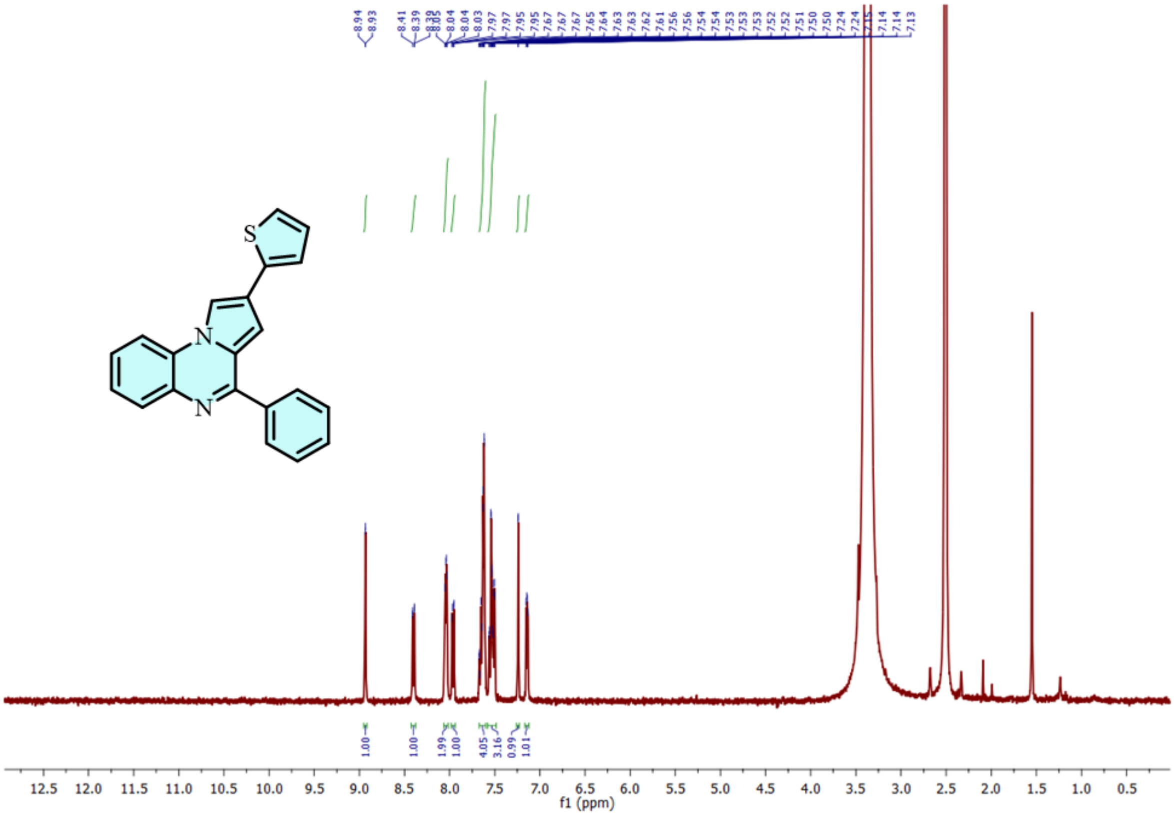

Fig. S7: ^{1}H NMR of QTP in DMSO-d$_6$.

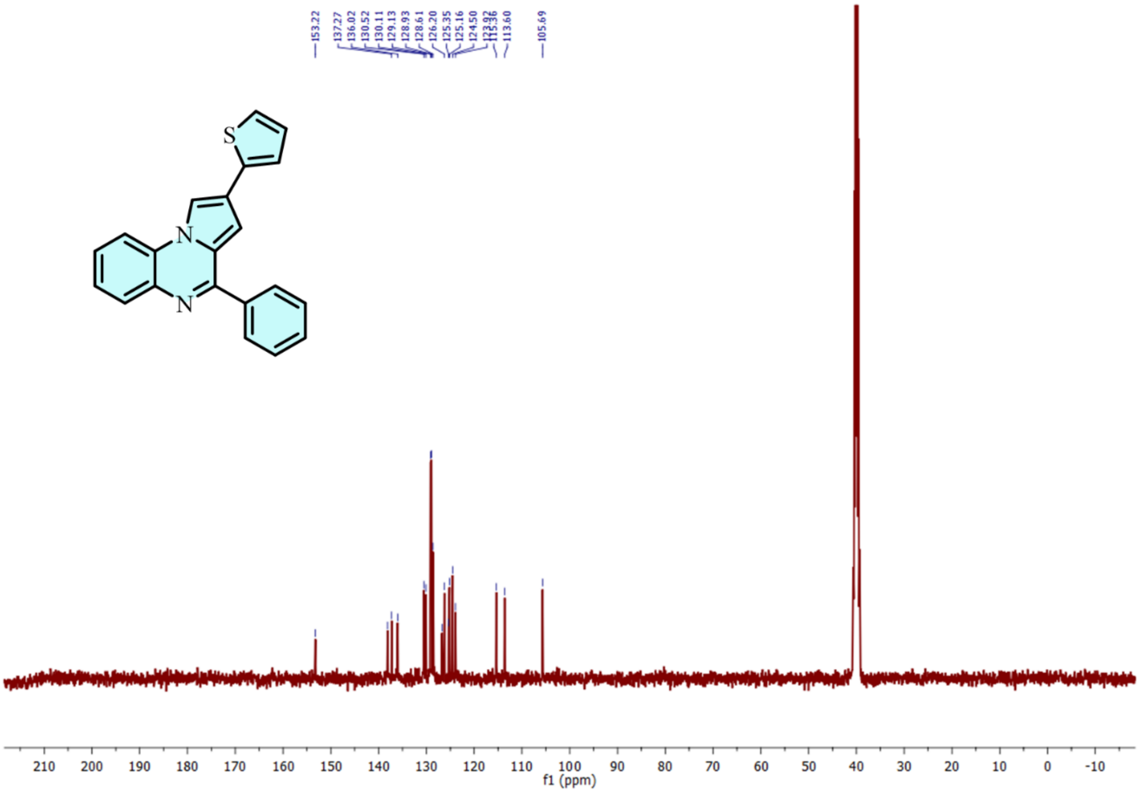

Fig. S8: ^{13}C NMR of QTP in DMSO-d$_6$.

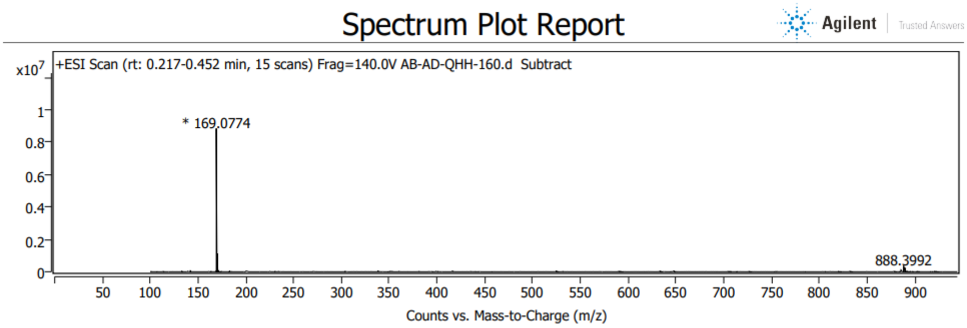

Fig. S9: HRMS Data of QHH.

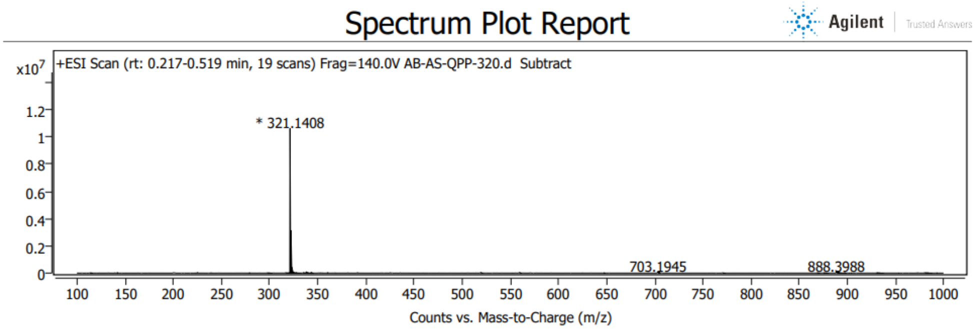

Fig. S10: HRMS Data of QPP.

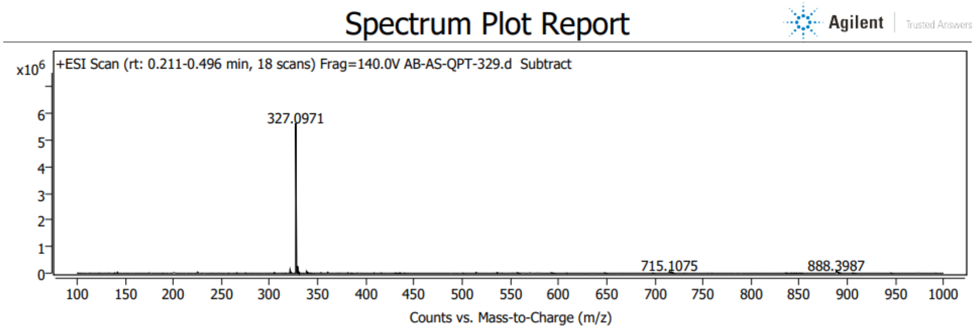

Fig. S11: HRMS Data of QPT.

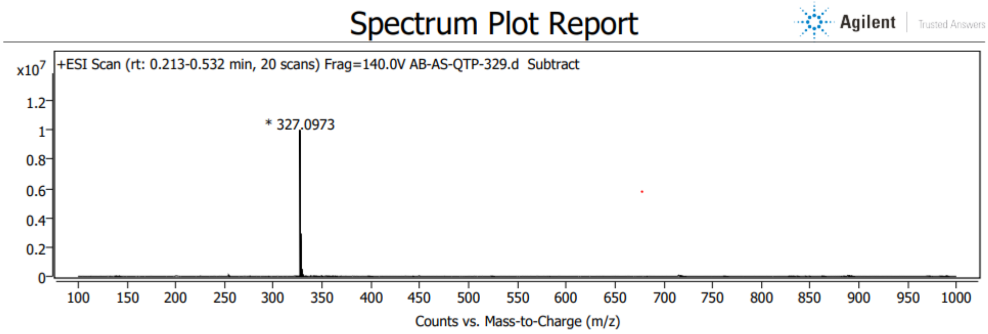

Fig. S12: HRMS Data of QPT.

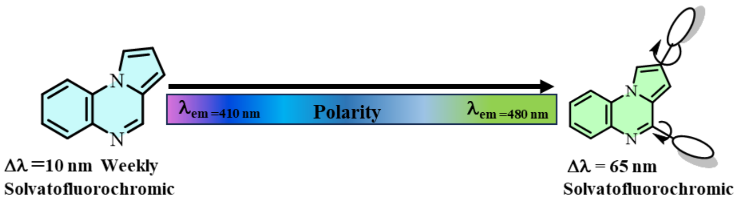

Fig. S13 General representation of PQNs polarity response (Spelling of weak in diagram)

Solvatochromism

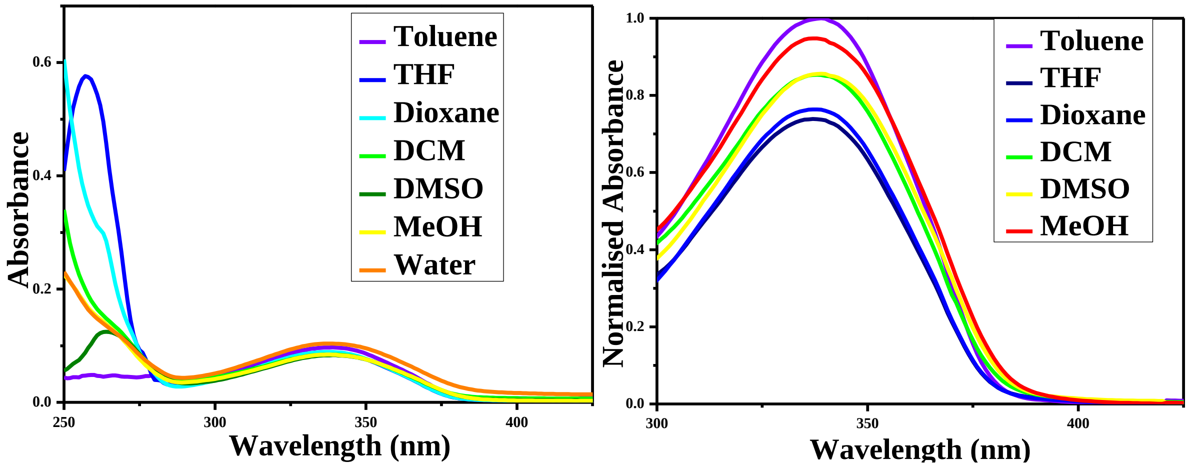

Fig. S14: UV-visible spectra and Normalised UV-visible spectra of QHH (10µM) in different solvents.

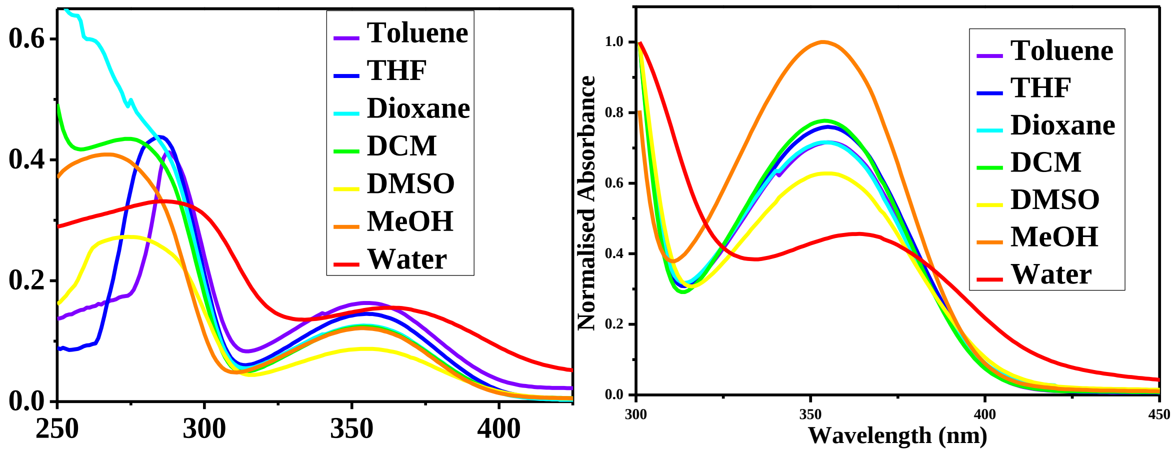

Fig. S15: UV-visible spectra and Normalised UV-visible spectra of QPP (10µM) in different solvents.

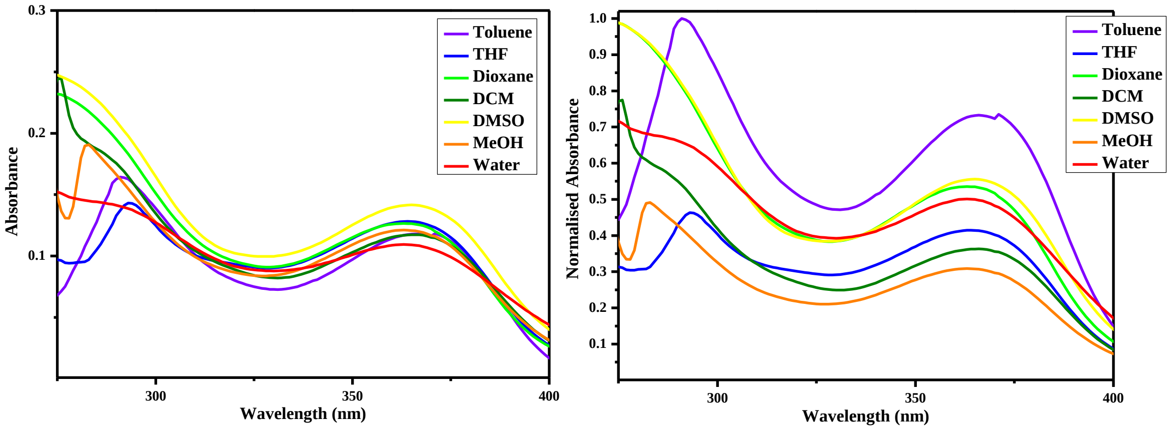

Fig. S16: UV-visible spectra and Normalised UV-Visible spectra of QPT (10µM) in different solvents.

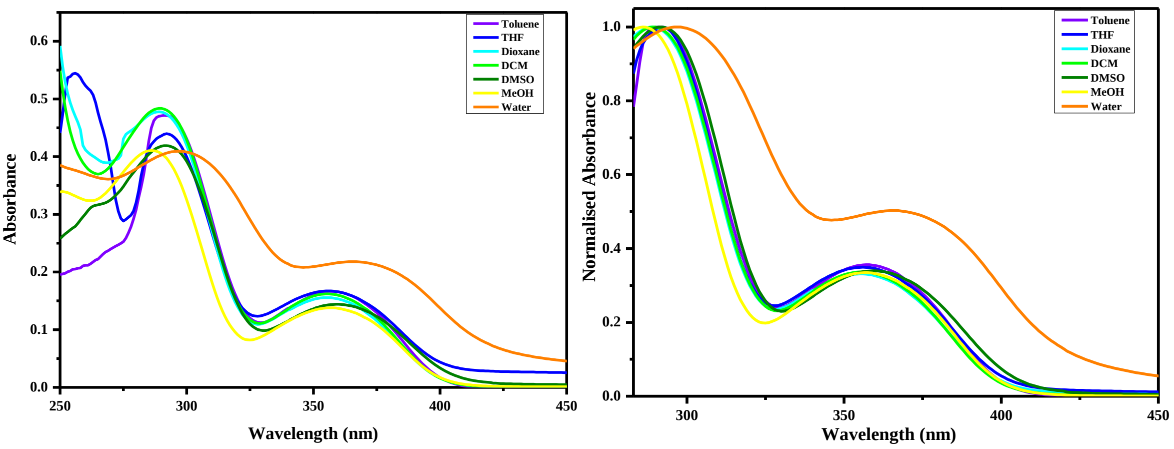

Fig. S17: UV-visible spectra and Normalised UV-visible spectra of QTP (20µM) in different solvents.

Solvatofluorochromism

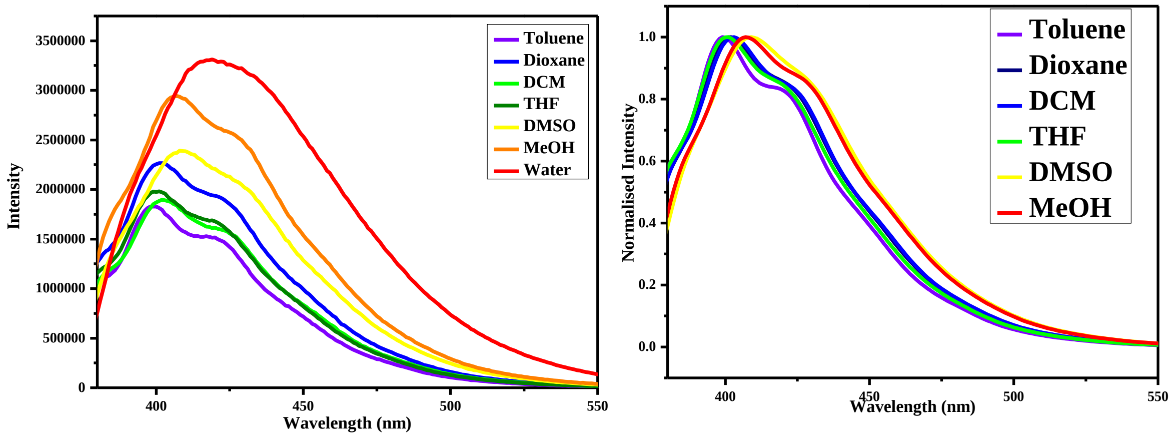

Fig. S18: Fluorescence spectra of QHH (10µM) in different solvents.

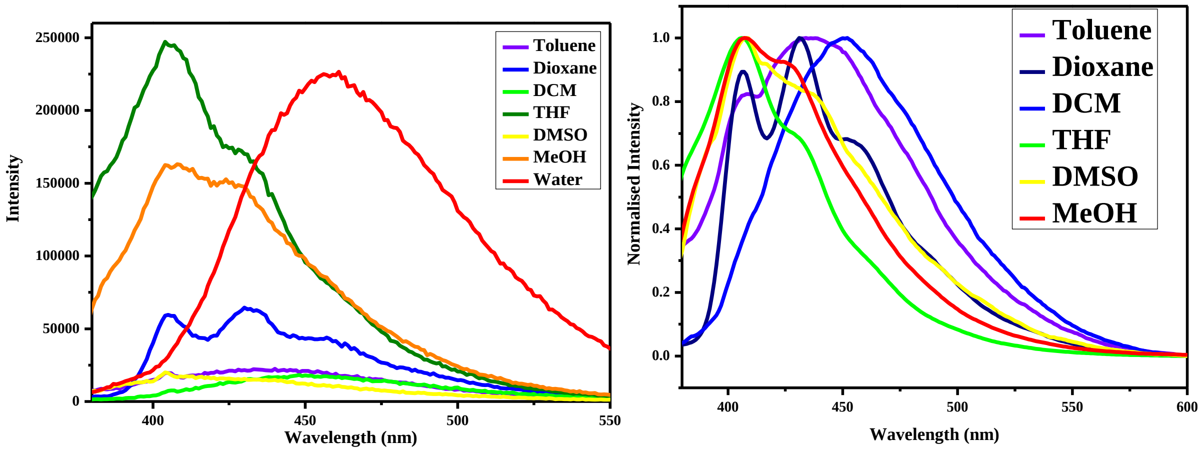

Fig. S19: Fluorescence spectra of QPP (10µM) in different solvents.

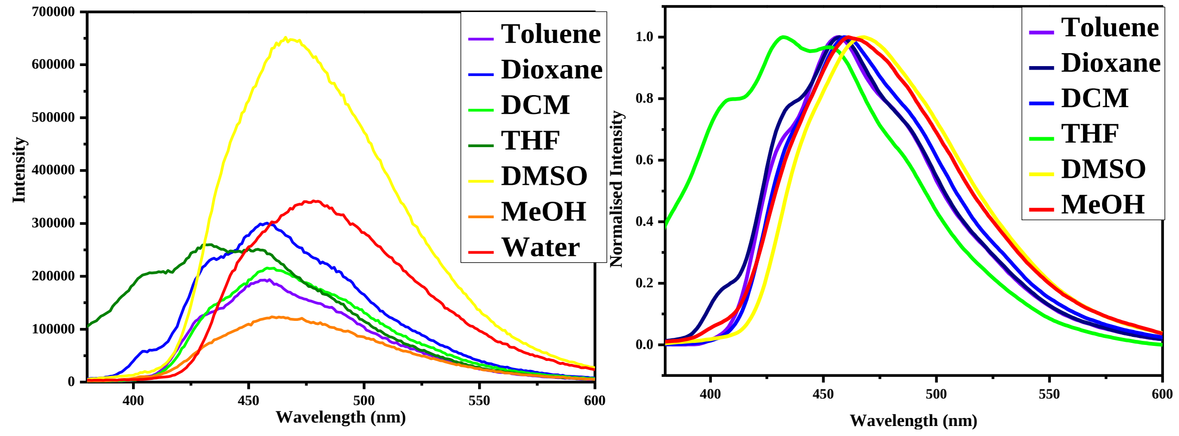

Fig. S20: Fluorescence spectra of QPT (10µM) in different solvents.

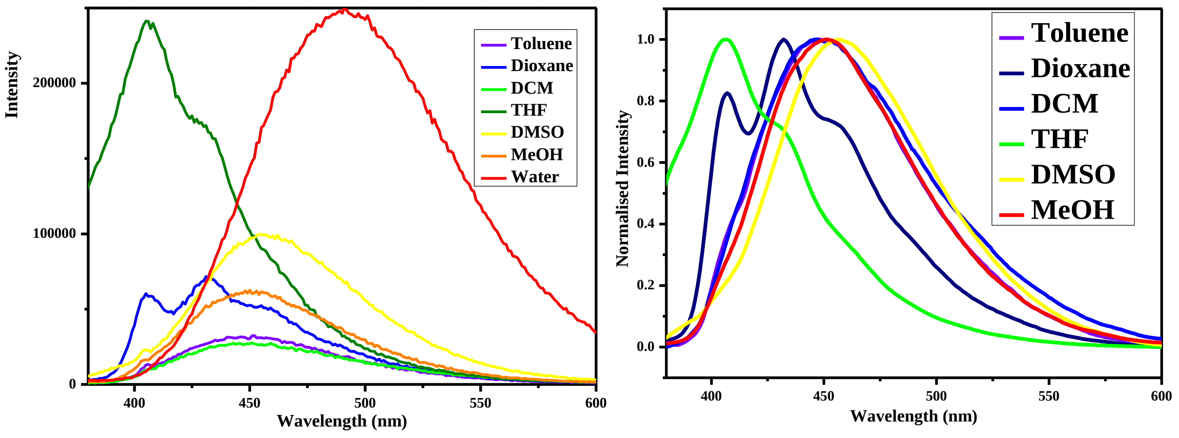

Fig. S21: Fluorescence spectra of QTP (10µM) in different solvents.

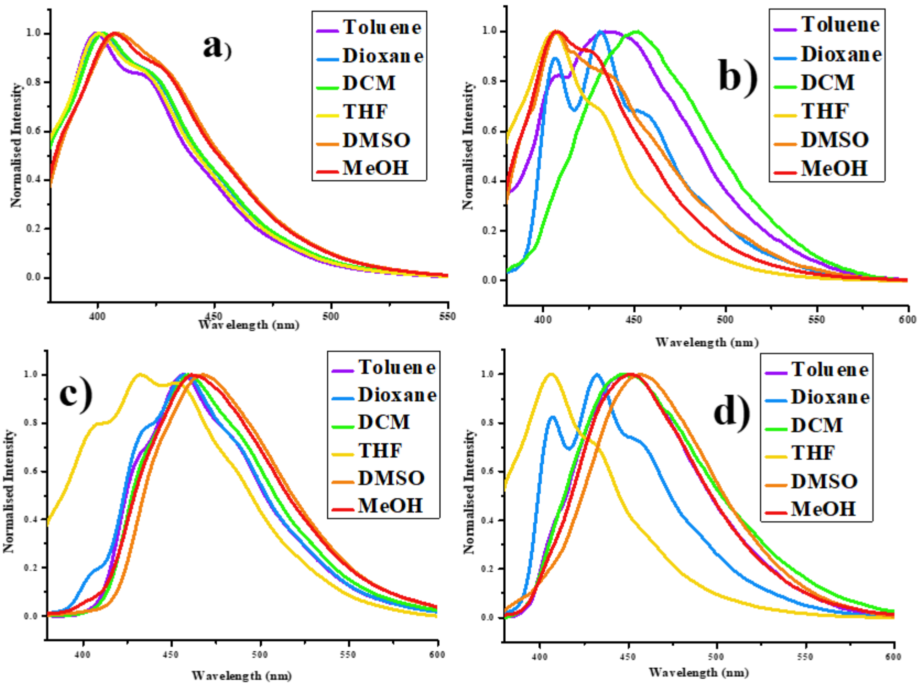

Fig. S22 Fluorescence spectra of (a) QHH, (b) QPP, (c) QPT, and (d) QTP (10 µM) in different solvents. (λ_{ex} 350 nm for QHH and λ_{ex} 360 nm for QPP, QPT and QTP)

Table S1: Absorption and emission wavelength of PQNs

**Solvent**	**QHH (λ_{abs}nm)**	**QHH (λ_{em}nm)**	**QPP (λ_{abs}nm)**	**QPP (λ_{em}nm)**	**QPT (λ_{abs}nm)**	**QPT (λ_{em}nm)**	**QTP (λ_{abs}nm)**	**QTP (λ_{em}nm)**
**Toluene**	340	398	356	440	362	459	357	451
**Dioxane**	338	402	355	430	360	458	355	431
**THF**	337	401	354	404	348	431	358	405
**DCM**	337	402	354	448	364	458	355	443
**DMSO**	338	408	355	404	366	466	361	454
**MeOH**	337	407	354	408	360	460	357	450

Table: S2 Solvatofluorochromism parameter of PQNs.

Solvent	Δf	ε	ϕ	τ_{avg} (ns)	Relative brightness	Stokes shift (cm^{-1})
**QHH**						
**Toluene**	0.0126	8980	6.04	2.58	54239.2	185185
**Dioxane**	0.0204	9230	0.85	1.10	7845.5	156250
**THF**	0.0208	9330	0.94	0.84	8770.2	156250
**DCM**	0.2171	8420	5.82	5.94	49004.4	153846
**DMSO**	0.2632	8110	101.25	5.72	821137.5	142857
**MeOH**	0.308	8580	11.18	6.19	95924.4	142857
**Water**	0.3200	8340	1.87	11.21	15595.8	117647
**QPP**						
**Toluene**	0.0126	13720	0.09	0.20	1234.8	119047
**Dioxane**	0.0204	12020	0.03	0.41	360.6	133333
**THF**	0.0208	14160	0.07	0.69	991.2	200000
**DCM**	0.2171	11770	0.06	0.22	706.2	106382
**DMSO**	0.2632	10250	0.68	0.55	6970	204081
**MeOH**	0.308	11170	0.46	0.29	5138.2	185185
**Water**	0.3200	13230	0.11	1.59	1455.3	106382
**QPT**						
**Toluene**	0.0126	1770	6.92	0.53	12248.4	103092
**Dioxane**	0.0204	1520	0.68	0.65	1033.6	102040
**THF**	0.0208	1280	0.36	0.79	460.8	120481
**DCM**	0.2171	1260	5.35	0.48	6741	106382
**DMSO**	0.2632	1480	184.31	0.88	272778.8	100000
**MeOH**	0.308	1210	2.92	0.39	3533.2	100000
**Water**	0.3200	1080	0.78	0.73	842.4	114942
**QTP**						
**Toluene**	0.0126	18110	0.13	0.24	2354.3	106382
**Dioxane**	0.0204	13760	0.024	0.39	330.24	131578
**THF**	0.0208	14060	0.07	0.65	984.2	212765
**DCM**	0.2171	15490	0.07	0.19	1084.3	113636
**DMSO**	0.2632	13760	3.09	0.42	42518.4	107526
**MeOH**	0.308	16220	0.18	0.26	2919.6	107526
**Water**	0.3200	18340	0.07	2.39	1283.8	86956

Where, Δf= is the change in orientation polarizability ε=molar absorptivity (L mol^{-1} cm^{-1}) ϕ=Quantum yield, Relative brightness = ε* ϕ; τ_{avg}=average lifetime (ns)

Absorbance vs Concentration plot to determine molar absorptivity of QHH

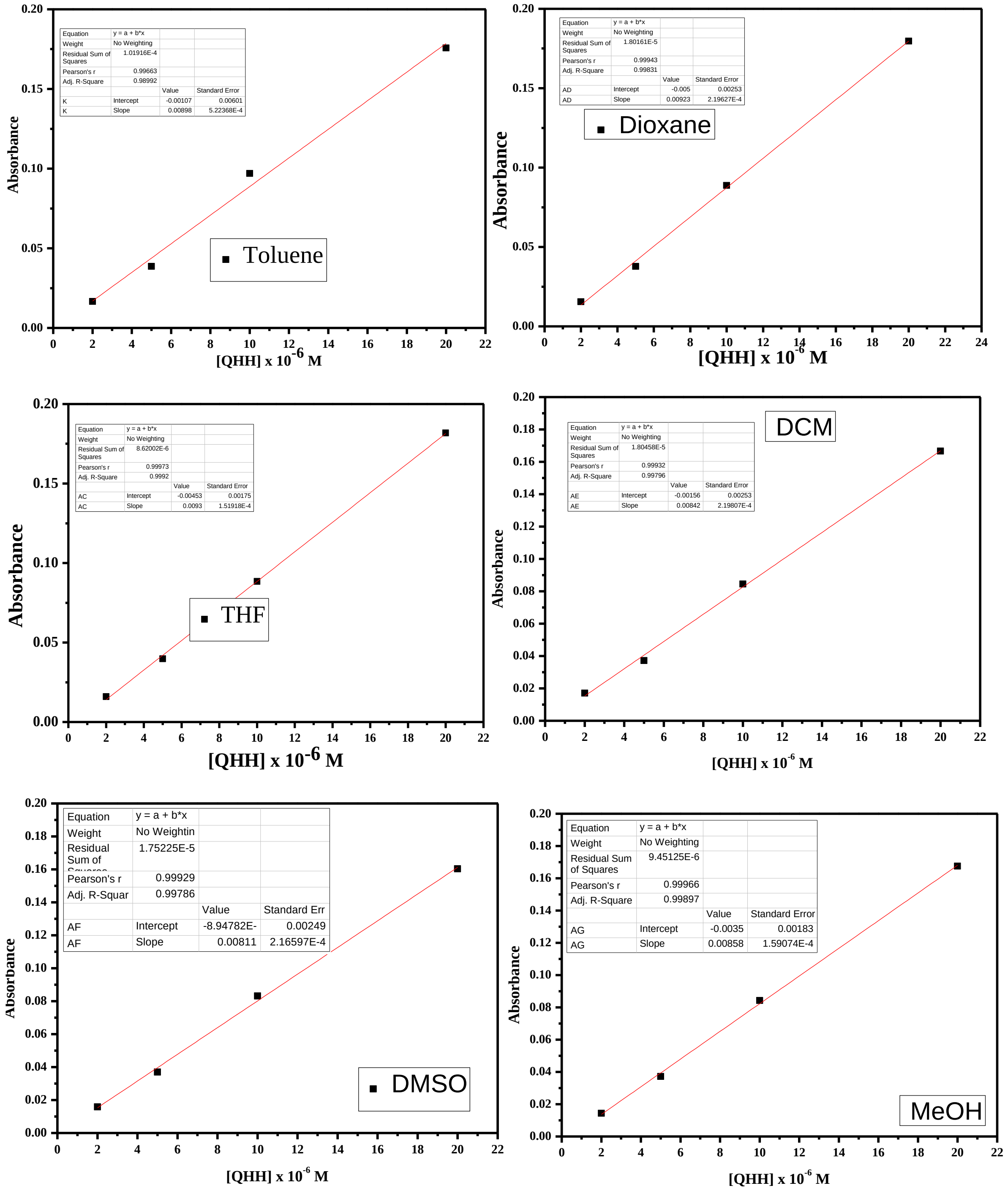

Fig. S23: Molar absorptivity of QHH (2,5,10,20 μM) in different solvents at λ_{abs} **=340nm.**

Absorbance vs Concentration plot to determine molar absorptivity of QPP

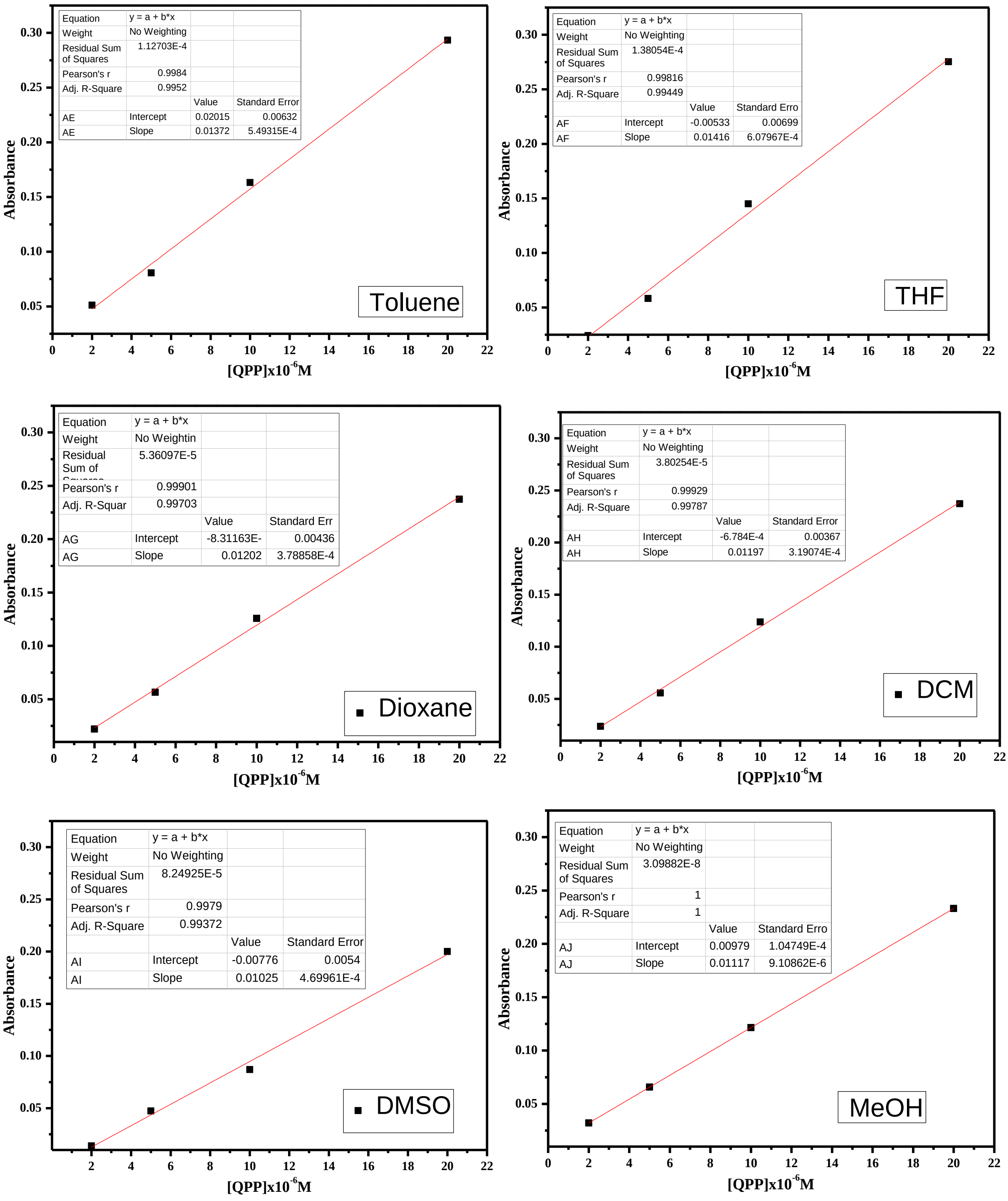

Fig. S24: Molar absorptivity of QPP (2,5,10,20 μM) in different solvents at λ_{abs} **=360nm.**

Absorbance vs Concentration plot to determine molar absorptivity of QPT

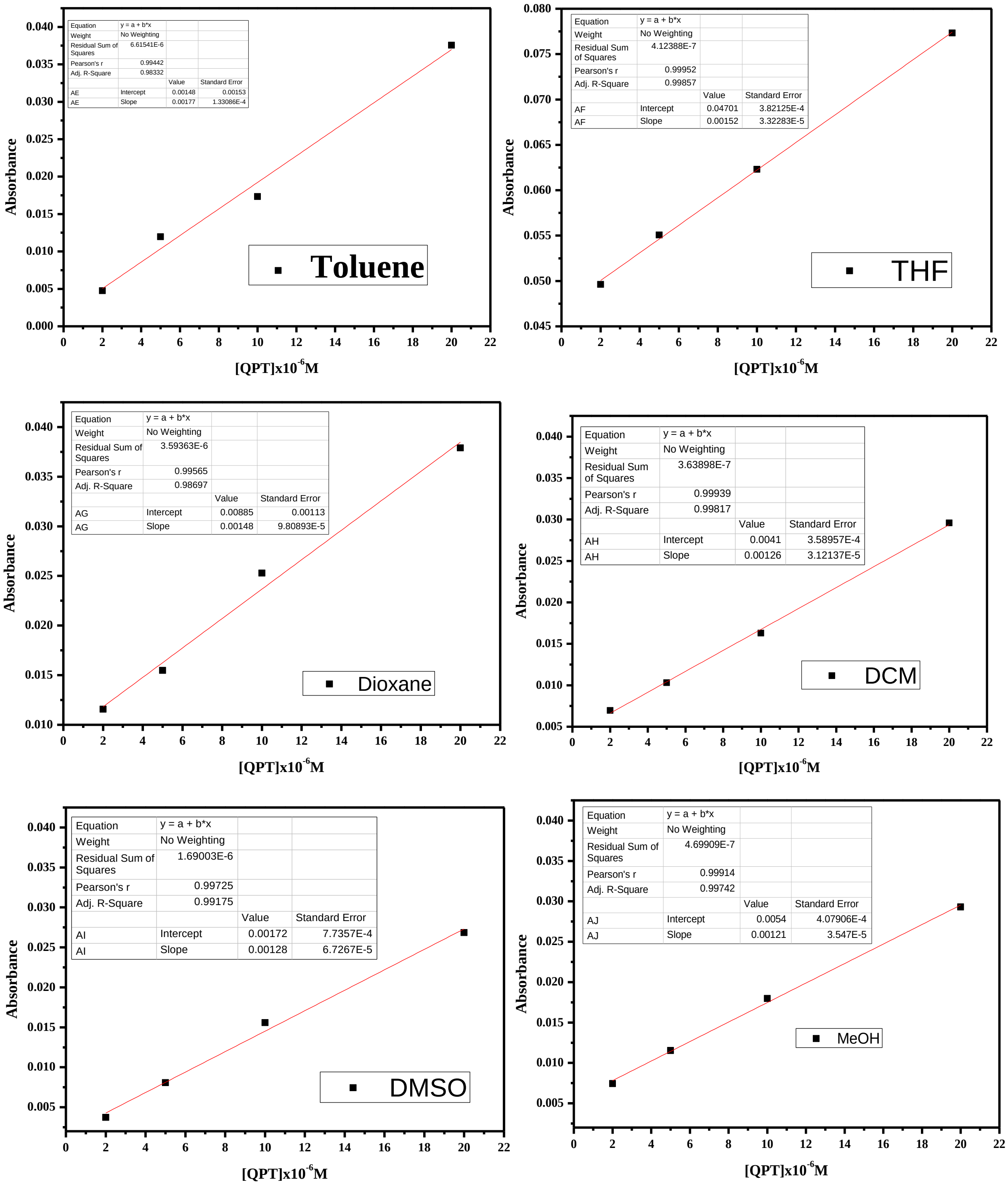

Fig. S25: Molar absorptivity of QPT (2,5,10,20 μM) in different solvents at **λ_{abs} =360nm.**

Absorbance vs Concentration plot to determine molar absorptivity of QTP

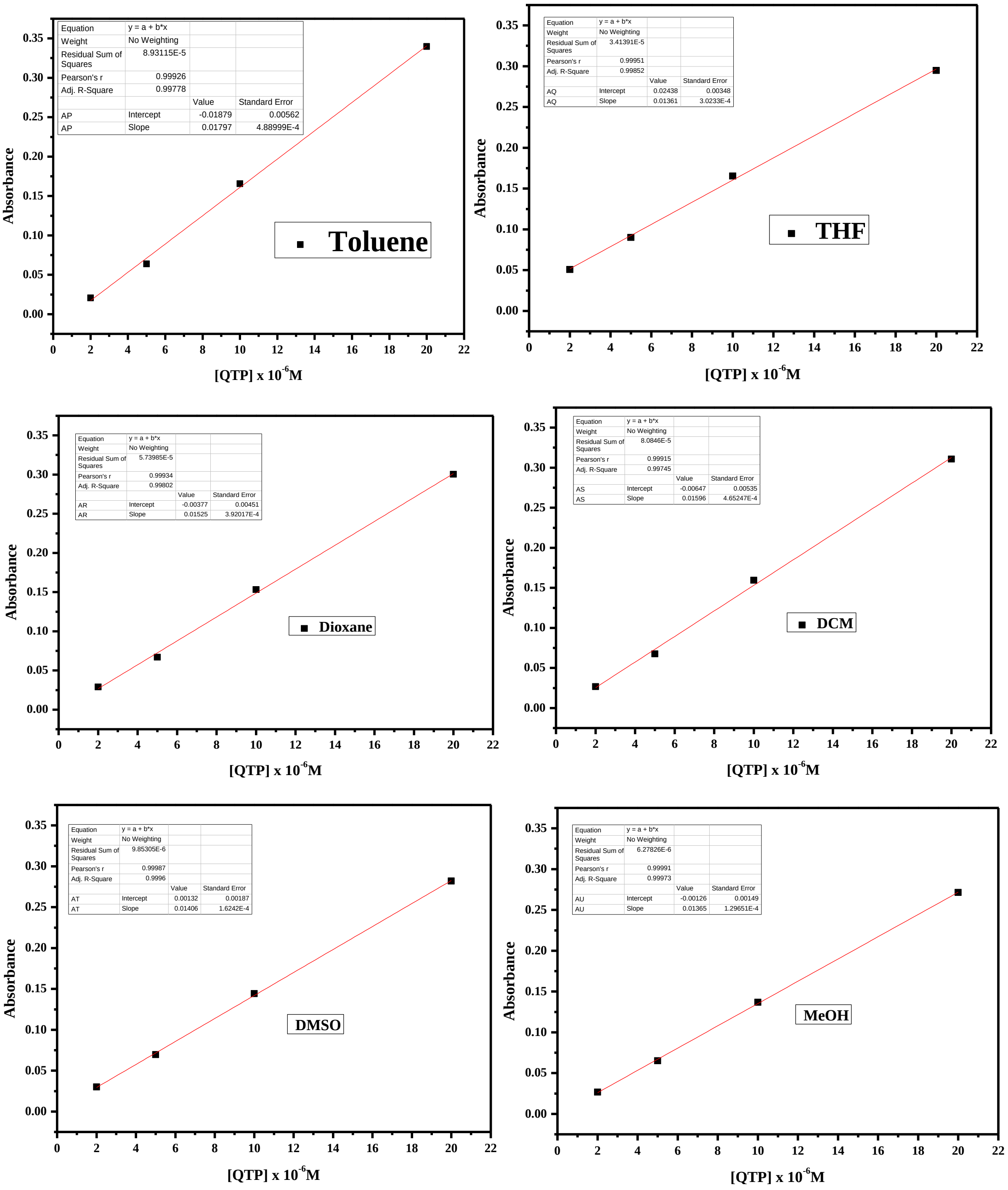

Fig. S26: Molar absorptivity of QTP (2,5,10,20 μM) in different solvents at λ_{abs} **=360nm.**

TCSPC studies for PQNs

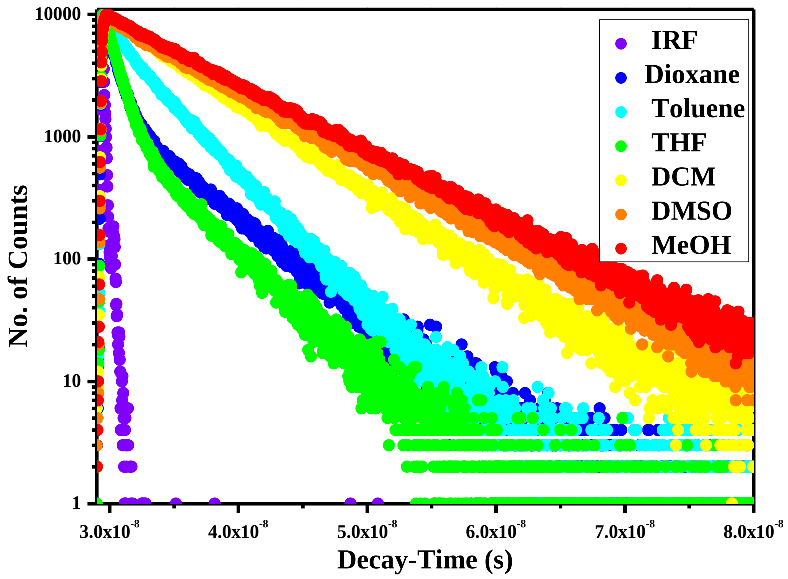

Fig. S27: Life-time decay plot of QHH (10μM) in different solvents.

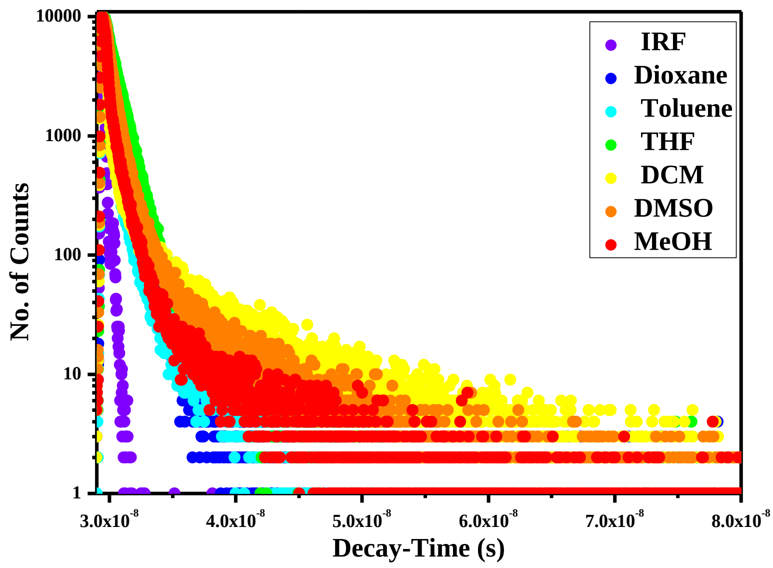

Fig. S28: Life-time decay plot of QPP (10μM) in different solvents.

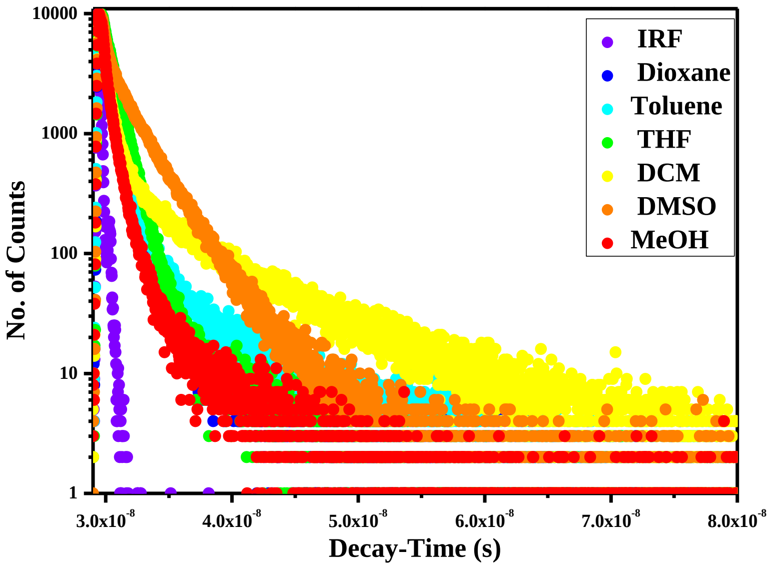

Fig. S29: Life-time decay plot of QPT (10μM) in different solvents.

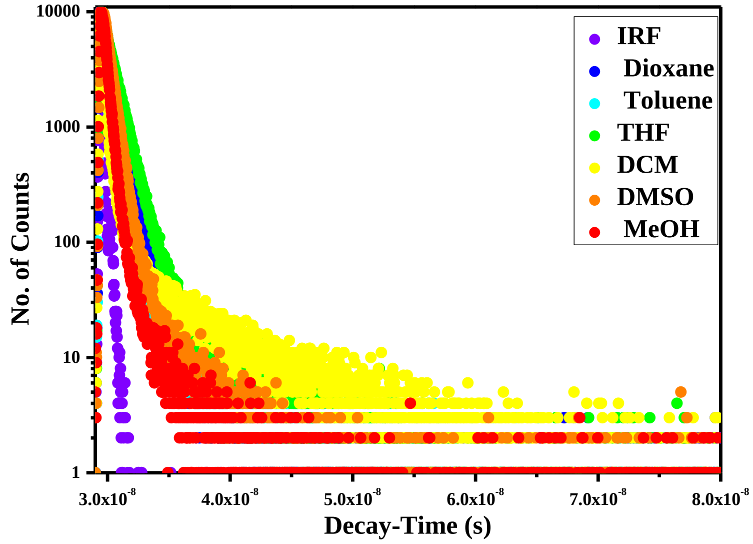

Fig. S30: Life-time decay plot of QTP (10μM) in different solvents.

Table: S3 Lifetime parameter of PQNs.

**Kr/Knr**	QHH	QPP	QPT	QTP
DIOXANE	0.294792	0.014604	0.185629	0.01547
TOLUENE	0.408503	0.005301	0.081699	0.00633
THF	0.577092	0.034673	0.117761	0.026197
DCM	0.794379	0.005575	0.090707	0.005708
MEOH	3.112889	0.040548	0.042933	0.012597
DMSO	2.785625	0.005875	0.24116	0.023043
WATER	85.13755	0.055748	0.115155	0.069555

Table S4: Photophysical parameter of PQNs in solid and solution states

Compound	λ_{em} Solid state (ϕ_f)	λ_{em} Solution state (ϕ_f)	α_{AIE}	K_r/K_{nr} (Solid state)	K_r/K_{nr} (Solution state)
**QHH**	430 nm (0.04)	424 nm (0.51)	0.078	0.0004	0.005138
**QPP**	453 nm (1.19)	459 nm (0.07)	17	0.012043	0.000715
**QPT**	464 nm (1.34)	478 nm (0.17)	7.88	0.014096	0.001666
**QTP**	467 nm (17.97)	481 nm (0.01)	1,797	0.219066	0.000132

Aggregation Induced emission studies

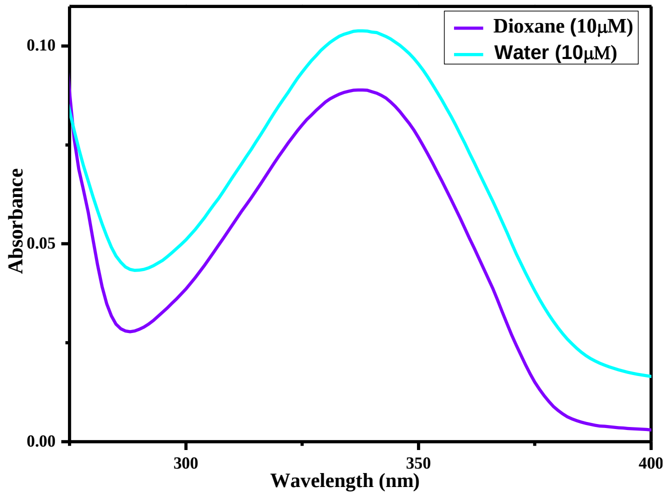

Fig. S31: UV-visible spectra of QHH (10μM) in dioxane and water solvent.

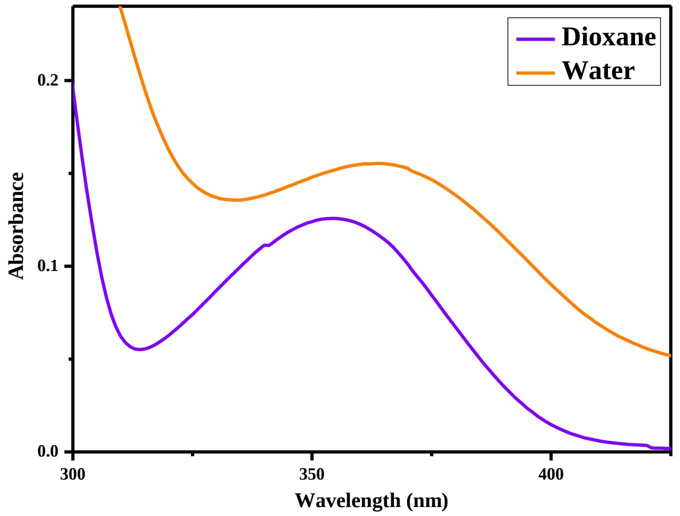

Fig. S32: UV-visible spectra of QPP (10μM) in dioxane and water solvent.

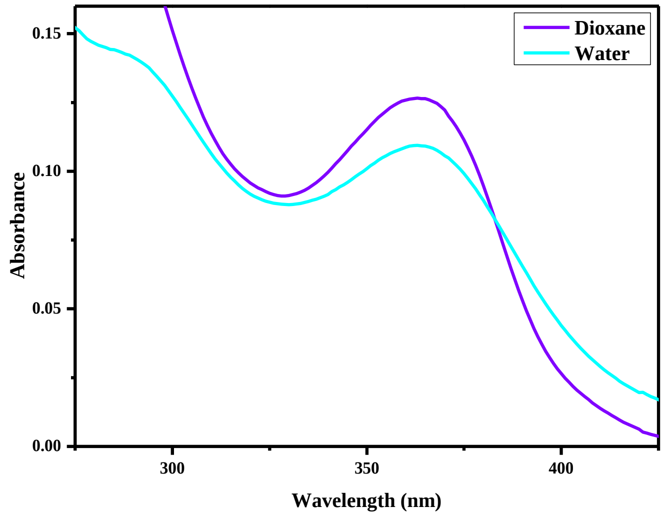

Fig. S33: UV-visible spectra of QPP (10μM) in dioxane and water solvent.

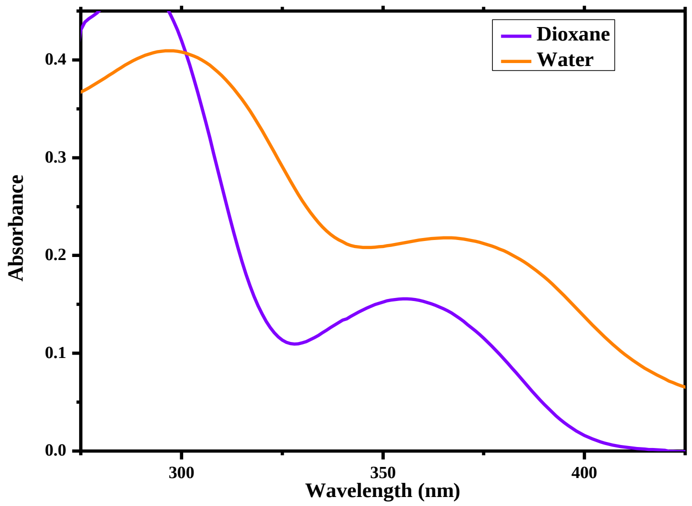

Fig. S34: UV-visible spectra of QTP (10μM) in dioxane and water solvent.

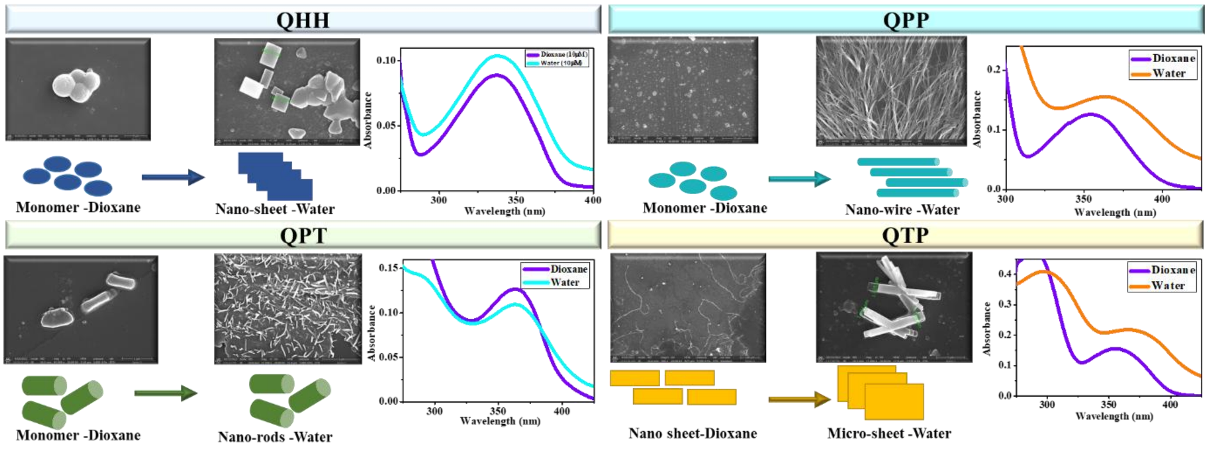

Fig. S35: Detailed analysis of aggregation of formation of PQNs.

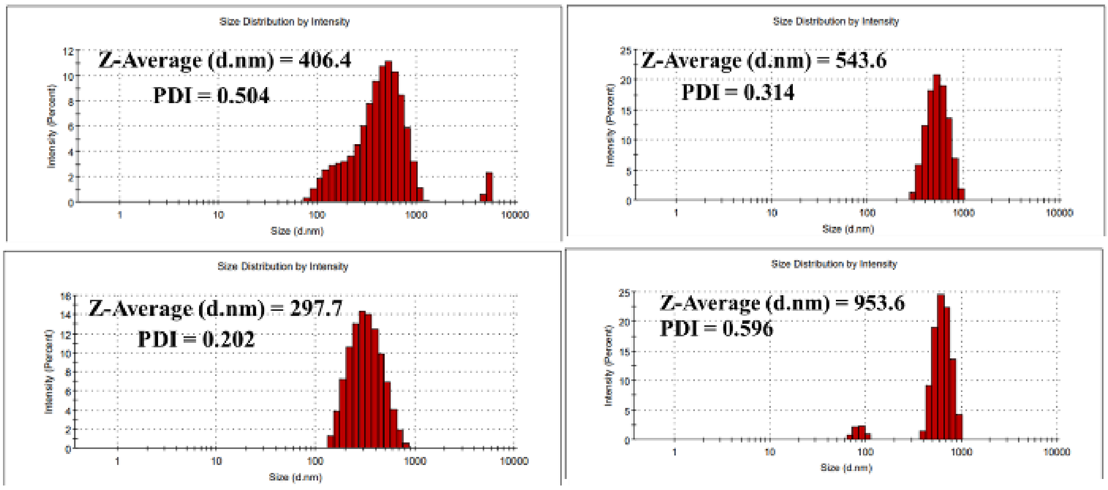

Fig. S36: DLS spectra of PQNs in water at 10μM concentration.

Solid state emission studies and Lifetime of PQNs

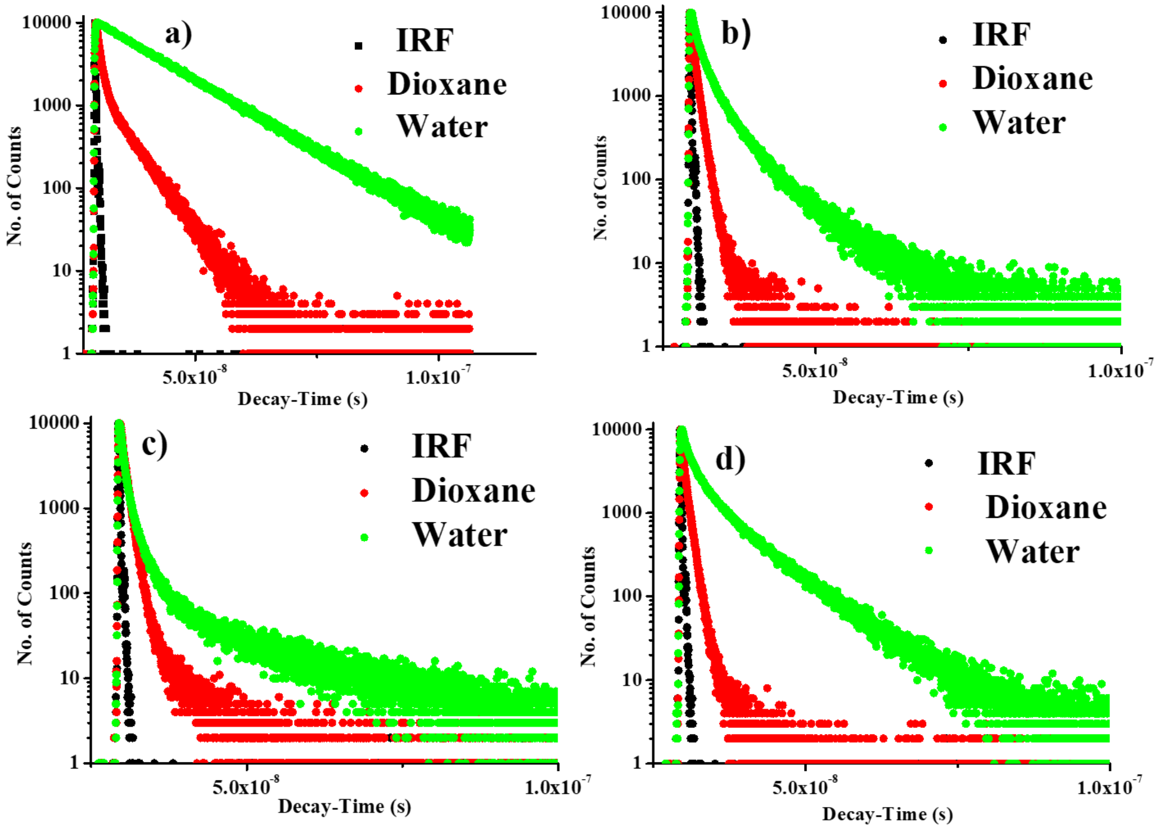

Fig. 37 Fluorescence lifetime decay of (a) QHH, (b) QPP, (c) QPT, and (d) QTP with dioxane and water using λ_{ex}= 375 nm.

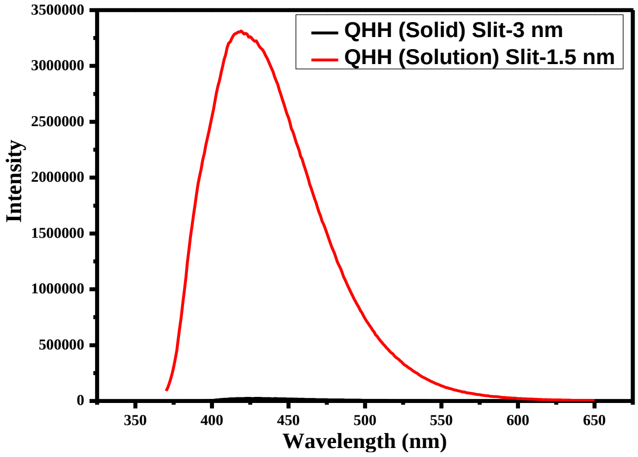

Fig. S38: Fluorescence spectra of QHH (10μM) in solid and solution state using λ_{ex}=360 nm.

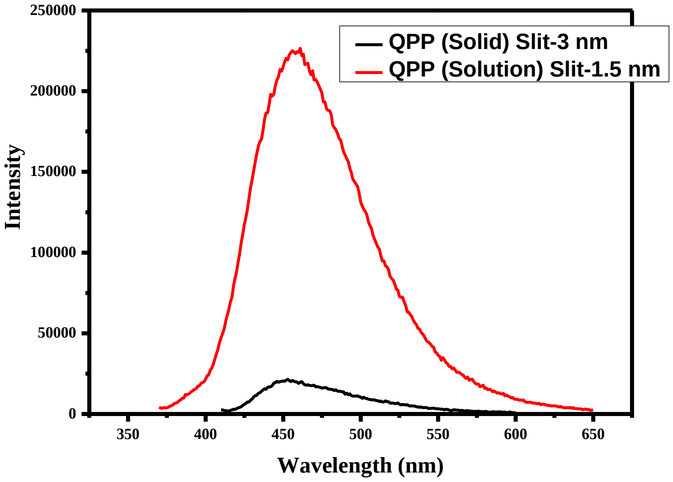

Fig. S39: Fluorescence spectra of QPP (10μM) in solid and solution state using λ_{ex}=360 nm.

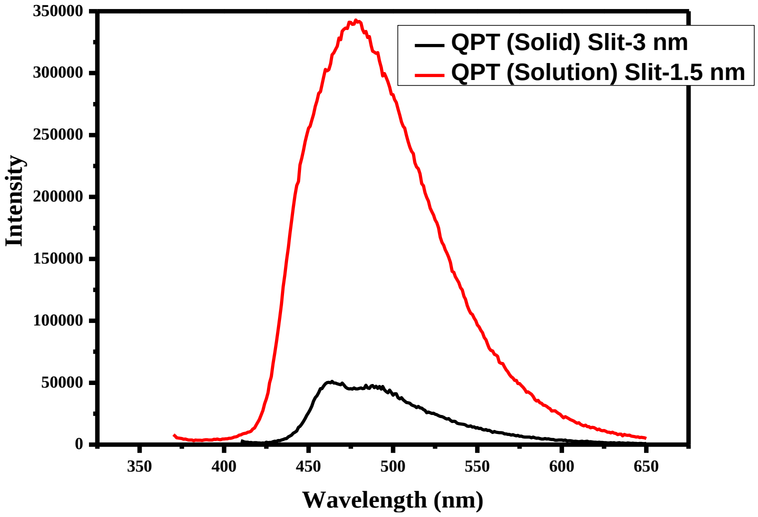

Fig. S40: Fluorescence spectra of QPT (10μM) in solid and solution state using λ_{ex}=360 nm.

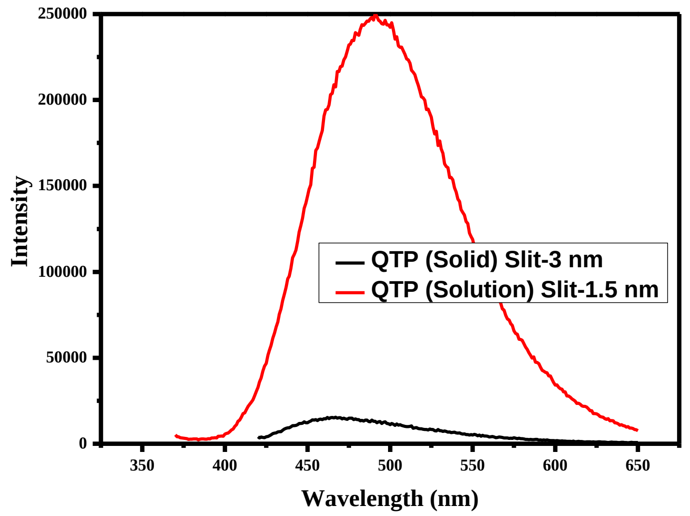

Fig. S41: Fluorescence spectra of QTP (10µM) in solid and solution state using λ_{ex}=360 nm.

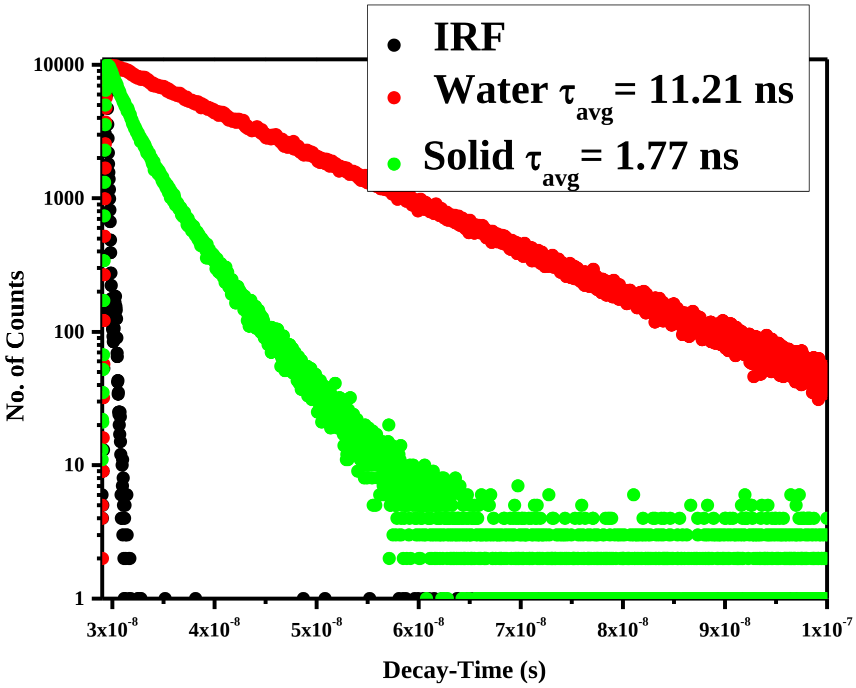

Fig. S42: Lifetime decay plot of QHH (10µM) in solid and solution state using λ_{ex}=375 nm.

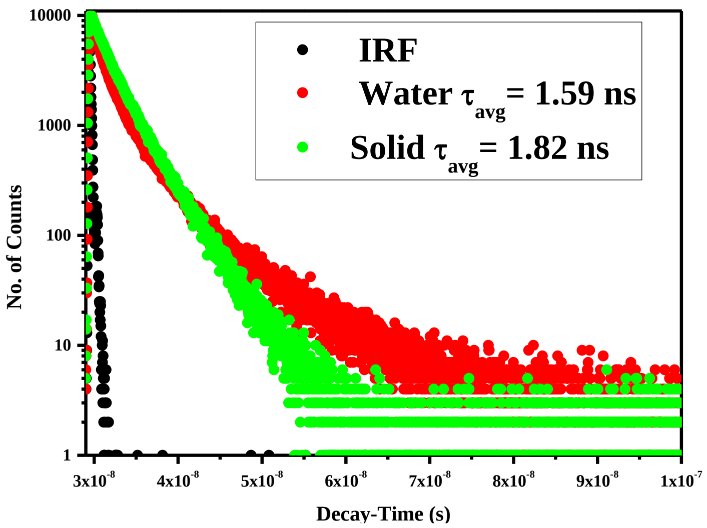

Fig. S43: Lifetime decay plot of QPP (10μM) in solid and solution state using λ_{ex}=375 nm.

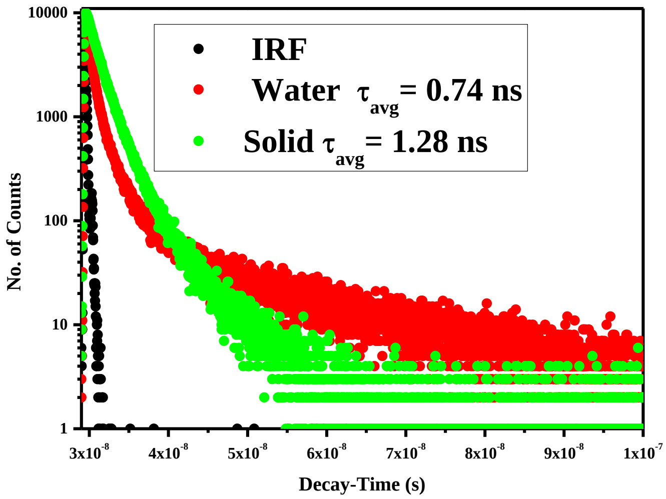

Fig. S44: Lifetime decay plot of QPT (10μM) in solid and solution state using λ_{ex}=375 nm.

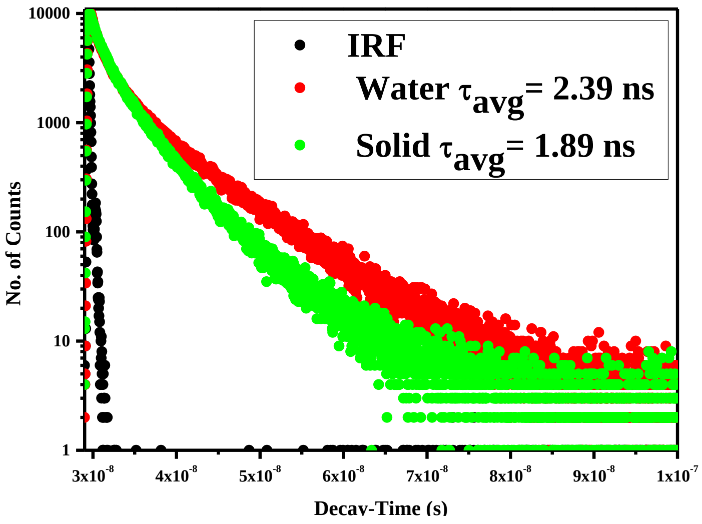

Fig. S45: Lifetime decay plot of QTP (10μM) in solid and solution state using λ_{ex}=375 nm.

Crystal structure analysis of QTP

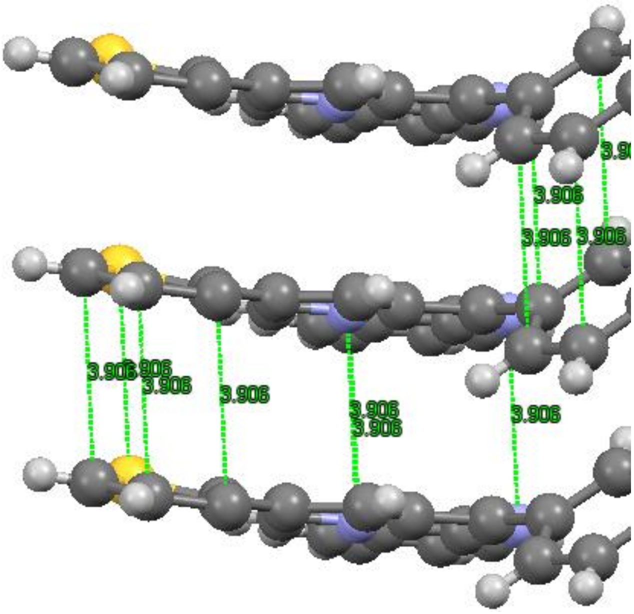

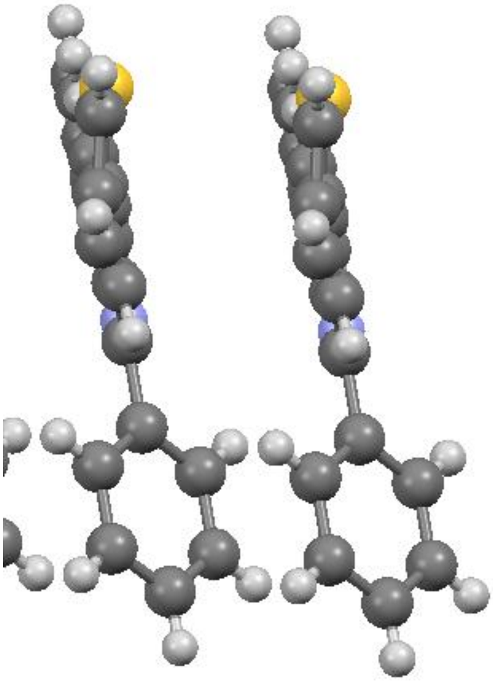

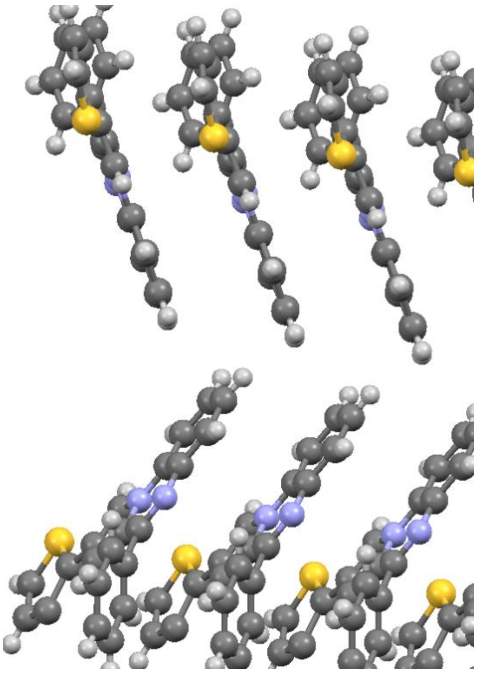

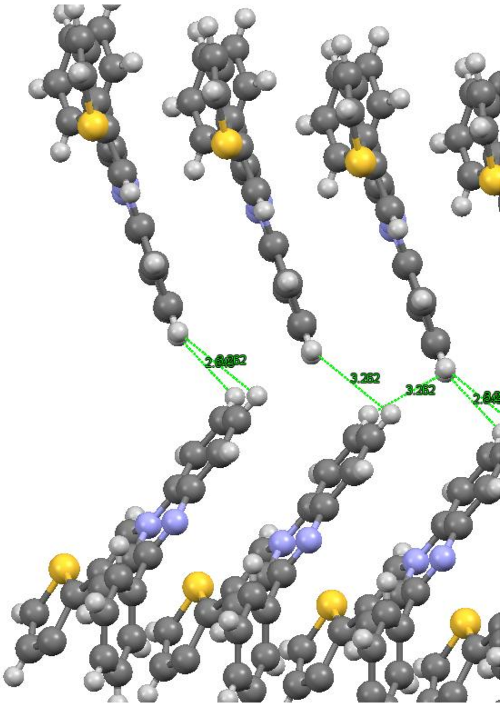

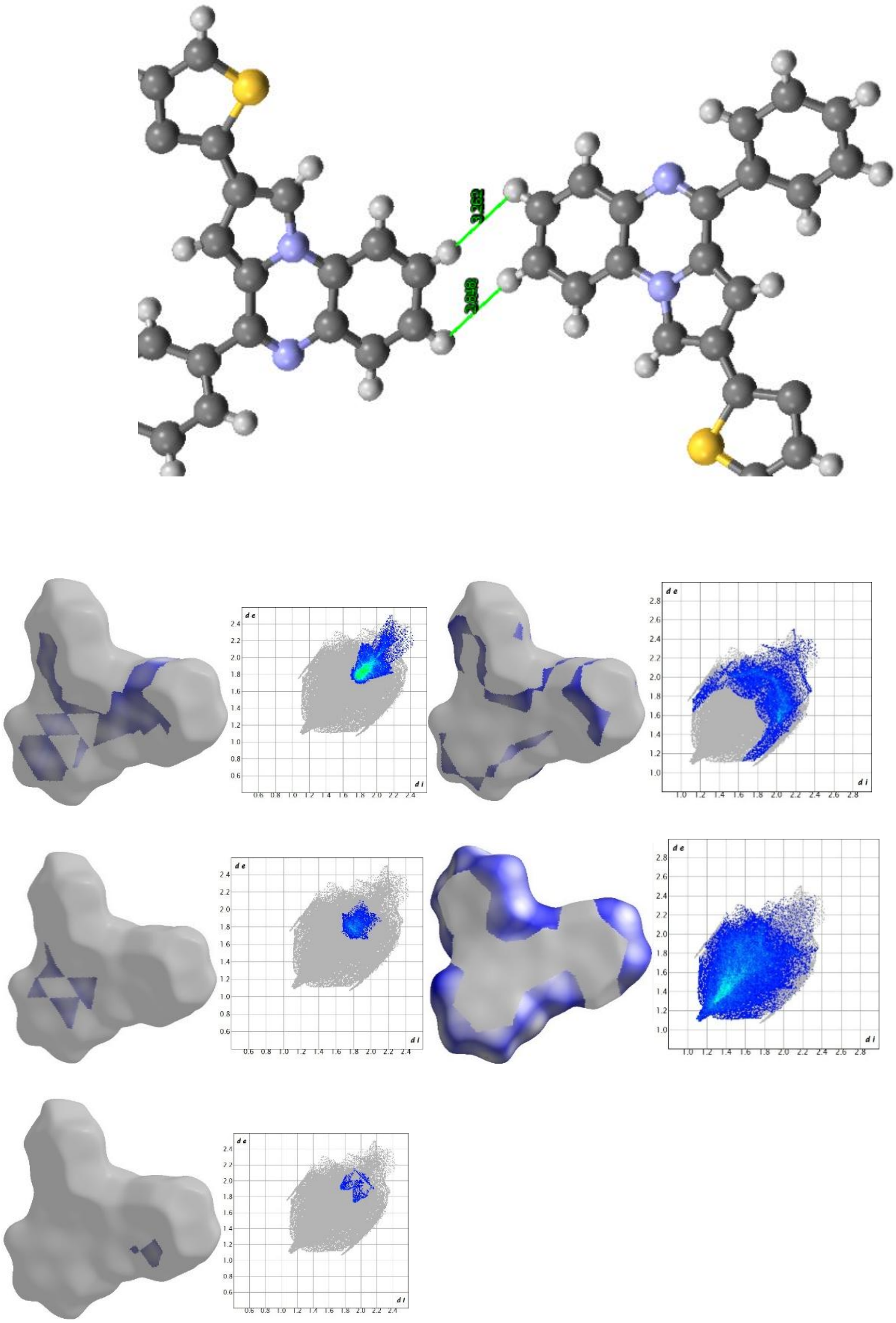

Fig. S46: QTP-Crystal packing modes, Hirshfeld Surface analysis of QTP crystal for weak interaction quantification**.**

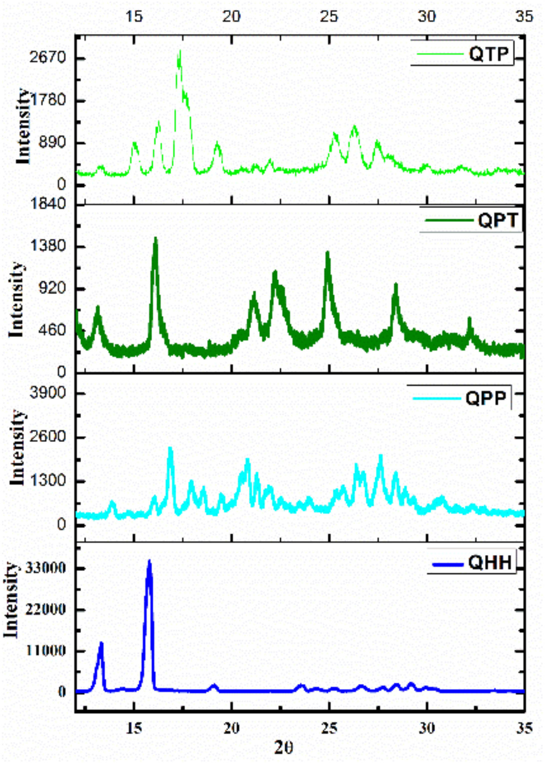

Fig. S47: PXRD spectra of PQNs.

Emission and Lifetime of PQNs with varying pH

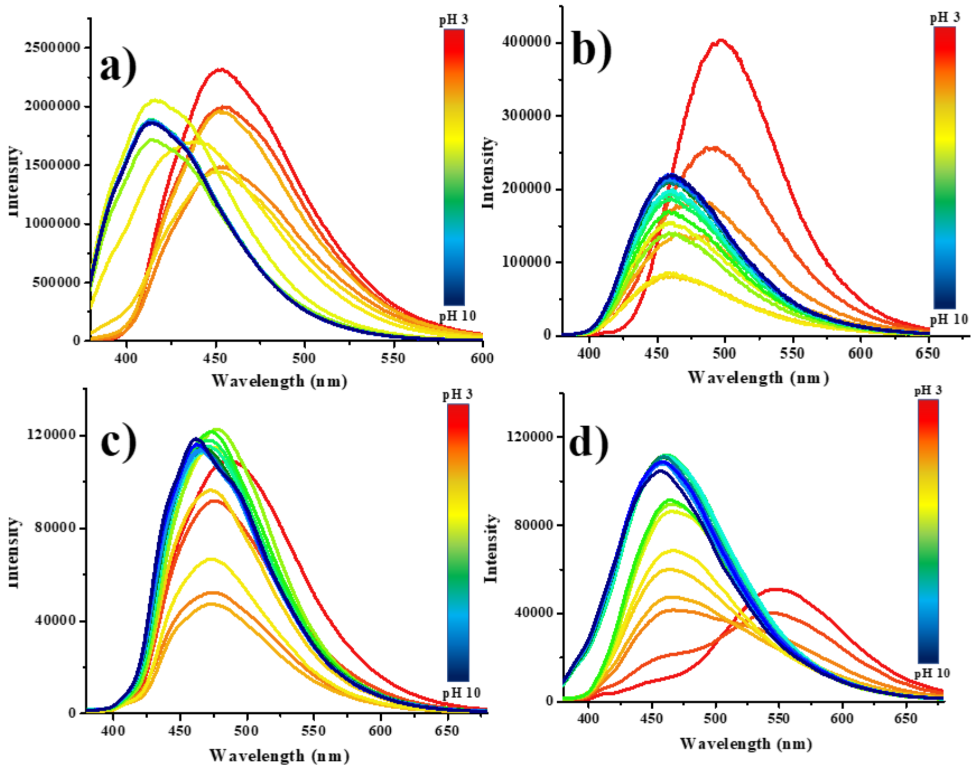

Figure S48: a) Fluorescence spectra of (a) QHH, (b) QPP, (c) QPT, and (d) QTP in different pH.

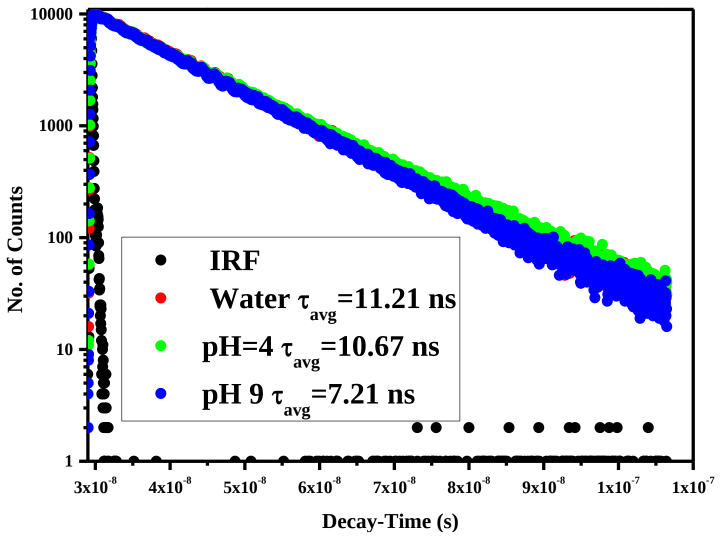

Fig. S49: Lifetime decay plot of QHH (10μM) in different pH.

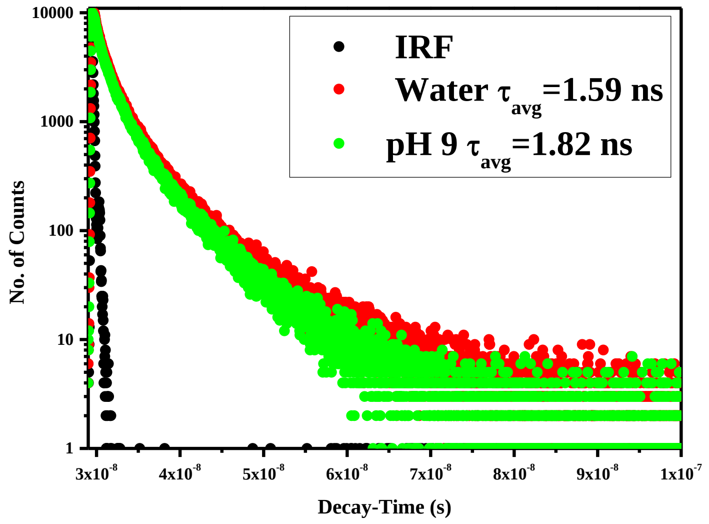

Fig. S50: Lifetime decay spectra of QPP (10μM) in different pH.

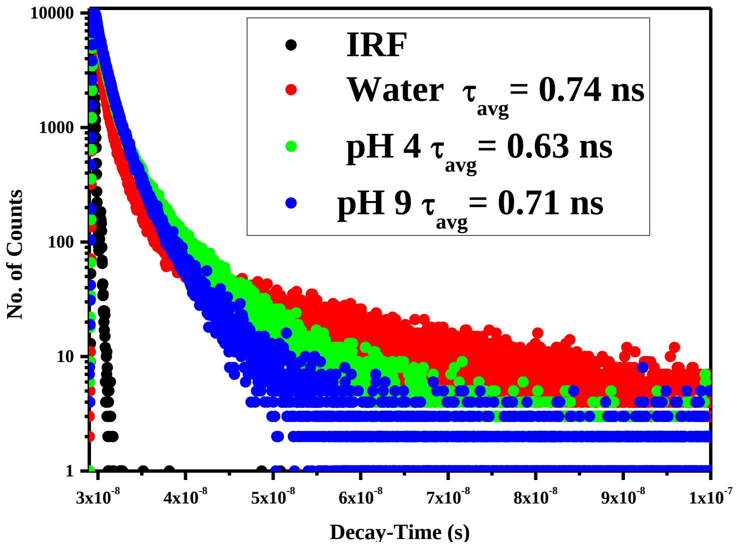

Fig. S51: Lifetime decay plot of QPT (10μM) in different pH.

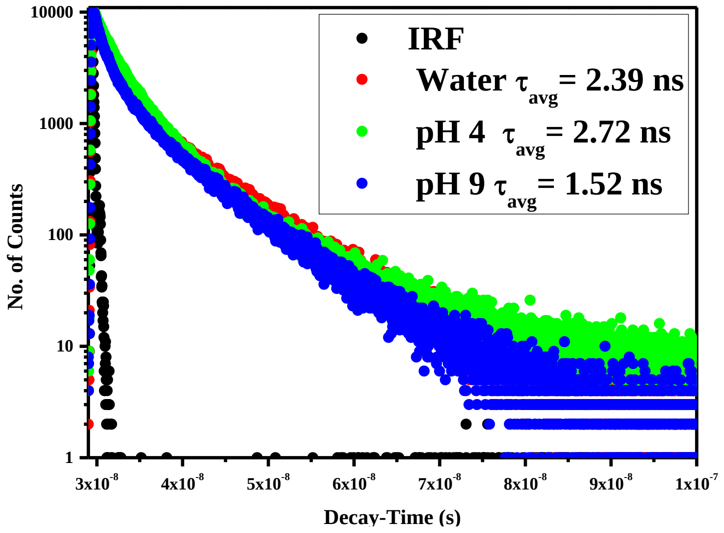

Fig. S52: Lifetime decay plot of QTP (10μM) in different pH.

MTT Assay

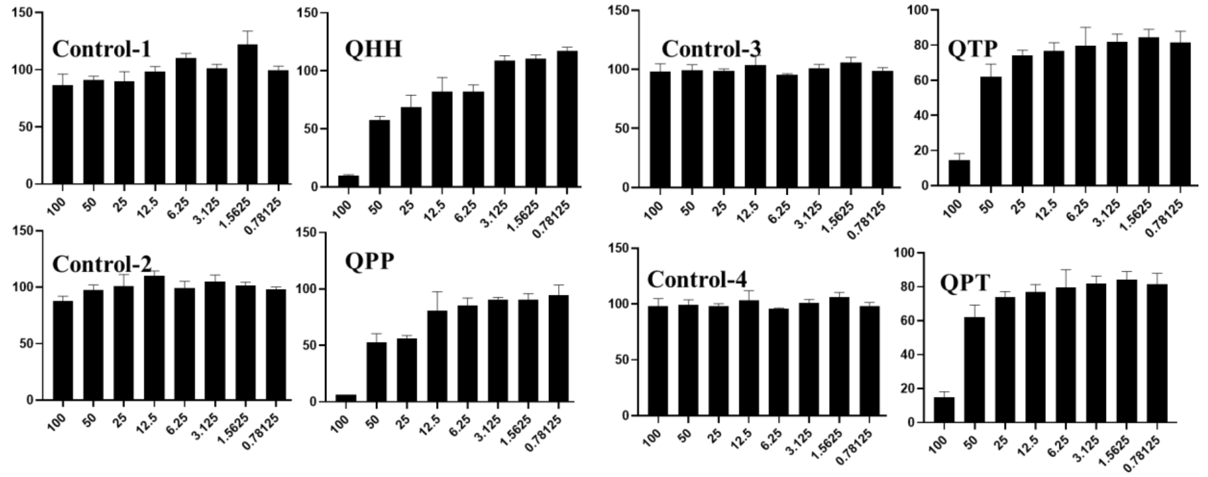

Figure S53: MTT data of PQNs in RAW 264.7 cells [control-1 for QHH, control-2 for QPP, control-3 for QTP, control-4 for QPT].

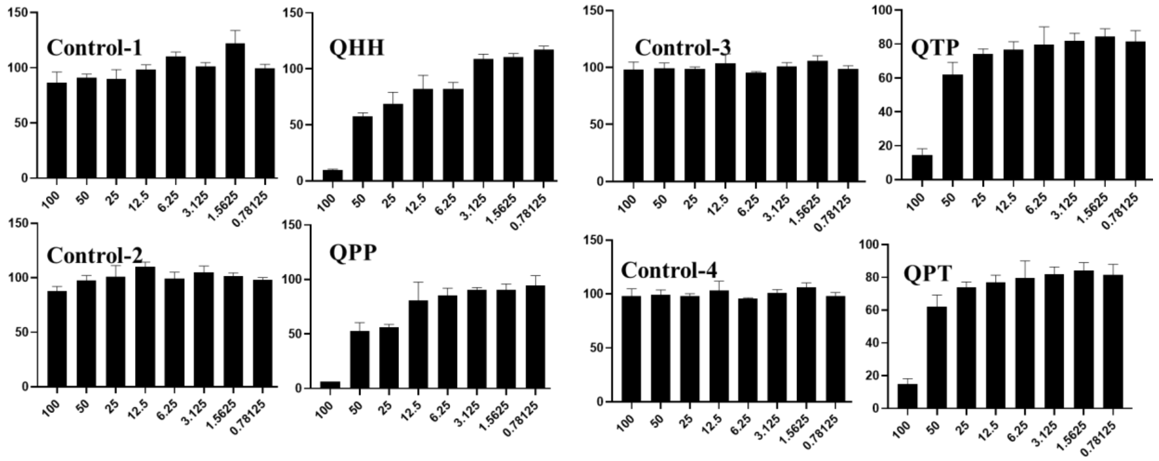

Figure S54: MTT data of PQNs in CAL 20 cells [control-1 for QHH, control-2 for QPP, control-3 for QTP, control-4 for QPT].

Confocal Imaging

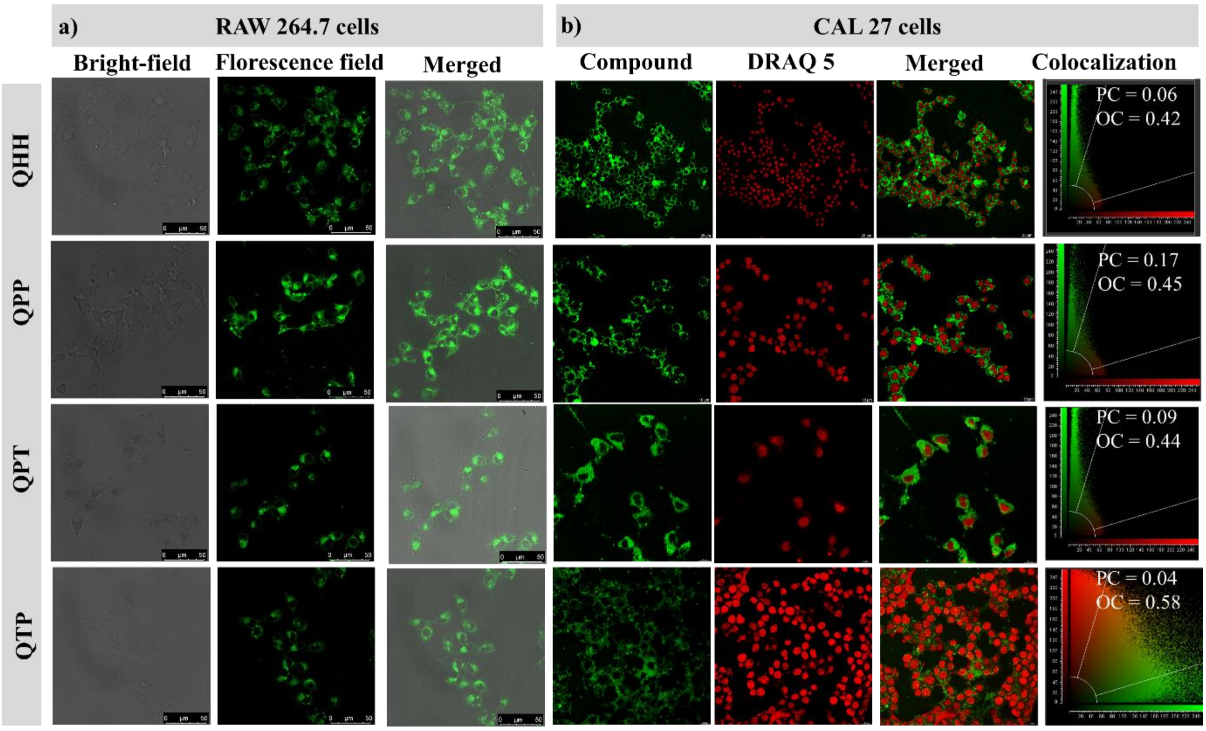

Fig. S55 (a) CLSM imaging of PQNs (10 µM, λ_{ex}= 405 nm, λ_{em}= 415-550 nm) in RAW 264.7 cells and (b) CAL 27 cells incubated with PQNs (10 µM, λ_{ex}= 405 nm, λ_{em}= 415-550 nm)and DRAQ5 (5 µM, nucleus tracker, λ_{ex} = 633 nm, λ_{em} = 650–700 nm). Scale bar=50µm.

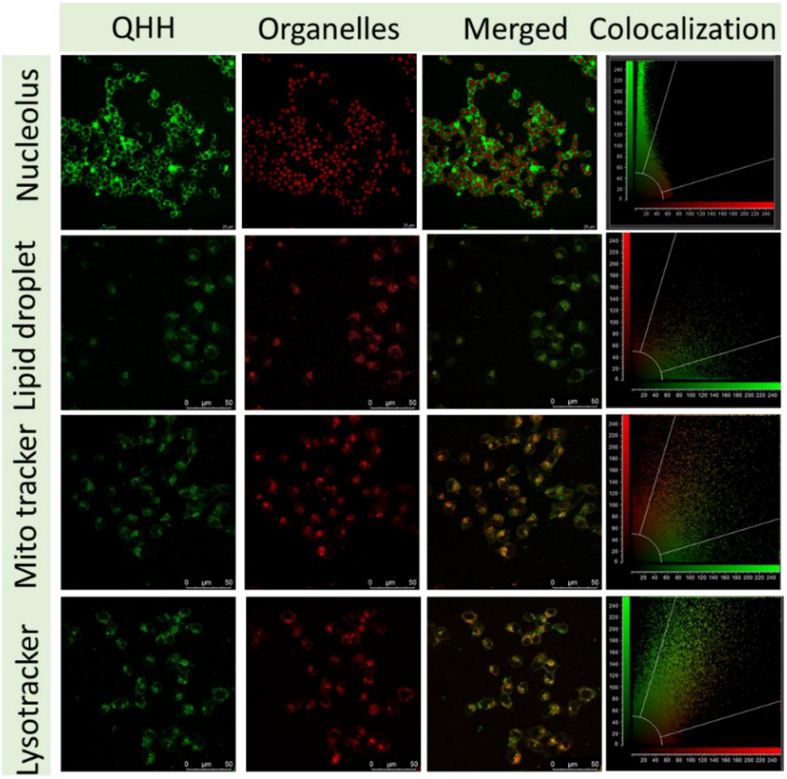

Fig. S56: Confocal imaging comparison of QHH (10µM) with different organelle staining biomarkers.

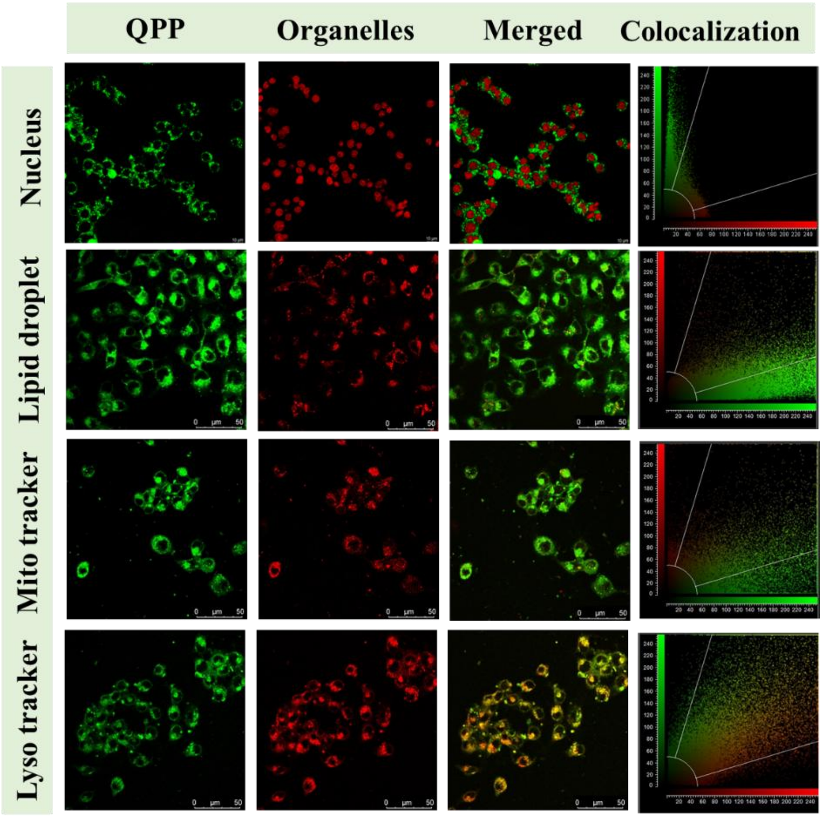

Fig. S57: Confocal imaging comparison of QPP (10μM) with different organelle staining biomarkers.

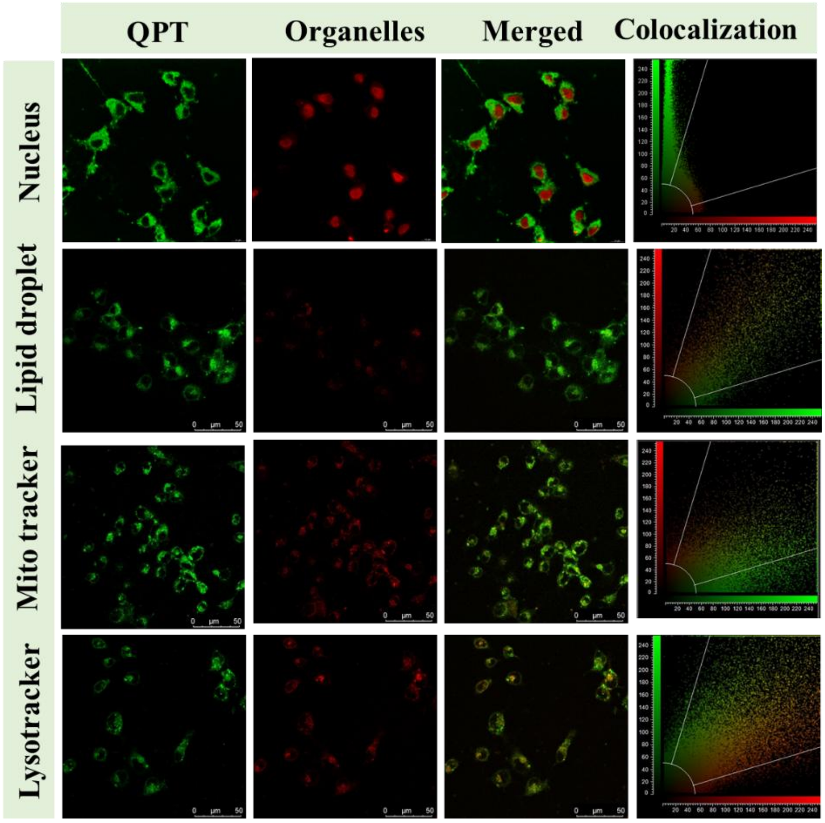

Fig. S58: Confocal imaging comparison of QPT (10μM) with different organelle staining biomarkers.

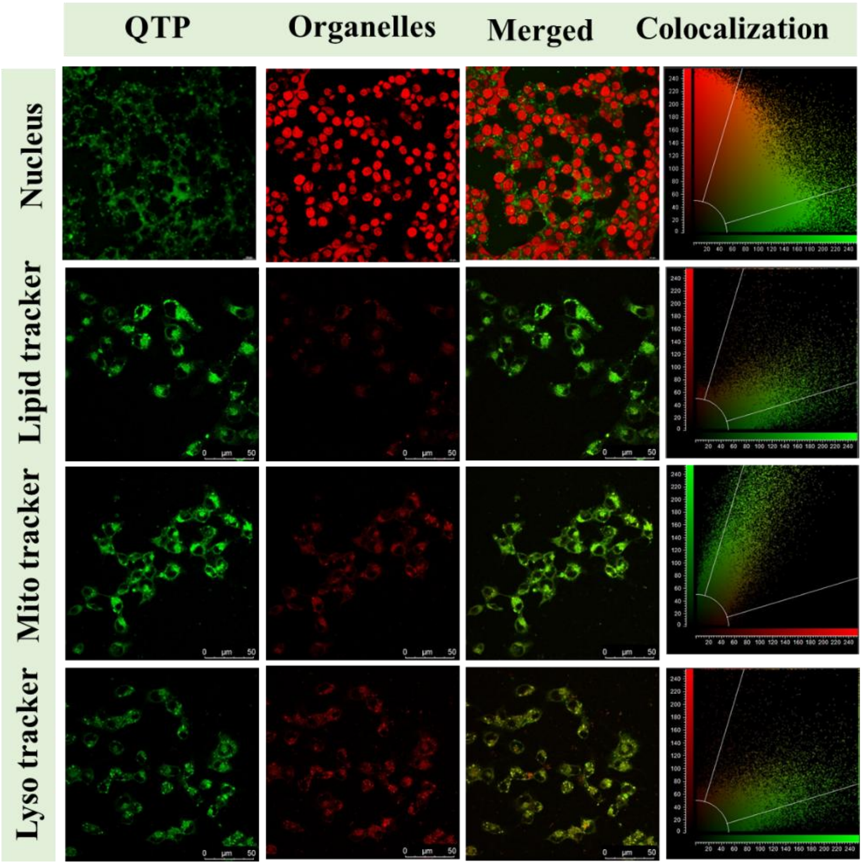

Fig. S59: Confocal imaging comparison of QTP (10µM) with different organelle staining biomarkers.

FLIM:

After completing the preliminary confocal experiments, we used the FLIM technique on QHH and QTP to monitor the effect of polarity changes in RAW cells. These molecules were chosen based on their TCSPC results, where significant changes were noticed in the average lifetimes upon changing the polarity, and pH. LPS induction was used to trigger cell inflammation, as it is well known to affect the cellular microenvironment. The average lifetime (τ_{avg}) of whole cells was found to be 2.6 ns and 4.95 ns for QHH and QTP, respectively. Upon LPS induction, a change in the average fluorescence lifetime was observed, between 2.8 - 5.3 ns for QTP and 2.03 - 5.66 ns for QHH (Table: xx). Subsequently, region of interest (ROI)-based image analysis was carried out for pre- and post-induction images. Herein, three regions were examined: cytoplasm, near cell membrane, and inside the nucleus (Table: xx). For cells containing QHH and without induction, lifetime was found to be 2.53 ns in the cytoplasm, 2.92 ns near the cell membrane, and 2.99 ns inside the nucleus. On the other hand, under inflammation conditions, the lifetimes were increased and were found to be 3.01 ns in the cytoplasm, 3.16 ns near the cell membrane, and 3.23 ns inside the nucleus. Upon performing the ROI image analysis for QTP-containing cells, two different lifetimes were mainly observed. In cells without induction, lifetimes were 3.56 ns and 4.0 ns, whereas LPS-induced cells showed lifetimes of 3.23 ns and 3.83 ns. FLIM experiments clearly show the sensitivity of the probes to the changes in the cellular microenvironment, especially the changes in polarity and pH.

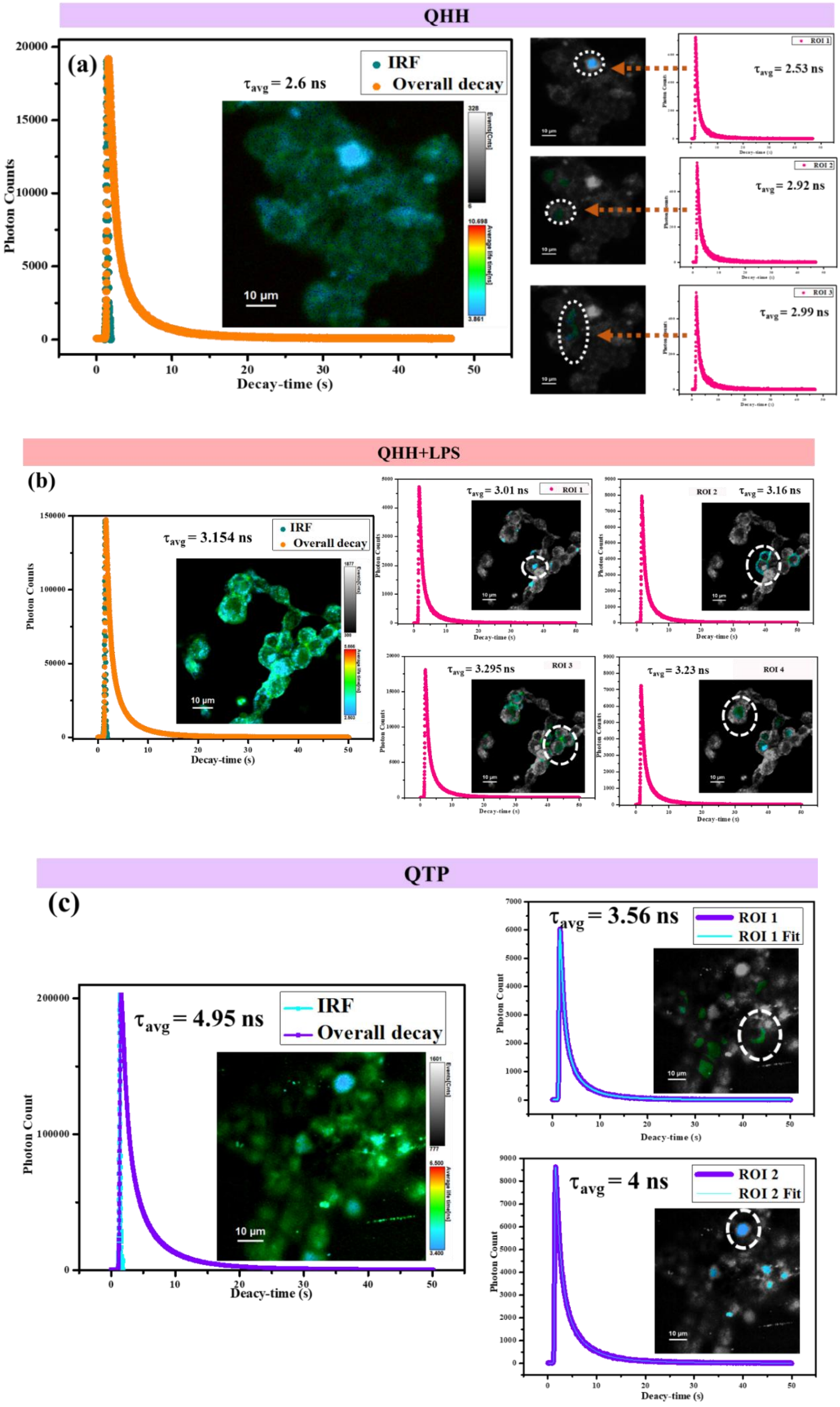

QHH
(a)
IRF
Overall decay
τavg = 2.6 ns
Photon Counts
Decay-time (s)
10 μm
τavg = 2.53 ns
τavg = 2.92 ns
τavg = 2.99 ns
ROI 1
ROI 2
ROI 3
QHH+LPS
(b)
τavg = 3.154 ns
IRF
Overall decay
Photon Counts
Decay-time (s)
τavg = 3.01 ns
τavg = 3.16 ns
τavg = 3.295 ns
τavg = 3.23 ns
ROI 4
QTP
(c)
τavg = 4.95 ns
IRF
Overall decay
Photon Count
Deacy-time (s)
τavg = 3.56 ns
ROI 1 Fit
τavg = 4 ns
ROI 2 Fit

QTP+LPS

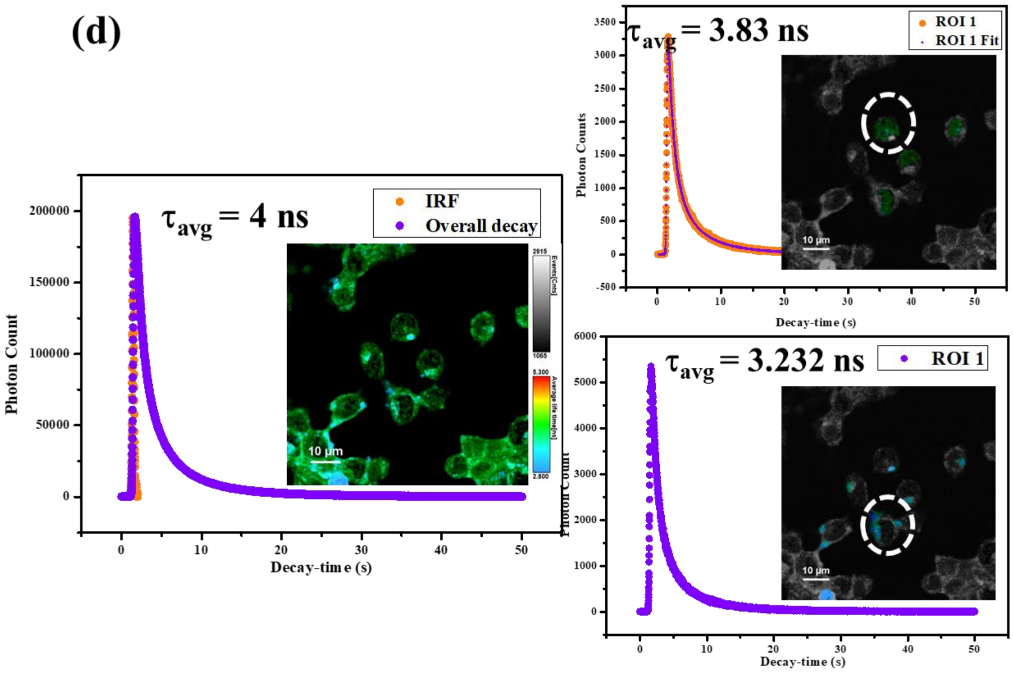

Fig. S60: ROI-based image analysis a) FLIM imaging of QHH (10μM) in RAW cell, b) FLIM imaging of QHH (10μM) under inflammation condition in RAW cell, c) FLIM imaging of QTP (10μM) in RAW cell, FLIM imaging of QTP (10μM) under inflammation condition in RAW cell.

Computational Studies

Table S5a. TDDFT-predicted absorption peak positions in dioxane and water

Compound	Transition	Dioxane λ_{abs} (oscillator strength) [dominating excitation]	Water λ_{abs} (oscillator strength) [dominating excitation]
**QHH**	$S_0 \rightarrow S_1$	331 (0.261) [HOMO→LUMO],	330 (0.233) [HOMO→LUMO],
	$S_0 \rightarrow S_2$	299 (0.015) [HOMO–1→LUMO]	299 (0.016) [HOMO–1→LUMO]
**QPP**	$S_0 \rightarrow S_1$	358 (0.275) [HOMO→LUMO],	368 (0.495) [HOMO→LUMO],
	$S_0 \rightarrow S_2$	343 (0.130) [HOMO–1→LUMO]	351 (0.246) [HOMO–1→LUMO]
**QPT**	$S_0 \rightarrow S_1$	378 (0.301) [HOMO→LUMO],	378 (0.245) [HOMO→LUMO],
	$S_0 \rightarrow S_2$	360 (0.198) [HOMO–1→LUMO]	365 (0.195) [HOMO–1→LUMO]
**QTP**	$S_0 \rightarrow S_1$	361 (0.143) [HOMO→LUMO],	364 (0.140) [HOMO→LUMO],
	$S_0 \rightarrow S_2$	356 (0.255) [HOMO–1→LUMO]	353 (0.204) [HOMO–1→LUMO]

Table S5b. TDDFT-predicted emission peak positions in dioxane and water

**Compounds**	**Emission from S_1 (of monomer) λ_{em} (oscillator strength)**		**Emission from S_1 (of dimer) λ_{em} (oscillator strength)**
	**Dioxane**	**Water**	**Water**
**QHH ($S_1 \rightarrow S_0$)**	399 (0.2432)	419 (0.4523)	503 (0.0065)
**QPP ($S_1 \rightarrow S_0$)**	475 (0.2550)	492 (0.4627)	485 (0.3953)[1]<br>488 (0.4347)[2]
**QPT ($S_1 \rightarrow S_0$)**	479 (0.2734)	474 (0.2601)	536 (0.0004)
**QTP ($S_1' \rightarrow S_0$)**	440 (0.2081)	456 (0.3605)[a]	450 (0.1521)[a]
**QTP ($S_1 \rightarrow S_0$)**	-	492 (0.4667)[b]	483 (0.4172)[b]

[a] emission originating from S_1, [b] emission originating from S_1' of QTP, [1,2] emission originating from two different S_1 dimer geometries of QPP

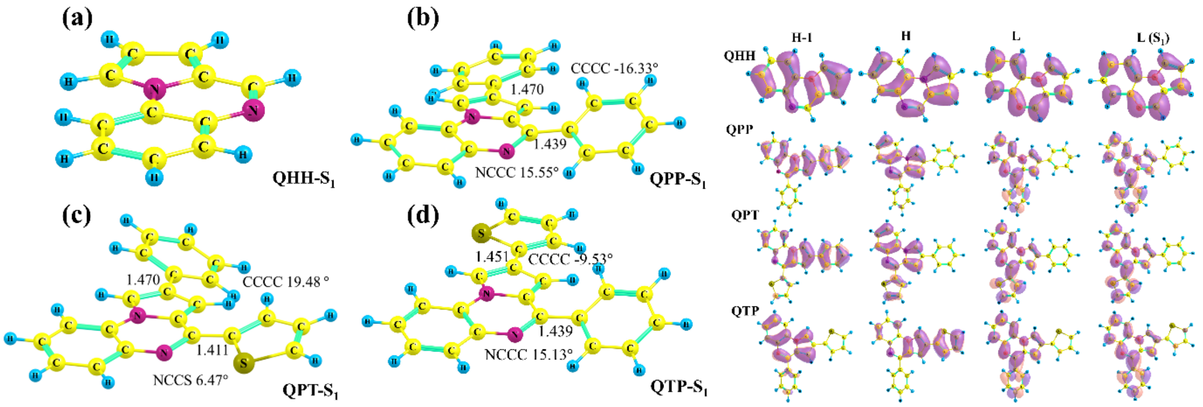

Fig. S61 Fluorescent S_1 minima geometries with charge distributions (orbitals of S_0 and S_1) of (a) QHH (b) QPP (c) QPT (d) QTP

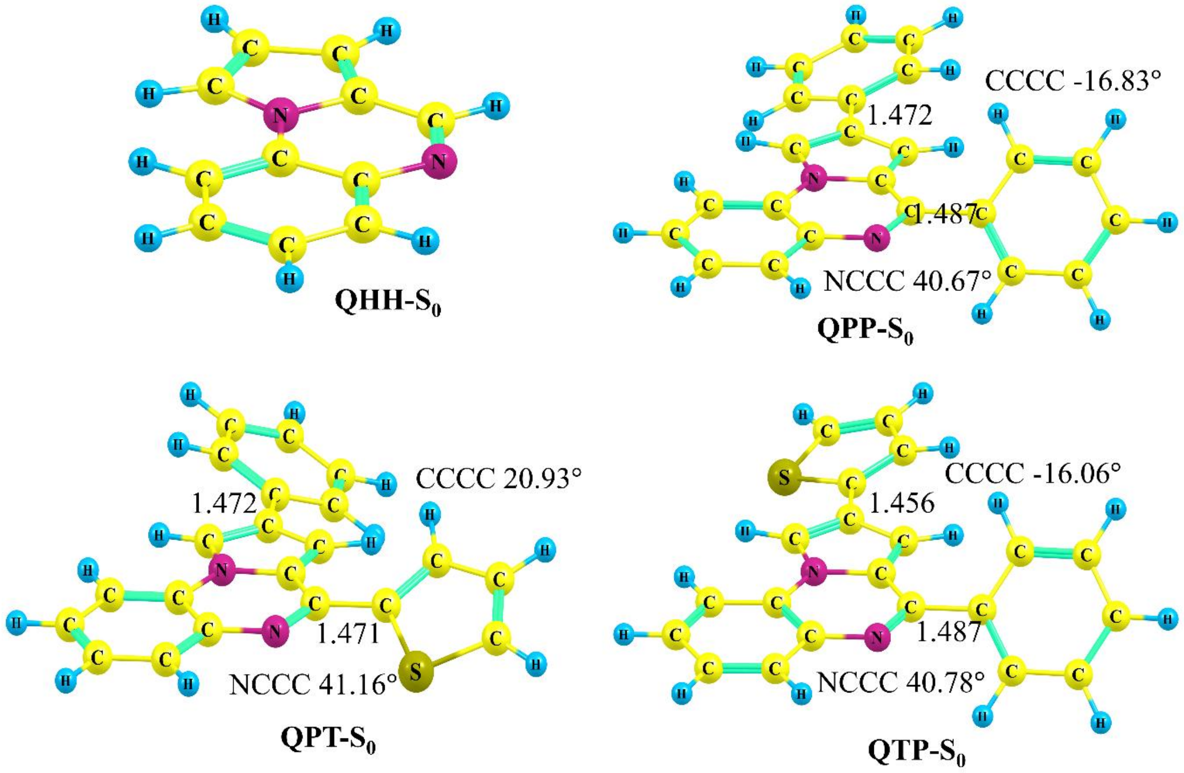

Fig. S62: S_0 minima geometries of QHH, QPP, QPT, and QTP.

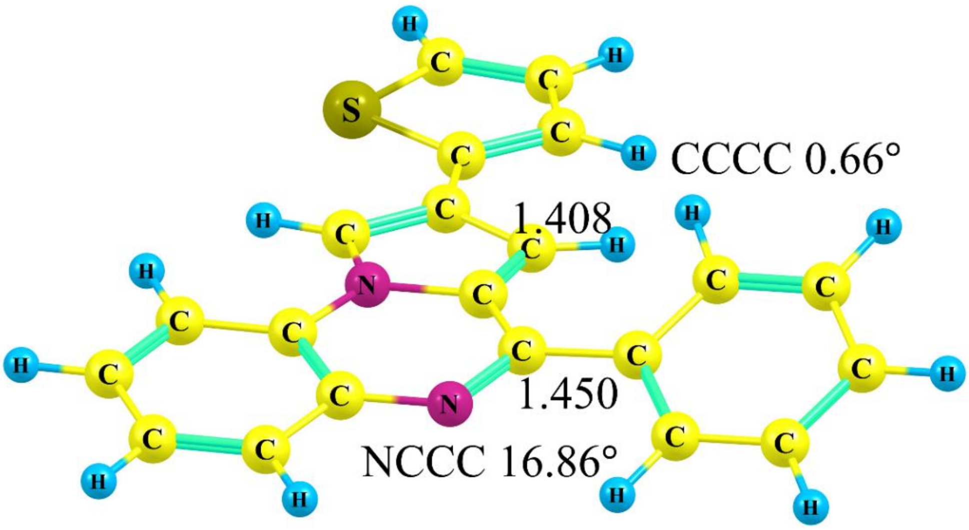

QTP-S$_1'$

Fig. S63: S_1 minima geometry of QTP- S_1'.

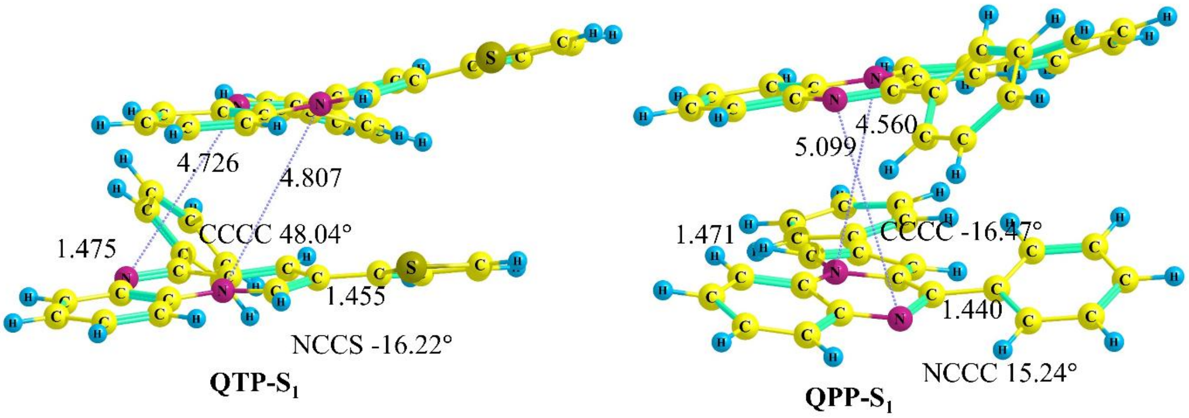

Fig. S64: Structures of the optimized S_1 of π-stacked dimers of QPP and QTP.

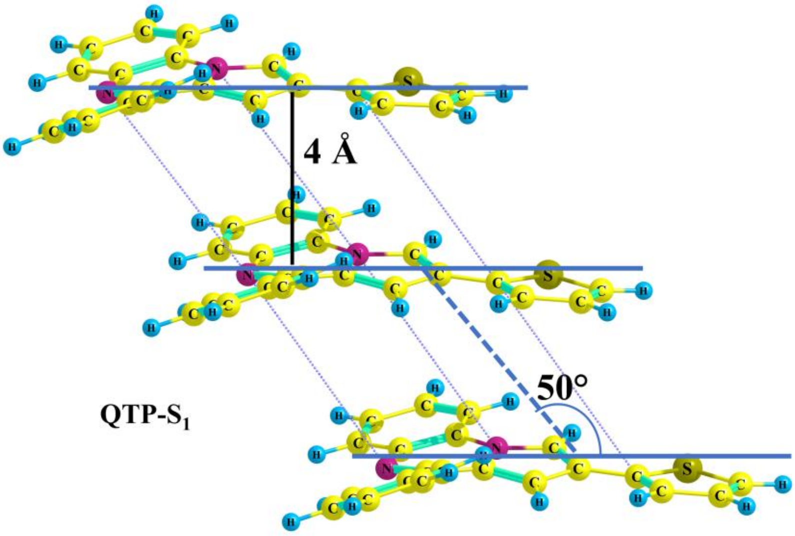

Fig. S65: slip-stacked trimer of QTP-S_1.

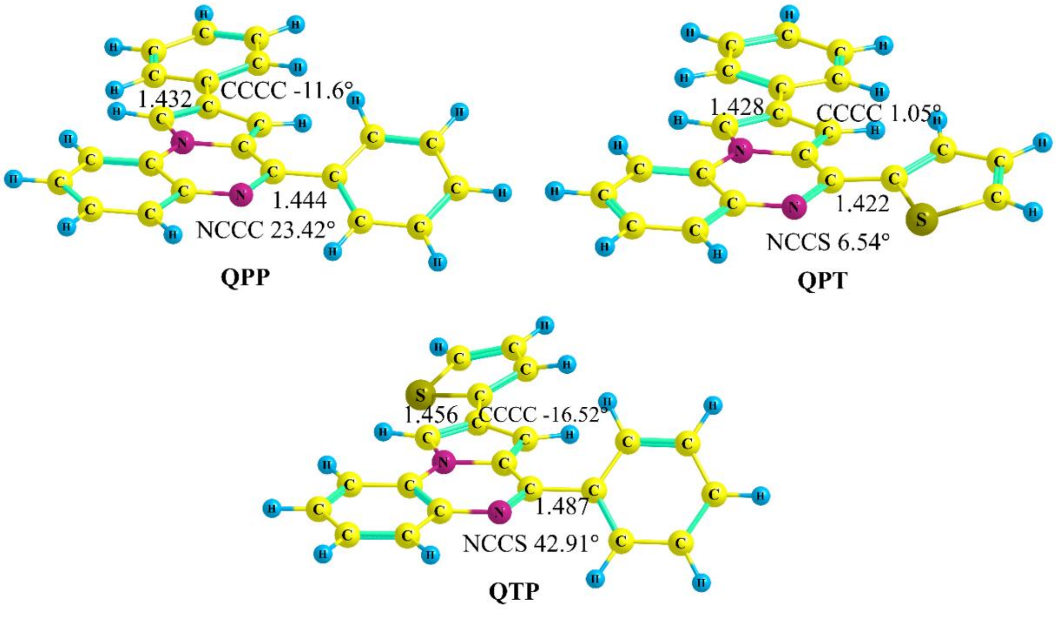

Fig. S66: The S2\T3\T4 degenerate geometries of QPP (top left), QPT (top right), and QTP (bottom).

Table S6: Spin-Orbit Coupling (SOC) parameters (in cm^{-1}) for S_0 and S_1 optimized geometries of QHH, QPP, QPT, and QTP.

SOC parameters (in cm^{-1}) At the below-mentioned optimized geometries	**S_1-T_1**	**S_1 -T_2**	**S_1-T_3**	**S_2-T_1**	**S_2 -T_2**	**S_2-T_3**	**S_2-T_4**
**QHH-S_0 Min**	0.163	0.895	0.160	1.125	0.296	0.189	0.369
**QHH-S_1 Min**	0.127	0.761	0.326	1.063	0.217	0.078	4.617
**QPP-S_0 Min**	0.229	0.544	0.415	0.712	0.180	0.203	0.300
**QPP-S_1 Min**	0.816	0.818	1.263	0.720	0.148	0.199	0.199
**QPT-S_0 Min**	0.404	0.878	1.803	0.913	0.114	0.799	0.441
**QPT-S_1 Min**	0.201	1.177	1.209	0.686	0.232	0.098	0.089
**QTP-S_0 Min**	0.853	0.279	0.157	0.384	0.483	0.531	0.700
**QTP-S_1 Min**	0.496	0.140	0.176	0.577	0.537	0.238	0.845

Table S7: a) TDDFT-predicted phosphorescence peak positions (in water) in nm **b) T_2(min) /T_3(min) – T_1 energy gap in eV c)** $S_{1\text{-min}}$ – $T_{1/2/3\text{-min}}$ gap in eV **d)** Emission Dimer (optimized) with oscillator strengths (in water) in nm **e)** Emission of slip-stack dimer and trimer of QTP-S1 **f)** Dipole moments (Debye).

**a) Theoretical Phosphorescence peak positions (in water) in nm**		
**QHH**	T_2 (min) → S_0	445
**QPP**	T_2 (min) → S_0	493
**QPT**	T_2 (min) → S_0	505
**QTP**	T_3 (min) → S_0	476

**b) T_2(min) /T_3(min) – T_1 energy gap in eV**	
	**T_2 (min)– T_1**
**QPP-T_2**	0.2449
**QPT-T_2**	0.5383
**QHH-T_2**	0.4278
	**T_3 (min)-T_2**
QTP-T_3	0.2076

**c) S_1-(min) – T_1(min)/T_2(min)/T_3(min) energy gap in eV**		
	**S_1 (min)– T_1(min)**	**S_1 (min)– T_2 (min)**

**QHH**	1.273	0.132
**QPP**	0.919	0.111
**QPT**	1.084	0.602
		**S_1 (min)– T_3 (min)**
**QTP**	1.001	-0.125

**d) Emission of dimer (at S_1 optimized geometry) with oscillator strengths (in water) in nm**	
**QHH-S_1**	503 (0.0065)
**QPP-S_1**	485 (0.3953)
**QPT-S_1**	536 (0.0004)
**QTP-S_1'**	450 (0.1521)
**QTP-S_1**	483 (0.4172)

**e) Emission of slip-stack dimer and trimer of QTP-S_1**		
	**Dimer**	**Trimer**
**QTP-S_1**	484 (0.0403), 483.66 (0.8461)	484 (0.0468), 484 (0.0045), 483.75 (1.2590)

**f) Dipole moment (Debye)**	**S_0-min geometry**	**S_1-min geometry**
**QHH**	3.45	6.18
**QPP**	4.91	7.32
**QPT**	3.08	7.36
**QTP**	4.04	6.67 (S_1)

Phosphorescence

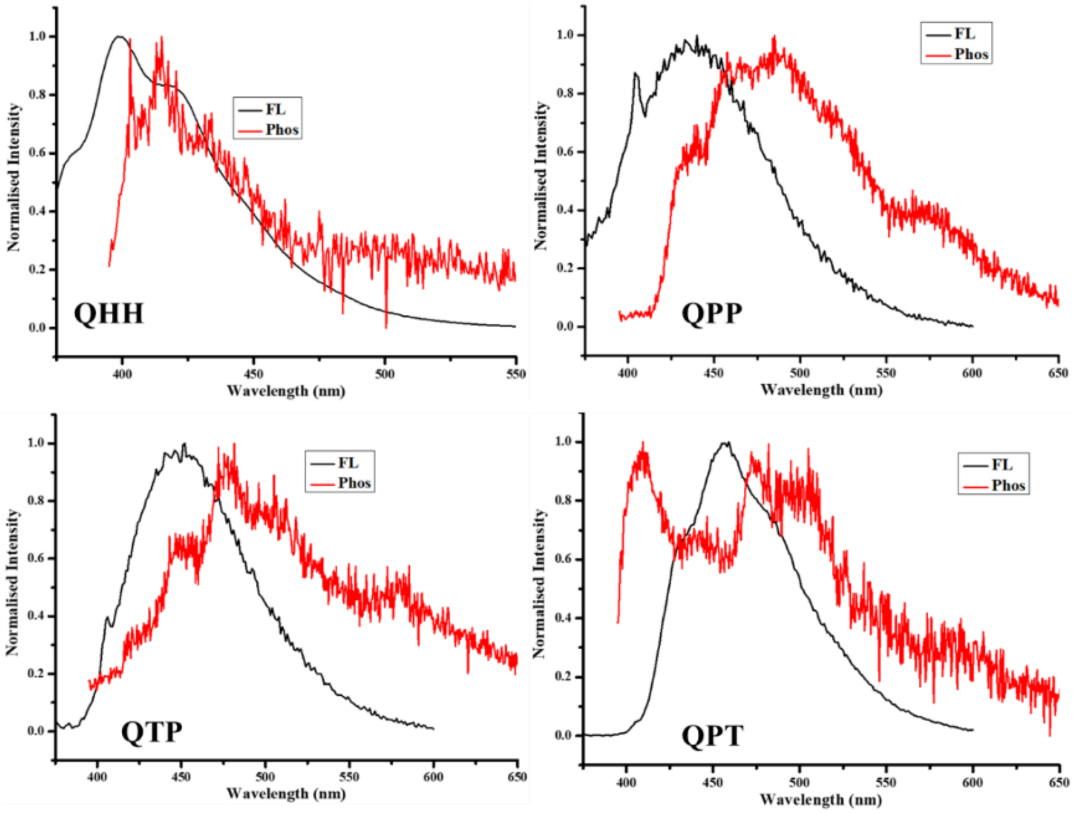

Fig. S67. Fluorescence and phosphorescence emission spectra of QHH, QPP, QPT, and QTP.

ROS generation studies

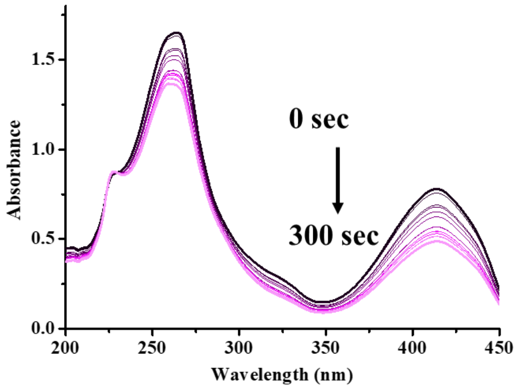

Fig. S68: UV-visible spectra of DPBF (25μM) photodegradation in dioxane using white light irradiation.

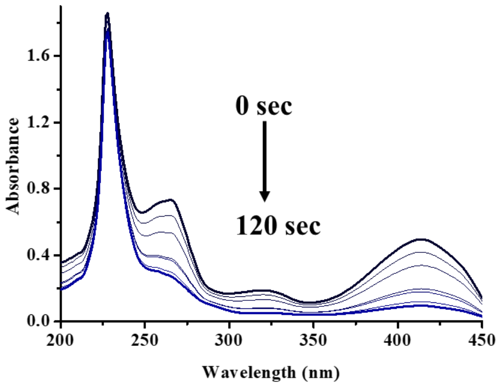

Fig. S69: UV-visible spectra of DPBF (($25\mu M$) with QPP ($5\mu M$) using white light irradiation.

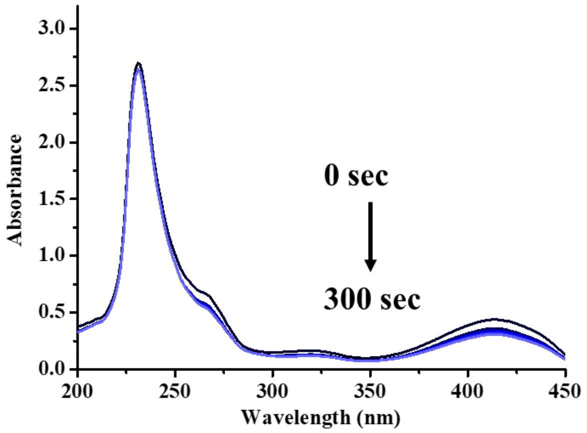

Fig. S70: UV-visible spectra of DPBF (($25\mu M$) and NaN_3 ($50\mu M$)with QPP ($5\mu M$) using white light irradiation.

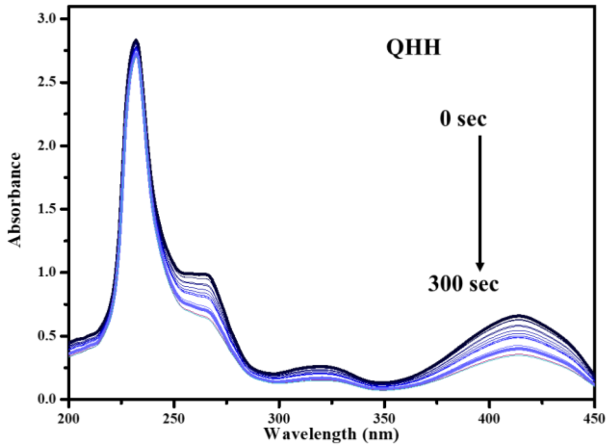

Fig. S71: UV-visible spectra of DPBF ($25\mu M$) in the presence of QHH ($5\ \mu M$) irradiating with white light source**.**

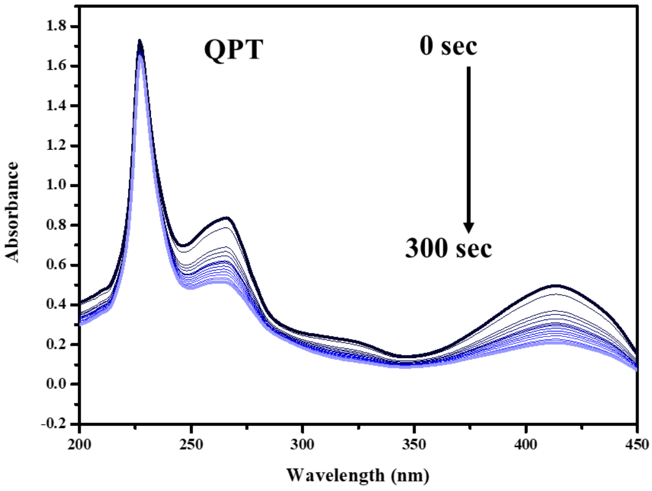

Fig. S72: UV-visible spectra of DPBF (25μM) in the presence of QPT (5 μM) irradiating white light source.

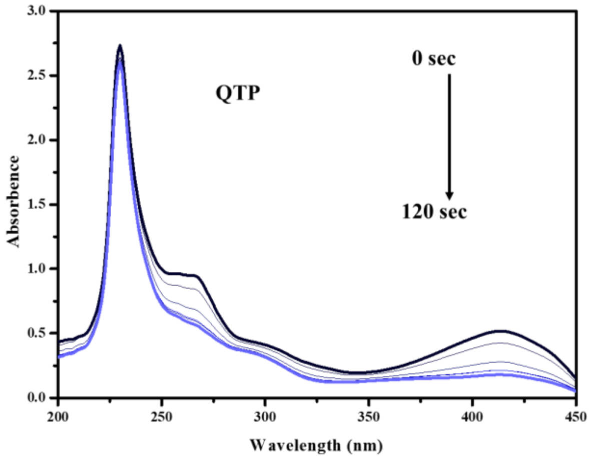

Fig. S73: UV-visible spectra of DPBF (25μM) in the presence of QTP (5 μM) irradiating white light source.

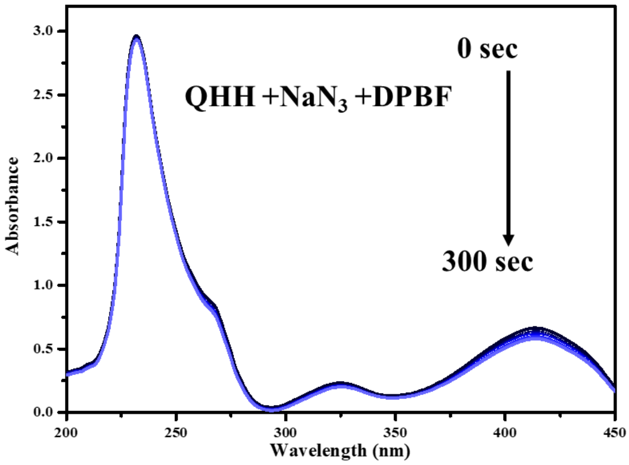

Fig. S74: UV-visible spectra of DPBF (25μM) in the presence of QHH (5 μM) and singlet oxygen quencher irradiating white light source.
